# Convergent adaptation of the genomes of woody plants at the land–sea interface

**DOI:** 10.1093/nsr/nwaa027

**Published:** 2020-02-20

**Authors:** Ziwen He, Shaohua Xu, Zhang Zhang, Wuxia Guo, Haomin Lyu, Cairong Zhong, David E Boufford, Norman C Duke, The International Mangrove Consortium, Suhua Shi

**Affiliations:** State Key Laboratory of Biocontrol, Guangdong Provincial Key Laboratory of Plant Resources, Key Laboratory of Biodiversity Dynamics and Conservation of Guangdong Higher Education Institutes, School of Life Sciences, Sun Yat-Sen University, Guangzhou 510275, China; State Key Laboratory of Biocontrol, Guangdong Provincial Key Laboratory of Plant Resources, Key Laboratory of Biodiversity Dynamics and Conservation of Guangdong Higher Education Institutes, School of Life Sciences, Sun Yat-Sen University, Guangzhou 510275, China; State Key Laboratory of Biocontrol, Guangdong Provincial Key Laboratory of Plant Resources, Key Laboratory of Biodiversity Dynamics and Conservation of Guangdong Higher Education Institutes, School of Life Sciences, Sun Yat-Sen University, Guangzhou 510275, China; State Key Laboratory of Biocontrol, Guangdong Provincial Key Laboratory of Plant Resources, Key Laboratory of Biodiversity Dynamics and Conservation of Guangdong Higher Education Institutes, School of Life Sciences, Sun Yat-Sen University, Guangzhou 510275, China; State Key Laboratory of Biocontrol, Guangdong Provincial Key Laboratory of Plant Resources, Key Laboratory of Biodiversity Dynamics and Conservation of Guangdong Higher Education Institutes, School of Life Sciences, Sun Yat-Sen University, Guangzhou 510275, China; Hainan Dongzhai Harbor National Nature Reserve Administration, Haikou 571129, China; Harvard University Herbaria, Cambridge, MA 02138-2094, USA; Centre for Tropical Water and Aquatic Ecosystem Research, James Cook University, Townsville, QLD 4811, Australia; Members of the Consortium of 26 Institutions; State Key Laboratory of Biocontrol, Guangdong Provincial Key Laboratory of Plant Resources, Key Laboratory of Biodiversity Dynamics and Conservation of Guangdong Higher Education Institutes, School of Life Sciences, Sun Yat-Sen University, Guangzhou 510275, China

**Keywords:** convergent evolution, genome, mangrove, adaptive evolution, woody plants

## Abstract

Sequencing multiple species that share the same ecological niche may be a new frontier for genomic studies. While such studies should shed light on molecular convergence, genomic-level analyses have been unsuccessful, due mainly to the absence of empirical controls. Woody plant species that colonized the global tropical coasts, collectively referred to as mangroves, are ideal for convergence studies. Here, we sequenced the genomes/transcriptomes of 16 species belonging in three major mangrove clades. To detect convergence in a large phylogeny, a CCS+ model is implemented, extending the more limited CCS method (convergence at conservative sites). Using the empirical control for reference, the CCS+ model reduces the noises drastically, thus permitting the identification of 73 convergent genes with *P*_true_ (probability of true convergence) > 0.9. Products of the convergent genes tend to be on the plasma membrane associated with salinity tolerance. Importantly, convergence is more often manifested at a higher level than at amino-acid (AA) sites. Relative to >50 plant species, mangroves strongly prefer 4 AAs and avoid 5 others across the genome. AA substitutions between mangrove species strongly reflect these tendencies. In conclusion, the selection of taxa, the number of species and, in particular, the empirical control are all crucial for detecting genome-wide convergence. We believe this large study of mangroves is the first successful attempt at detecting genome-wide site convergence.

## INTRODUCTION

Genomic sequencing has been highly successful in revealing the biology of species that are not considered suitable experimental subjects [1–7]. The next phase of genomic studies may be on species that evolve in the shared environment. Convergent emergence of phenotypes facilitating adaptation to ecologically similar environments has been extensively reported [8–10]. However, it is still unclear whether similar molecular events underlie this phenotypic convergence [11–14]. Indeed, given the complex nature of biological networks, disparate genetic pathways can lead to similar phenotypic effects. In short, convergent evolution in molecular mechanisms may not be necessary for phenotypic convergence. This has been known to be the case in human's high-altitude adaptation [15–17].

Molecular convergence can take place at several levels. For example, some consider similar selective pressures on the same genes as a form of convergent evolution. This view appears to be the basis of cancer genomic studies, which focus on tumorigenesis as phenotypic convergence [18,19]. In this study, we will consider a new form of molecular convergence—the preference or avoidance for the same amino acids (AAs) across the genomes. Nevertheless, among all forms of molecular convergence, the most commonly accepted, and the most stringently defined, is site convergence, whereby the same AA site independently evolved toward the same AA.

The investigations of site convergence fall into two categories [20]. In the genic approach, there is prior knowledge about the candidate genes or pathways underlying the convergent phenotype (see Supplementary Table 1 of He *et al.* [20]); hence, the results are generally statistically robust. In contrast, the genomic approach aims at finding signals of convergence broadly in the genome without a set of candidate genes. In the literature, the failing of the genomic approach has been the lack of proper control. Without estimating the background convergence in the control taxa, most studies estimate the noise level by simulations. Among the 14 genomic studies of convergence (Table 1 of He *et al.* [20]), only

two are associated with an empirical control. Importantly, in both the echolocating mammals [21–23] and the marine mammals [24], the empirical control showed the background convergence to be as high as (or higher than) the observed level in the focal group. At present, the genomic approach has failed to find true signals of site convergence [20].

**Table 1.** Genomes of mangroves and their non-mangrove relatives.

Taxa	No. of sequences	Data type	Data size	Sources
***Avicennia* and relatives** (red letters denote mangrove taxa)				
***Avicennia marina* var. *marina***	1	Genome (SMRT^a^)	15.7 Gb	This study
	1	Genome (Hi-C^b^)	37.8 Gb	This study
	37	Genome	79.6 + 159.6 Gb	This study
*A. marina* var. *australasica*	6	Genome	36.8 Gb	This study
*A. marina* var. *eucalyptifolia*	6	Genome	46.5 Gb	This study
*A. officinalis*	1	Transcriptome	3.62 Gb	This study
*Mimulus guttantus*	1	Genome	–	Hellsten *et al.* [27]
*Sesamum indicum*	1	Genome	–	Wang *et al.* [28]
*Acanthus* and relatives				
*Acanthus ilicifolius*	1	Transcriptome	4.45 Gb	Yang *et al.* [29]
*Ac. leucostachyus*	1	Transcriptome	4.69 Gb	Yang *et al.* [29]
**Rhizophoreae and relatives**				
***Rhizophora apiculata***	1	Genome (SMRT)	16.2 Gb	Xu *et al.* [1]
	11	Genome	89.3 + 87.0 Gb	This study
*R. mucronata*	27	Genome	15.2 + 106.6 Gb	This study
*R. stylosa*	18	Genome	15.8 + 63.0 Gb	This study
*R. mangle*	1	Genome	15.0 Gb	This study
*Bruguiera gymnorhiza*	1	Genome (SMRT)	33.5 Gb	This study
	1	Genome (Hi-C)	91.2 Gb	Li *et al.* (by personal communication)
*Kandelia obovata*	1	Transcriptome	2.33 Gb	Guo *et al.* [31]
*Ceriops tagal*	1	Transcriptome	4.31 Gb	Yang *et al.* [30]
*Pellacalyx yunnanensis*	1	Transcriptome	4.01 Gb	Yang *et al.* [30]
*Carallia brachiata*	1	Transcriptome	2.46 Gb	Guo *et al.* [31]
*Populus trichocarpa*	1	Genome	–	Tuskan *et al.* [5]
***Sonneratia* and relatives**				
***Sonneratia alba***	1	Genome (SMRT)	28.4 Gb	This study
	34	Genome	100.8 + 131.0 Gb	This study
***S. caseolaris***	1	Genome	72.3 Gb	This study
*S. apetala*	1	Transcriptome	2.46 Gb	This study
*S. ovata*	1	Transcriptome	2.32 Gb	This study
*Eucalyptus grandis*	1	Genome	–	Myburg *et al.* [3]
*Trapa bispinosa*	1	Transcriptome	7.00 Gb	Li *et al.* [32]
*Duabanga grandiflora*	1	Transcriptome	5.08 Gb	Li *et al.* [32]
*Lagerstroemia speciosa*	1	Transcriptome	2.41 Gb	This study
**Out-group**				
*Oryza sativa*	1	Genome	–	Ouyang *et al.* [92]

^a^PacBio single-molecule real-time (SMRT) sequencing.

^b^High-throughput chromosome conformation capture techniques.

In addition to the methodological issues, the ecology of the focal groups is also crucial. The ideal candidates would be a group of species that invade the same new habitat, utilize the same resources and, hence, experience the same selective pressure. Woody plants that colonize the interface between land and sea on the global tropical coasts, collectively referred to as mangroves, may be the ideal choice for the following reasons. The intertidal environments are considered extreme for woody plants where salinity, UV intensity, temperature and sedimentation are all drastically altered [25]. These physical characteristics are similar across the tropical coasts [26]. The main characteristic of the new habitat for mangroves is the saline environment that oscillates daily with the rise and fall of the tides. This ambient salinity would impact the cellular environments in mangroves. Thus, genomic convergence, both site convergence and AA-usage convergence, can be reasonably expected among mangroves.

## RESULTS

### Genomic sequencing of the component species of the mangrove guild

**Figure 1.**
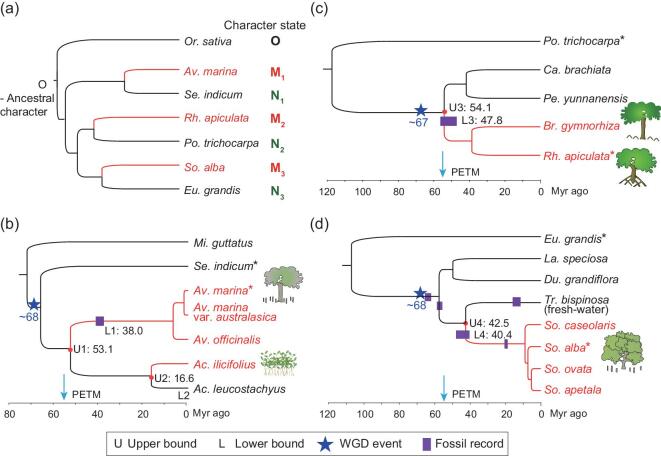
Timing and origins of the three major mangrove clades. (a) A simplified (and symmetric) phylogeny between mangroves and inland plants, represented by one species in each taxon. Mangrove and non-mangrove lineages are indicated by red and black colors, respectively. This symmetric design facilitates the detection of convergence and permits noise estimation. By using the CCS method [37], mangrove convergence is inferred only at conservative sites where all three non-mangrove species shared the same character as the out-group; i.e. N_1_ = N_2_ = N_3_ = O. The convergent signal is identified when at least two mangroves share the same derived character at a conservative site. For the control, the same criteria, with mangroves and non-mangroves switched, are applied. (b–d) The three main mangrove clades (*Avicennia*, Rhizophoreae and *Sonneratia*) of panel (a) are shown separately with detailed timing of various events. Species marked by an asterisk correspond to those of panel (a). Stars on the branches denote the timing of whole-genome duplications (see text). Solid boxes indicate the estimated age of fossils. The timing of mangrove origin is placed between Ui and Li (i = 1–4 for the four clades shown) where Ui and Li can be either a common ancestor (e.g. U1) or a dated fossil (e.g*.* L1). The fossil ages are: L1 (Middle Bartonian, 38–41.3 Myr ago) [35], L3 (47.8–56 Myr ago) [90], L4 (40.4–48.6 Myr ago) [91], *Sonneratia* (19 Myr ago) [91] and *Trapa* (11.6–15.9 Myr ago) [91]. The origins of the three major mangrove clades are between 43 and 54 Myr ago, roughly following a period of high sea levels. The PETM (Paleocene-Eocene Thermal Maximum at ∼55 Myr ago) [36] period is indicated by an arrow. See Supplementary Figs 13–15 for more details. The abbreviations for genera are as follows: *Or*, *Oryza*; *Mi*, *Mimulus*; *Se*, *Sesamum*; *Av*, *Avicennia*; *Ac*, *Acanthus*; *Po*, *Populus*; *Ca*, *Carallia*; *Pe*, *Pellacalyx*; *Br*, *Bruguiera*; *Rh*, *Rhizophora*; *Eu*, *Eucalyptus*; *La*, *Lagerstroemia*; *Du*, *Duabanga*; *Tr*, *Trapa*; *So*, *Sonneratia.* The drawings of trees are by Deirdre Bean.

In this study, we sequenced the genomes of the major component species of the mangrove guild (see Fig. 1 and Table 1). The three major mangrove taxa [*Avicennia*, *Sonneratia* and Rhizophoreae (a tribe that includes four exclusively mangrove genera: *Bruguiera*, *Ceriops*, *Kandelia* and *Rhizophora*)] together comprise 32 species, or 40% of all mangroves in the world. A fourth independently evolved lineage of mangrove is a small group in *Acanthus*, nested in the *Avicennia* clade. In total, 16 mangrove species are subjected to the genomic analyses in this study.

These genomes are done at one to three levels of completeness—the third-generation SMRT (‘single-molecule real-time’) long reads, the second-generation Illumina short reads and transcriptome sequencing (Illumina short reads). At least one species from each of the three taxa is chosen for SMRT sequencing. They are *Sonneratia alba* (SA), *Avicennia marina* (AM), *Rhizophora apiculata* (RA) and *Bruguiera gymnorhiza* (BG). The assemblies show high accuracy and completeness. The genome annotations and other information are given in Supplementary Note, Supplementary Figs 1–12 and Supplementary Tables 1–13.

Additional species of each clade were sequenced to expand the phylogeny and increase the power of detecting genomic convergence. With multiple mangrove-genome sequences from three clades (all sequenced by the International Mangrove Consortium), convergence at multiple levels can be analysed in parallel. Genomes of the non-mangrove relatives reported in the literature [3,5,27,28] with some additions by our previous studies [29–32] were also used in the analyses (Table 1).

### Independent and concurrent emergence of mangroves

As stated in the ‘Introduction’ section, the parallel transitions to similar tropical intertidal habitats may predispose mangroves to genomic convergence. It is equally important, albeit less appreciated in previous studies, that the taxa should have comparable evolutionary histories as well. The focal taxa should ideally have been in similar environments more or less concurrently, thus permitting the same amount of time for traits of convergence to evolve. This point has not been a concern in previous studies (see Table 1 of He *et al.* [20]). If two taxa invaded similar environments independently, say 1 and 5 million years (Myr) ago, they should not be expected to yield comparable convergent signals at the genomic level. In particular, when the new environments have not been constant (such as in the last 5 Myr of fluctuating sea levels [2]), the two taxa cannot be said to have been evolving in ‘similar environments’. Therefore, the search strategy should be optimized to increase the chance of detecting convergence signals, and this section provides a proper phylogenetic framework for convergence studies.

The three mangrove clades belong in three divergent lineages of angiosperm (orders Lamiales, Malpighiales and Myrtales) and, according to fossil dating, may have diverged for more than 100 Myr [33]. Genomic sequences confirm their independent origins (Fig. 1a; see Supplementary Table 14 and Supplementary Note for details). For estimating the time of mangrove emergence, a separate analysis was performed for each order (Fig. 1b–d). Each time-depth estimate is bracketed by an upper (U) and a lower (L) bound. U designates the divergence time between mangroves and their closest non-mangrove relatives and L indicates the most recent common ancestor of extant mangroves within each clade. Both estimates are obtained from the genomic or transcriptomic sequences using the MCMCTREE program of the PAML package [34]. When available, fossil dating is used in place of either U or L, thus narrowing the bracket.

In Fig. 1b, mangrove genus *Avicennia* is placed between U1 (at 53.1 Myr ago) and the common ancestor of *Avicennia* (at 6.8 Myr ago). In this case, a fossil dated to Middle Bartonian (Middle Eocene; 38–41.3 Myr ago) [35] shows traits of *Avicennia* and provides a better estimate of L1. The origin is therefore placed between 53 and 38 Myr ago (Supplementary Fig. 13). The dating of other clades, marked U2-L2, U3-L3 and U4-L4, is done by the same approach as shown in Fig. 1c and d (see Supplementary Note, Supplementary Figs 13–16 and Supplementary Tables 15–20). The origins of the three main taxa of mangroves, which together represent about half of the extant ‘true mangroves’, are clustered in the interval of 43–54 Myr ago. This interval roughly corresponds to a brief period of extreme global warming called the Paleocene-Eocene Thermal Maximum (PETM), ∼55.5 Myr ago [36]. During PETM, the eustatic sea level rose due to the melting of ice sheets. As the sea level rises, some woody plants may have developed special characteristics, such as vivipary and salt/anoxia tolerance, to cope with the increasingly saline habitat.

Prior to colonization of the new habitats, the three mangrove clades independently experienced whole-genome duplication (WGD; marked with a star in Fig. 1b–d). Indeed, the AM genome harbors 835 syntenic blocks, SA has 706 and RA has 377 syntenic blocks, accounting for between 74% and 91% of their genomes (Supplementary Fig. 17). Using nucleotide substitution numbers between genes in these syntenic blocks as autopolyploidy, we estimate that all three whole-genome duplications occurred in the same time frame between 67 and 68 Myr ago (Fig. 1b–d) and preceded habitat shifts in every case (Supplementary Figs 13–15). It seems plausible that the dual conditions of PETM and WGD may have predisposed mangroves to evolve in convergence at the genomic level.

### Two levels of convergence

We now use the collection of independently evolved mangrove genomes (Table 1 and Fig. 1) for studying genomic convergence. Convergence is analysed at two levels. First, the same sites of the same gene across species are compared (site convergence). Second, the usages of the 20 AAs across all sites of all genes are compared (usage convergence). This second approach is extended to comparing the 190 (= 20 × 19/2) possible substitutions among the 20 AAs. While the former analysis is more commonly practiced, the latter may be closer to the core adaptations of mangroves in the tropical intertidal environments as detailed in ‘Convergent evolution in AA usage’ section. This level of convergence also permits detailed studies between closely related mangroves, while previous sections compare mangroves with their distant non-mangrove relatives (‘The evolutionary mechanism of convergence observed between closely related species‘).

### Convergence at AA sites

In this section, we are able to prove for the first time site convergence at the genomic levels for the following reasons. First, the divergence depth maximizes the historical sharing of environments (section ‘Independent and concurrent emergence of mangroves’). Second, a large number of species is used to reduce the background noises. Therefore, when a new non-mangrove species shows the mangrove character, or when an additional mangrove species fails to do so, the noisy site is exposed. The number of species is important in ‘de-noising’ (see Supplement). Third, since site convergence is statistically inferred, the probability of true convergence (*P*_true_) should be presented, especially when *P*_true_ < 0.5. We are able to calculate and maximize *P*_true_ by extending the empirical CCS (convergence at conserved sites) method [37] with computer simulations. The CCS method is a symmetric design that pairs each focal species (e.g. a mangrove) with a control species (a non-mangrove relative). Let the level of observed convergence among the focal taxa be A (for all) and the observed convergence among the control taxa be N (for noise). Then, the level of true convergence among the focal taxa should be (A – N)  = S (for signal) and *P*_true_ = S/A.

Note that the CCS method is a strictly empirical test as the calculation of *P*_true_ = (A – N)/A requires only A and N, both empirically obtained. As shown in the ‘Introduction’ section, whenever the theoretically calculated false convergence in publications is replaced by the empirical value, *P*_true_ is reduced to 0. It is therefore proposed that, in any genomic study of site convergence, the starting point should be a set of genes with *P*_true_ > 0. While the CCS method provides such a set, it cannot use the full data to maximize *P*_true_ due to the symmetry design. In Xu *et al.* [37], only 3 pairs of taxa (out of a total of 21) could be used and the resultant *P*_true_ is only 0.42 for the genes identified. To improve *P*_true_, we now propose an expanded CCS+ model, briefly described below (see the ‘Methods’ section for details).

The first step of the CCS+ model uses the largest symmetric phylogeny (LSP) possible by pairing a key species from each focal taxon with an available non-mangrove relative. (This step is equivalent to the original CCS model.) In the first step, the use of conservative sites would reduce the background noises. It is also possible that sites conserved in the old environment are more likely to evolve in the new environment by convergence. In the second step, all available mangrove and non-mangrove species are added to the LSP to form a full phylogeny. In the full phylogeny, convergence is more stringently defined as follows: (i) newly added mangrove species must have the same convergent characters; (ii) newly added non-mangroves are not permitted to have the mangrove characters. In the second step, the full phylogeny permits maximal de-noising.

As a result, both S and N, termed S’ and N’ in the full phylogeny, become smaller. In the full phylogeny, A’ = S’ + N’ is observable from the expanded data and N’ can now be simulated as shown in the ‘Methods’ section. We then obtain *P’*_true_ = S’/A’. Using the CCS+ model on a large phylogeny, it should be possible to obtain *P’*_true_ = S’/A’ > 0.9, meaning that >90% of the identified genes are true convergent genes.

**Figure 2.**
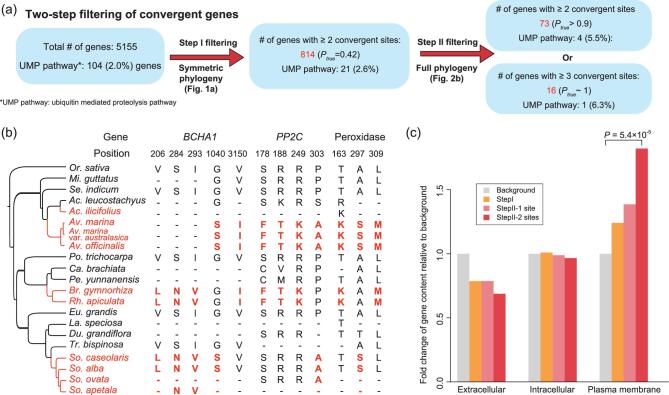
Genic convergence among mangroves. (a) A two-step procedure for identifying genes of convergence. In Step I, 814 candidate genes are identified based on the small phylogeny of Fig. 1a. A modest probability of true convergence (*P*_true_ = 0.42) is attained. In step II using the full phylogeny of panel (b), further screening of the 814 genes yields 73 genes of high confidence. Genes of the ‘ubiquitin-mediated proteolysis’ pathway are enriched in the sets. (b) Three examples of convergent genes in mangroves are shown. These genes have at least three convergence sites and are associated with salinity tolerance. Red coloring in the phylogenetic tree is used for mangrove species. (c) Proteins of convergence are enriched on plasma membrane. Subcellular localization is classified as extracellular, intracellular or membrane-bound based on the prediction of CELLO [47]. In each class, the percentage of the 5155 background genes is normalized as 1 while the percentages for the convergent genes are shown as the fold change relative to the background. For genes producing membrane-bound proteins, the enrichment of convergent genes, relative to the background, increases as the criteria become more stringent (*P* = 5.4 × 10^−5^ by Fisher's exact test). Such a pattern is not observed for extracellular and intracellular proteins.

For mangroves, Step I of the CCS+ method identifies 814 (N + S) convergent genes with S = 342 and N = 472. Hence, *P*_true_ = 0.42 [(814 – 472)/814; see Fig. 2a and Xu *et al.* [37]]. With the full phylogeny of Fig. 2b and the criteria of convergence given in the ‘Methods’ section, we obtain in Step II A’ = S’ + N’ = 73. While the total number of genes is reduced to <10% (from 814 to 73), the reduction in N’/N, which can be simulated (see the ‘Methods’ section), is even more drastic at 0.67%. This means that N’ = 472 × 0.67% = 3.16, thus yielding *P’*_true_ = S’/A’ = (A’ – N’)/A’ = 0.957. In short, the probability that each of the 73 genes is a true convergent gene is 95.7%. It is the highest rate obtained so far. Their ontologies are annotated in Supplementary Table 21. Based on the results of PROVEAN [38], 22 of the 73 genes contain at least 1 convergent site that is also highly conserved among the >50 inland species.

#### Ontology of genes of convergence

At the pathway level, ‘ubiquitin-mediated proteolysis’ stands out (Supplementary Table 22). Genes of this pathway facilitate cellular tolerance to environmental stimuli by modulating downstream transcription factors [39]. Genes of this pathway were enriched with mangrove convergent genes, with four genes carrying no fewer than two convergent sites annotated in this pathway (5.5%, 4/73) vis-à-vis 2.0% (104/5155) for the whole data set (*P*-value = 0.059, Fisher's exact test). The four genes are *DDB1a*, *APC7* (anaphase-promoting complex subunit 7) and two genes of *FBXW7* (F-box and WD-40 domain protein 7). The details of the four genes are described in Supplementary Table 23.

An even more stringent cut-off of ≥3 convergence sites per gene yields 16 genes. Interestingly, 3 of the 16 genes are involved in salinity tolerance, including *PP2C*, *BCHA1* and peroxidase, as displayed in Fig. 2b. PP2C (protein phosphatase 2C) is essential for abscisic-acid signaling, which functions in stress response [40,41]. BCHA1 (BEACH-domain homolog A1) is essential for salt-stress tolerance, thanks to its regulation of the mRNA-processing body [42]. Peroxidase and other antioxidants scavenge reactive oxygen species, which are induced by stresses and can alter normal cellular metabolism through oxidative damage to the cellular components [43].

The analyses of genes and pathways point to the cellular environment as the main setting in which the adaptive pressure is exerted. Proteins of mangroves have to be adapted to the new cellular environment, which reflects the salinity fluctuation in the ambience. In this context, the subcellular localization of proteins of convergence should be informative. We thus classify genes of convergence as extracellular, intracellular or membrane-bound, as shown in Fig. 2c. It is clear that the convergent genes are enriched on the plasma membrane and the higher the stringency in calling convergence, the greater the enrichment (Fig. 2c). For example, 35 of the 73 genes with 2 or more convergent sites could be localized on the plasma membrane (48%), which is significantly higher than that of the background (26%; *P*-value = 5.4 × 10^−5^, Fisher's exact test). At the genic level, membrane proteins experience convergence. In the following sections, we address the molecular events underlying intracellular adaptation.

### Convergent evolution in AA usage

From the site-convergence analysis, we conclude that the pathways involved in convergence mainly govern cellular processes. In other words, the relevant environmental factors appear to be within the cells. In their natural habitats, mangroves cannot maintain constant salinity in all tissues [44,45] because the salinity concentration in the intertidal zones fluctuates daily as the tides ebb and flow. In a stable saline environment, the cytoplasmic salinity concentration of mangrove cells is comparable with plants of non-saline habitats [44]. However, when the salinity changes, it would take several days for the mangrove cytosol to re-equilibrate [44]. Therefore, in the natural habitats of mangroves, the cellular level of salinity likely fluctuates as well. We turn inward to see whether and how the proteins of mangroves evolve in response to these cellular conditions.

**Figure 3.**
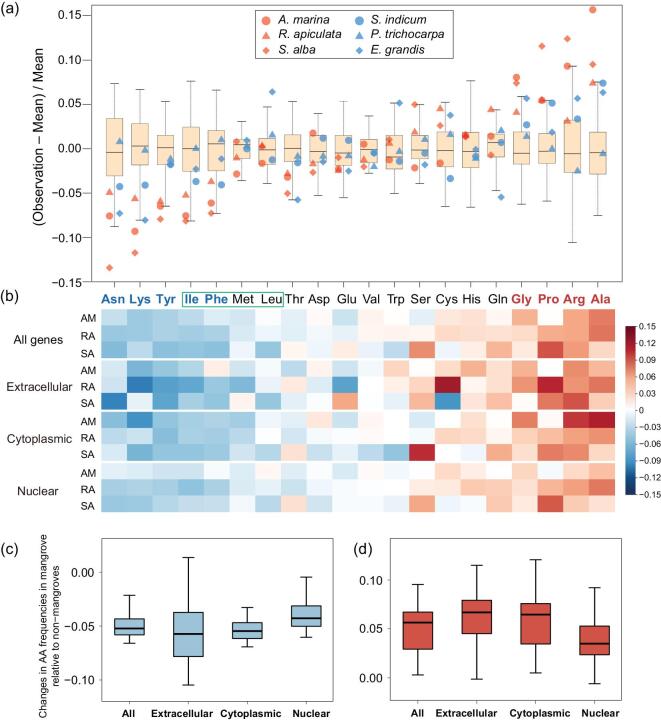
Convergence in amino acid (AA) usage in mangroves. (a) AA compositions of 57 dicotyledon genomes are used as the reference. The percentages of all AAs in each genome are shown against the distributions of all species, displayed by the box plot. The three mangroves and their closest inland relatives are shown by red and blue dots, respectively. The five most underused AAs are on the left and the four most overused AAs in mangroves are shown on the right. In addition, the four AAs, namely Ile, Phe, Met and Leu (boxed by the green border), are large hydrophobic residues that could potentially destabilize proteins in hypersaline conditions (see text). (b) AA-usage changes in different subcellular locations. The heat intensity is measured as shown in the *y*-axis of panel (a). The extracellular proteins show slightly more deviations from the reference genomes than proteins located in the cytoplasm. Both show greater deviations than those located in the nucleus, as expected if AA compositions evolve to respond to the local salinity. (c) and (d) Box-plot representation of the pattern of (b). The blue and red box displays, respectively, the five underused and four overused AAs in three mangroves.

We first compare the AA compositions of mangroves with those of 54 other dicotyledonous plants. As shown in Fig. 3a, AA usages in mangroves are consistently the outliers among plants surveyed. Nine AAs, shown in colored letters, meet the two criteria: (i) all mangrove species are above the third quartile or below the first quartile among the 57 species; and (ii) at least one of the mangroves is in the top or bottom 10%. In every case, the AA that meet the two criteria also satisfies the third one: (iii) the AA usage of each mangrove is more extreme than that of its closest non-mangrove relative (*P* < 0.01, chi-square test). Among the nine AAs, four are overused (red font) and five (green font) are underused (Supplementary Fig. 18). Furthermore, Ile and three additional AAs (the green border box) have large hydrophobic residues (Supplementary Fig. 19). In hypersaline conditions, their non-specific inter- and intramolecular interactions may break the proper folding and conformation of proteins [46].

To test the statistical significance of the deviations, we resample the AA usage of the non-mangrove species for three hypothetical mangrove taxa (see the ‘Methods’ section). Because the increase in any AA usage would result in decreases in others, all AA samplings are weakly interdependent; hence, extensive simulations are necessary. We first ask how significant it is to have five AAs that are underutilized in all three simulated mangroves by the criteria (i) and (ii) above. The simulated resampling shows that the probability of having five or more underutilized AAs is 3.3 × 10^−4^ if the underlying usages are the same as non-mangroves. Assuming five unpreferred AAs by mangroves, we now ask whether the four overutilized AAs as shown in Fig. 3a are also significant. The question arises because, although mangroves avoid certain AAs, they may not prefer any others and the appearance of overutilizations in those four AAs is not biologically meaningful. This possibility again is rejected, with *P* = 3.2 × 10^−3^. In short, mangroves as a group prefer some AAs and also avoid others, relative to their non-mangrove relatives.

Since proteins are distributed in the different subcellular locations, the hypersaline conditions may affect the AA usages as a function of these locations. Here, we use CELLO, a commonly used subcellular localization predictor [47], to assign the protein location. Figure 3b–d shows the AA-usage changes for proteins in extracellular, cytoplasm and nuclear locations in mangroves vis-à-vis their closest non-mangrove relatives. For the nine most significantly changed AAs, the degree of change is highest in the extracellular and lowest in the nuclear location. This pattern indicates the degree of deviation in AA usage to correspond with the local salinity level.

Among published genomic sequences, the genomes of mangroves appear to be the only ones that are enriched in the GC content only at non-synonymous sites. It is noteworthy that the four most commonly used AAs (red font in Fig. 3a) are coded by GGN, CCN, CGN and GCN, for Gly, Pro, Arg and Ala, respectively. Hence, the GC content of the coding region is increased in all three mangrove taxa compared to their inland relatives (by 0.91%, 1.96% and 1.54%, respectively; Supplementary Table 24). The trend is absent on 4-fold degenerate sites and in introns (Supplementary Note, Supplementary Tables 24 and 25), indicating that the selective pressure acts on AA (coding regions) rather than the nucleotide level of whole-genome sequences.

It should be further noted that AAs with GC-rich codons are energetically less costly [48]. In mangroves, the HEB scores [high-energy bond; the number of high-energy phosphate bonds (∼PO_4_) required to synthesize each AA] [49–51] of the more commonly used AAs are significantly smaller than the less common ones and the mean HEB scores of all mangroves are smaller than those of their inland relatives (Supplementary Fig. 20). Lower energetic cost could be part of the adaptive strategy in the intertidal soils that have extremely low nutrient availability [52].

### The evolutionary mechanism of convergence observed between closely related species

The convergence in AA usage reported in Fig. 3 could be driven by different evolutionary mechanisms to reach the same end. Consider the simplest case of two AAs. Let the evolutionary rate of AA1 → AA2 be f1 and the reversal rate be f2. At equilibrium, AA1/AA2 is determined by f2/f1 but the same AA1/AA2 ratio can be achieved by different f1’s and f2’s. When we consider the 20 AAs, there would be 380 (20 × 19) substitution rates. These rates are functions of the biochemical properties of AAs [53,54] and they would collectively determine the relative abundance of AAs.

Measurements of AA-substitution rates are well established [54,55] and have been further extended recently [56]. Instead of the 380 rates, the measurements usually yield 190 rates, since (AAi → AAj) and (AAj → AAi) are not distinguishable. We rank them by their relative magnitude from K1 to K190 in descending order. Between closely related species, K1–K75 > 0 and K76–K190 ∼ 0 because the latter require 2- or 3-bp changes [54,55]. Ki's are readily obtainable using available packages [34,55]. The rank order of Ki's is nearly constant across a wide range of species from *Drosophila*, primates to yeast and rodents [54]. The constancy, determined by the physico-chemical properties of the AAs, permits the calculation of the expected Ki's [E(Ki)’s] between any pair of closely related species [57].

**Figure 4.**
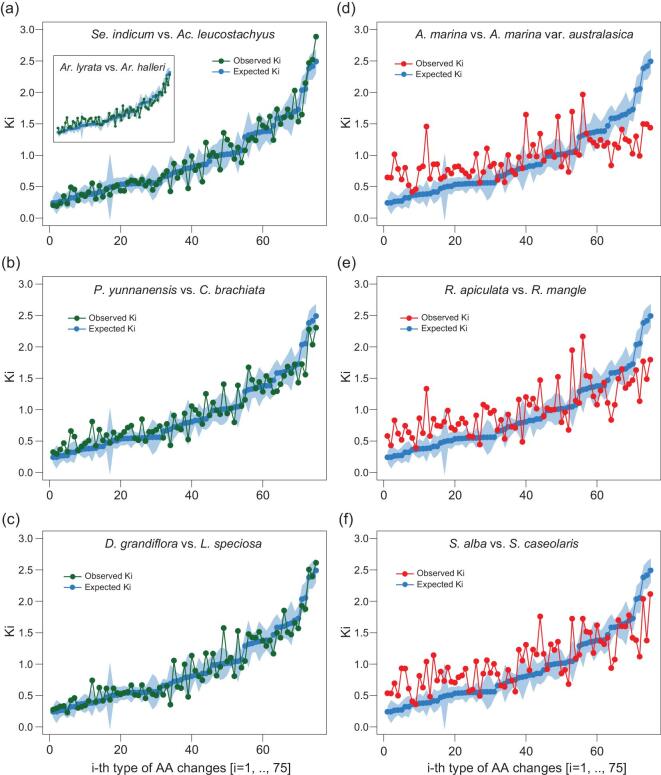
Observed vs. expected Ki's in mangroves and their non-mangrove relatives. The expected Ki's based on the universal index [54] across primates, rodents, yeast and *Drosophila* are shown in each panel (the line of blue dots; the shade covers one standard deviation on each side). *x*-axis: the ranking of i's from 1 to 75. *y*-axis: observed Ki and expected Ki. The observed Ki is scaled such that Ka/Ks = 1, as the universal index is scaled. (a–c) In non-mangroves, as well as in *Arabidopsis* [*A. lyrata* – *A. halleri*; see the inset of panel (a)], the observed Ki's agree well with the expected values. (d–f) In mangroves, the observed Ki's deviate strongly from the expected Ki's (see Table 2 for details).

We first compare the observed Ki's with E(Ki)’s among non-mangrove plants. A close match between the observed and the expected means the protein evolution between these plants follow the general rules of AA substitutions. Figure 4a–c shows the three comparisons between non-mangrove species, which are, respectively, the closest relatives of *Sonneratia*, *Avicennia* and *Rhizophora*. The Ki values between two closely related *Arabidopsis* species are also given in the inset of Fig. 4a. In all cases, the correlation between the expected and the observed Ki's is high, with *R*^2^ > 0.9 for all non-mangrove pairs. The results corroborate the existence of a general evolutionary mechanism governing AA substitutions. Strikingly, the patterns are very different between mangrove species. The observed Ki's (red lines in Fig. 4d–f) do not follow the E(Ki)’s well. Many values are several standard deviations away from the expected.

In general, AA changes that are relatively rare in molecular evolution tend to experience accelerated substitution in mangroves whereas a few commonly exchanged AAs experience deceleration. Among the 75 Ki's, 12 values converge in all 3 mangrove taxa (Table 2) with an average of ∼4 standard deviations from the expected. In contrast, these 12 Ki's in the non-mangrove control deviate from the expected Ki's by an average of only 0.917 standard deviations. It is clear that mangrove proteins have been evolving by a similar substitution mechanism that appears to be unique in mangroves.

**Table 2.** The unusual and convergent pattern of AA substitutions in mangroves.

AA1	AA2	*Av. marina* vs. *Av. marina* var. *australasica*	*R. apiculata* vs. *R. mangle*	*S. alba* vs. *S. caseolaris*	Average of mangrove pairs	Average of non-mangrove pairs
Asp	Tyr	4.516	3.789	3.294	3.866	0.296
Arg	Trp	6.292	4.749	3.617	4.886	0.377
Cys	Tyr	6.283	4.301	2.807	4.464	1.417
Cys	Phe	3.338	2.945	4.234	3.506	1.416
Ser	Trp	2.668	3.143	2.817	2.876	0.059
Arg	Cys	5.002	4.426	3.059	4.162	1.486
Leu	His	2.986	4.141	4.340	3.822	0.963
Ser	Cys	2.731	3.443	5.056	3.743	1.991
Arg	Lys	−3.554	−3.562	−3.083	−3.400	−0.359
Thr	Ala	−4.007	−4.948	−4.982	−4.646	−1.419
Asn	Ser	−4.234	−3.653	−3.718	−3.868	−0.767
Val	Ile	−4.209	−4.245	−4.756	−4.403	−0.450
Mean of absolute values		4.152	3.945	3.814	3.970	0.917

The numbers of standard deviations between the observed and expected Ki's are given. The 12 pairs (out of 75) deviate by >2.5 standard deviations in all three mangrove taxa.

## DISCUSSION

In this study, we sequenced a major part of a biological community, composed of species that utilize resources in similar ways, to study convergence

at the genic and genomic levels. Convergence may be viewed as the strongest manifestation of adaptive evolution, as the same adaptive path has been taken multiple times. We demonstrate that (i) a large number of well-chosen taxa are most crucial for detecting convergence (Table 1 and Fig. 1); (ii) the chosen taxa should ideally colonize the same new habitat independently and concurrently (Fig. 1); (iii) with the empirical control, site convergence can be identified with very high confidence (>90% of the reported genes; see Fig. 2); (v) high-level convergence in AA usage is central to the adaptation of a mangrove in the land–sea interface (Fig. 3); and (v) the highly unusual AA-substitution patterns between closely related mangrove species (vis-à-vis all other plants) indicate continual convergent adaptation even among present-day mangroves (Fig. 4 and Table 2).

In this study, mangroves from the same community indeed show strong evidence of site convergence. Under the conditions of maximal noise suppression, we identify 73 convergent genes that function in stress tolerance at the cellular level. It appears that the ecological pressure manifests itself in the cellular environment to drive genic convergence. Furthermore, mangroves also converge in higher-level genomic features. We have recently completed an extensive survey in which mangroves are found to have relatively small genomes, likely due to the smaller load of transposable elements (TEs) [58]. TE transposition is apparently suppressed when the cellular environments in mangroves changed.

Interestingly, in parallel with genome-size reduction, many gene families also shrink in size in mangroves (Supplementary Fig. 21). In particular, the reductions in gene families of pathogen resistance are pronounced (Supplementary Table 26 and 27). It may be possible that pathogens also found the new habitats of mangroves inhospitable (Supplementary Note). Convergent response in the transcriptome under salt stress is evident as well (Supplementary Note, Supplementary Figs 22 and 23 and Supplementary Table 28). These observations collectively support the thesis that similar cellular environments underlie the genomic convergence in mangroves.

This study of mangroves thus suggests the conditions necessary for convergent evolution. First, the external environments have to be highly similar and mangroves indeed share the tropical coastal habitats. Second, similar cellular environments may have large and immediate impacts on molecular convergence (such as AA composition). Third, molecular convergence is more likely when there are only a small number of genetic pathways, via which organisms cope with the selective pressure.

Convergence is an indication of the limited genetic options for a particular adaptation. A botanical puzzle about mangroves is the small number of species: at ∼80, it is much smaller than the number of woody plants in most other ecosystems. The need to converge on a limited number of phenotypic and genotypic states may have restricted the number of successful lineages [9]. Furthermore, such highly specialized modes of existence may also suggest a less robust system against environmental changes. Indeed, several recent massive die-offs could portend ‘a world without mangroves’ [59], as mangroves have come under the joint influences of natural and man-made disturbances [60]. The convergence in the past may thus offer a hint of the future.

## METHODS

### Genome sequencing and assembly

Materials used for whole-genome sequencing were collected in Qinglan Harbor, Hainan, China (19°37’N, 110°48’E). One mature individual of each species was randomly selected. Genomic DNA was extracted from leaves using the CTAB (hexadecyltrimethylammonium bromide) method [61] and total RNA was extracted from leaves, roots, flowers and stems using the modified CTAB method [62]. The 20-Kb SMRT long-read library were prepared following the PacBio SMRTbell 20 Kb Template Preparation BluePippin Size Selection protocol and were sequenced using the Biosciences RS II platform. Short-read libraries were constructed following the TruSeq DNA Sample Preparation Guide. Libraries with DNA fragment size of 200 bp, 300 bp, 400 bp, 500 bp, 2 Kb, 5 Kb and 10 Kb were sequenced using the Illumina Hiseq 2000 platform. Transcriptome sequencing was performed following the standard Illumina-transcriptome pipeline. The library insert size for transcriptome sequencing was 300 bp.

The SMRT long reads and Illumina short reads were combined to assemble a draft genome. Before assembling, PCR duplication, adaptor contamination and low-quality reads were filtered out. The *de novo* assembled genome based on the SMRT long reads was produced using four programs: falcon (https://github.com/PacificBiosciences/FALCON/), DBG2OLC [63], smartdenovo (https://github.com/ruanjue/smartdenovo) and wtdbg (https://github.com/ruanjue/wtdbg). The result obtained with smartdenovo was used as the final assembly because of its superior quality. To further improve site-specific consensus accuracy, Quiver [64] was used to perform genome polishing. Illumina reads were then mapped to the polished genome assembly using BWA [65]. SNPs as well as small indels were called and corrected using SAMTOOLS [66] and in-house scripts. Finally, gap-filling was performed on the scaffolds with SSPACE 3.0 [67] using 10-Kb mate-pair sequences with the key parameters set as: -x 1 -m 50 -o 10 -z 200 -p 1.

Three-dimensional proximity information was obtained by high-throughput chromosome conformation capture sequencing (Hi-C) [68] for AM and BG. We used Juicer [69] and HiC-Pro pipeline for Hi-C data processing [70].

Transcriptome data, BUSCO [71] (Benchmarking Universal Single-Copy Orthologs) genes and randomly selected genes from our previous work were used to evaluate the genome coverage and structural accuracy of the genome assembly (Supplementary Note).

### Genome annotation

The repeat sequences were masked throughout the genome using RepeatMasker (version 3.2.9) [72] and the RepBase library (version 16.08) [73]. Base on the repeat-masked genomes, homologous protein alignment, *ab initio* gene prediction and transcriptome data were combined for protein-coding gene prediction.

For homolog-based prediction, homologous proteins from five whole-genome sequences, namely *Oryza sativa*, *Mimulus guttatus*, *Sesamum indicum*, *Populus trichocarpa* and *Eucalyptus grandis*, were aligned to each of the two mangrove genomes using exonerate (v1.1.1) [74]. Based on the alignments, gene structures were generated using Genewise (version 2.2.0) [75]. The Augustus (version 3.2.2) [76] and GeneMark-ET (version 4.29) [77] algorithms were used to predict protein-coding genes *ab initio*. RNA-seq reads were mapped to the genome using Tophat (version v2.1.1) [78] and gene models from spliced transcripts were identified using cufflinks (version v2.2.1) [79]. Finally, the three sets of predicted genes were combined using EVidenceModeler (EVM) [80] to generate a weighted and non-redundant consensus set of gene structures.

To annotate the functions of genes, coding sequences were aligned against the SwissProt, TrEMBL [81] and NCBI non-redundant protein databases using BLAST (v2.2.6) with an e-value threshold of 1 × 10^−5^. Gene-ontology annotation was obtained by aligning against the Pfam database [82] using HMMER2GO (https://github.com/sestaton/HMMER2GO). KO (KEGG Orthology) assignments and pathway annotation were generated by searching against the KEGG database [83].

### Phylogenetic analysis and time dating

The genomes of SA, AM, RA, BG and *Sonneratia caseolaris*, together with the genome-sequencing data of *Avicennia marina* var. *australasica* and the transcriptomes of 10 related species, were used to calculate the divergence time for each mangrove lineage (*Sonneratia*, Rhizophoreae and *Avicennia*) (Supplementary Table 14). In each lineage, genes were clustered into families using the OrthoMCL software [84]. Phylogenetic trees for gene families were built using PhyML [85]. The program MCMCTREE of the PAML 4.8 package [34] was used to estimate the species-divergence time with the HKY85+gamma model assuming an independent rate for each branch. The detailed methods and time calibrations are described in the Supplementary Note.

To detect the signature of a WGD event**,** self-alignment was performed on protein sequences for each species using BLASTp (with an e-value cut-off of 1 × 10^−5^, identity ≥40%). The syntenic blocks were then identified using MCScanX [86]. Collinear blocks with at least five paired homologous genes were selected in this study. The results were visualized using the Circos software (v0.65) [87] for a manual check. Then, we dated the time of WGD events using the methods described in the Supplementary Note.

### Gene-family analysis

The OrthoMCL software was used to identify orthologous and paralogous groups of seven genomes (AM, RA, AS and their inland relatives *S. indicum*, *P. trichocarpa*, *E. grandis* and *O. sativa* as an out-group; Supplementary Note). For genes with alternative splicing, the longest transcripts were selected for analysis. The proteins of these seven species were merged to perform all-vs.-all alignment using BLASTp with an e-value cut-off of 1 × 10^−10^. The results were fed into a stand-alone OrthoMCL program with a default MCL (Markov Cluster Algorithm) inflation parameter of 2.0. After gene-family clustering, CAFE [88] was used to analyse the expansion and contraction of gene families among the seven species. Taking the gene-family sizes as input, CAFE used a stochastic birth-and-death process to model the evolution of gene-family sizes across a given phylogenetic tree and detected expanded or contracted gene families with *P*-value < 0.05.

### CCS+ model for inferring convergence in AA substitutions

The CCS+ model was designed to infer convergent AA substitutions with eliminating false positives in two steps. In the first step, the CCS+ model utilized the LSP possible by pairing a key species from each focal taxon with an available non-mangrove relative. With this symmetric design, the level of true convergence among mangroves could be controlled by the convergence among non-mangroves. In the second step, more mangroves and non-mangroves are added to the phylogeny. The advantage of the two-step method is the biological control in the first step, which informs the existence of true convergent genes. In the second step, the symmetry is no longer needed and it is possible to simulate the reduction (q, see below) in the noise level in this step. See Supplementary Fig. 24 for details.

In the first step, convergence was inferred according to the setting of Xu *et al.* [37]. For each of the three mangrove taxon, *A. marina*/*S. indicum*, *R. apiculata*/*P. trichocarpa*, *S. alba*/*E. grandis* were used as focal/control species pairs, respectively (Fig. 1a). And *O. sativa* was used as the out-group. Under the symmetric phylogeny, mangrove convergence is inferred only at conservative sites where all three non-mangrove species shared the same character as the out-group; i.e. N_1_ = N_2_ = N_3_ = O. At conservative sites, the ancestral state can be confidently inferred to be ‘O’, as described in Xu *et al.* [37]. With ancestors inferred as O, convergence can be inferred if two (or three) of the three mangrove species share a derived character that is different from the ancestral state, i.e. M_i_ = M_j_ ≠ O. For the control, the same criteria, with mangroves and non-mangroves switched, are applied. Genes carrying at least two mangrove (or non-mangrove) convergent sites were retained. The number of observed convergent genes among the focal mangrove taxa is A (for all) and the number of observed convergent genes among the control taxa is N (for noise). The level of true convergence among the focal taxa can be calculated as A – N = S (for signal) and *P*_true_ = S/A.

The inland relatives used in the first step are usually distantly related to mangroves. To further identify the true convergence that accompanied the habitat change and to further elevate the *P*_true_, more closely related mangrove and non-mangrove species were used. In the second step, more mangrove and non-mangrove species were added to the LSP to form a full phylogeny. In the full phylogeny, convergence was more stringently defined as follows: (i) newly added mangrove species must have the same convergent characters; (ii) newly added non-mangroves are not permitted to have the mangrove characters; (iii) the mangrove genes carried at least z convergent sites. (In practice, z is set from 1 to 4; in this application, we set z = 2.) As a result, both S and N, termed S’ and N’ in the full phylogeny, became smaller. In the full phylogeny, A’ (A’ = S’ + N’) was observable from the expanded data. N’ is equal to N*q, where q is the retention rate of noise from the first to second steps. Using the simulation procedure below, q could be estimated. Hence, *P’*_true_ could be estimated as *P’*_true_ = S’/A’ = (A’ – N’)/A’.

### Simulation of noise elimination when using more species

Sequence simulation was utilized to estimate the retained noise level (q = n’/n; we use n to denote N of the simulated result) from first to second steps of the CCS+ method. Sequence simulation was performed using *evolver* in the PAML package, according to the phylogeny of real data. Using simulated sequences, the number of convergent sites retained in the first and second steps could be calculated. Since there was no positive selection assumed in the simulation, all convergence identified was noise (n and n’ in the first and second steps). And the retention of noise could be calculated as q = n’/n. The detailed procedures were described as follows.

We first used PhyML to calculate the branch lengths (AA-substitution rate) of the full phylogeny. After that, the *evolver* program of the PAML package was utilized to produce simulated sequences. Given 21-species tree topology and branch lengths, the equilibrium AA frequency and the AA-substitution model (LG model [89]), the *evolver* program produced sequences for each node on the phylogeny. In total, 100 M AAs were produced for the following noise-elimination estimation. These AA sites were assembled into genes according to the 5155 gene lengths. Then the CCS+ step I and step II criteria were applied to identify the number of convergent genes for the simulated data set, which was n and n’, respectively. Then the retention of noise could be calculated as q = n’/n.

### Simulation of AA-usage bias

In the analyses of AA usage, we found the frequencies of 9 AAs were outliers compared with 54 inland plants. Among the nine AAs, four are overused and five are underused. To assess the biological significance, simulations were utilized to examine the by-chance probability of the observations.

First, we examined the total number of overused and underused AAs observed by chance. We randomly picked a value from 54 inland plants for each AA to form the AA composition of a pseudo-genome (the sum of 20 AA compositions was normalized to 1). In each simulation replicate, the AA compositions of three pairs of pseudo-genomes were generated and checked by the criteria (i) and (ii) (see main text). If X of 100 000 simulation replicates generated no fewer than 9 outlier AAs, the probability of our observations by chance would be X/100 000. Then, for the simulated data set, we examined the number of extremely underused AAs that could be observed on the condition that four AAs were extremely overused and the number of extremely increased AAs on the condition that five AAs extremely decreased in frequency.

### AA-substitution-rate analysis

In each group, we chose a pair of mangrove species and a pair of inland species to calculate the AA-substitution rate Ki using the *codeml* of the PAML 4.8 package [34]. The parameter -1 and aaDist = 7 was applied in the file OmegaAA.dat, which specifies independent rates for 75 pairs of one-step AA changes. The universal index was used to be the scaled expected Ki, since the rank and relative values of Ki have been proven to be stable across primates, rodents, yeast and *Drosophila* [54]. The standard deviation of the expected Ki or universal index is calculated by using the scaled Ki of eight pairs of species including four pairs used in the previous study [54], three pairs of non-mangroves in Fig. 4a–c and *Arabidopsis lyrata* vs. *A. halleri*.

## DATA AVAILABILITY

The Whole Genome Shotgun project has been deposited at DDBJ/EMBL/GenBank under the accession codes PRJEB8422 and PRJEB8424. The whole-genome sequences are also available for download at http://evolution.sysu.edu.cn/Sequences.html.

## Supplementary Material

nwaa027_Supplemental_File
