# Supplementary material for: Convergent adaptation of the genomes of woody plants at the land–sea interface
Source: Natl Sci Rev. 2020 Feb 20;7(6):978–93. doi: 10.1093/nsr/nwaa027 (PMC8289059; doi:10.1093/nsr/nwaa027)
Supplement: nwaa027_Supplemental_File [file nwaa027_supplemental_file.pdf]

Supplementary Information for

Convergent adaptation of the genomes of woody plants at the land-sea interface

Ziwen He[1], Shaohua Xu[1], Zhang Zhang[1], Wuxia Guo, Haomin Lyu, Cairong Zhong, David E. Boufford, Norman C. Duke, The International Mangrove Consortium, Suhua Shi[*]

[1] These authors contributed equally

[*] Correspondence: lssssh@mail.sysu.edu.cn (S.S.)

This file includes:

Supplementary Note

Supplementary Figs. 1-24

Supplementary Tables 1-28

Supplementary References

Supplementary Note

1. Whole-genome sequencing, assembly, and annotation

Material preparation

The three major mangrove taxa (*Avicennia*, *Sonneratia* and Rhizophoreae) together comprise 32 species, or 40 % of all mangroves in the world. We describe the collection of data from all species here since we occasionally use the whole data set to discern a trend. For example, genome size reduction is a general pattern among all mangroves we have analyzed. The genomes of species other than *Avicennia marina* (AM) and *Sonneratia alba* (SA) will be analyzed in more detail in a separate study in order to address issues that are beyond the scope of this report. These species exhibit special characteristics that are beneficial for adapting to the intertidal zone, such as vivipary, salt excretion, and special root systems [25]. Species of *Rhizophora* are typical mangroves with true viviparous seeds. *Avicennia* genus exhibit cryptovivipary, where the embryo does not break away from the fruit wall before the fruit falls off. Although *Sonneratia* are non-viviparous, their seeds germinate without dormancy unlike most other plants. *Avicennia* have the ability to secrete salt, whereas the other two genera exclude salt from entering root cells. *Avicennia* and *Sonneratia* develop pneumatophores, whereas stilt-roots occur in *Rhizophora* [25].

We randomly sampled a single mature *Avicennia marina* var. *marina* (AM), *Rhizophora apiculata* (RA), *Sonneratia alba* (SA) and *Bruguiera gymnorhiza* (BG) plant from Qinglan Harbor, Hainan, China (19°37'N, 110°48'E) for *de novo* genome sequencing and assembly. *S. caseolaris* and most other species were collected from the same place in Qinglan Harbor, Hainan. *A. marina* var. *australasica* was from New Zealand (36°52'S, 174°46'E). Genomic DNA was extracted from leaves using the CTAB method [60].

In a separate study, we obtained AM, RA, and SA samples from the Indo-Malayan coasts and southern China [2]. The samples from Hainan are usually lower in heterozygosity than those from the coasts of Thailand. The average number of nucleotide differences per Kb between two random sequences in Hainan were estimated as 0.316 in AM, 0.451 in RA, and 0.260 in SA. The respective values are 0.934, 0.586, and 0.435 in samples from Gulf of Thailand. Each data point is the average over 80 loci. We usually have 100 individuals from three to five sites in both regions.

The relevant question is whether the Hainan samples or the Thailand samples more accurately represent the diversity we are interested in. We suggest that the Hainan samples are indeed the right ones to use. In the previous study we found that all mangrove species have deep population subdivision separated by the Strait of Malacca [2]. As the sea levels rose and fell in the last 2 Myr, the Strait, being only 25 meter in depth, has been closed to gene flow most of the time. This isolation was punctuated repeatedly by gene flow when sea levels rose above -25 m. The higher diversity in samples collected near the Strait reflects gene flow from the coasts on the other side of the Strait of Malacca. Thailand samples reflect both the demographic history on one coast and the genetic input from the other coast.

The Hainan sample is also affected by this recent gene flow, but to a lesser extent since it is relatively far from the Strait. For that reason, the Hainan samples may more faithfully portray the adaptive history of the species with a fewer geographical complications of the region.

Whole-genome sequencing

In order to generate a better draft genome, we used a joint strategy of single-molecule real-time sequencing (SMRT; Pacific Biosciences, Menlo Park, CA, USA) and next-generation Illumina HiSeq 2000 sequencing (Illumina Inc., San Diego, CA, USA). The whole-genome sequencing workflow is shown in Supplementary Figs. 1-3. SMRT sequencing was conducted on a PacBio RS II sequencing platform using the C4 sequencing chemistry and P6 polymerase with 20 Kb SMRT bell library and 25 SMRT cells. For the Illumina sequencing, we constructed 10-12 libraries with a variety of insert sizes (200 bp, 300 bp, 400 bp, 600 bp, 2 Kb, 5 Kb and 10 Kb). Raw reads were sequenced on the HiSeq 2000 platform for each species. The Illumina short reads were filtered via the following steps: (1) removing reads containing the Illumina TruSeq adaptor core sequence "GATCGGAAGA" with ≤ 1 mismatch in the 3' end; (2) removing duplicated reads from PCR amplification (if read 1 and read 2 of the two paired-end reads were identical in the first 30 bp); (3) removing reads shorter than 30 bp; and (4) removing single-end reads.

As a result, we obtained 15.7 Gb (N50 length at 14.2 Kb) and 28.4 Gb (N50 length at 17.7 Kb) of SMRT long-reads (Supplementary Figs. 4-5; Supplementary Table 1) and 79.6 Gb and 100.8 Gb of Illumina paired-end/mate-paired short reads for AM and SA (Supplementary Fig. 6; Supplementary Tables 2-3), respectively. We also generated 18-23 Gb data of RNA sequences (library insert size of 300 bp) for each species (Supplementary Table 8).

The RA and BG genomes were sequenced using the same strategy [1] (Li et al. unpublished data). For the *de novo* sequencing of the *S. caseolaris* genome, 72.3 Gb of whole-genome Illumina short-reads and 54 Gb of transcriptome data were generated. The information of data collection for other species is given in Table 1.

De novo genome assembly

We *de novo* assembled the genomes based on the SMRT long-reads using four pieces of software: falcon (https://github.com/PacificBiosciences/FALCON/), DBG2OLC [62], smartdenovo (https://github.com/ruanjue/smartdenovo), and wtdbg (https://github.com/ruanjue/wtdbg). We then chose the best result, generated by smartdenovo. Genome polishing was performed using Quiver [63] to further improve site-specific consensus accuracy. Clean reads from four Illumina short-read libraries (200 bp, 300 bp, 400 bp and 600 bp) were mapped to the genome assembly using BWA [64]. We next used samtools [65] and in-house scripts to call and correct SNPs and indels. Finally, we generated scaffolds and performed gap-filling with SSPACE 3.0 [66] with default parameter values using 10 Kb mate-pair sequencing data.

To improve the genome assembly of AM and BG, three-dimensional proximity information was obtained by high-throughput chromosome conformation capture sequencing (Hi-C) [67]. We used Juicer [68] and HiC-Pro pipeline for Hi-C data processing [69].

The total size of the assembled SA genome is 207.2 Mb, or 73% of the size estimated by flow cytometry (Supplementary Table 4 and Supplementary Fig. 7). The assembled SA genome consists of 108 scaffolds with N50 at 5.52 Mb (Supplementary Table 5). The 13 (or 42) longest scaffolds cover 50% (or 90%) of the genome, respectively. The assembled AM genome at 458.3 Mb is more than twice the size of SA and is also close (~90%) to the size estimated by flow cytometry (see Supplementary Tables 1, 4 and 5, Supplementary Fig. 7 for details). The assembly contains 421 scaffolds, with the 60 largest ones cover half of the genome. The longest and N50 are 8.9 Mb and 2.3 Mb, respectively. To improve the assembly of this larger genome, we utilize the 3D proximity information obtained by chromosome conformation capture sequencing (Hi-C) [67]. The final chromosome-scale assembly consists of 32 scaffolds (5.8 - 22.7 Mb) with N50 at 15.1 Mb (Supplementary Tables 5). The BG genome is also well assembled by SMRT and Hi-C, which N50 is 14.7 Mb in 18 chromosome-level scaffolds (Li et al. unpublished data). The assembled RA genome consists of 142 scaffolds with N50 at 5.4 Mb [1].

Assessment of genome completeness

To assess the quality of genome assembly and gene annotation, we calculated the matched fractions of the transcripts, core eukaryotic genes and randomly selected genes, which were standard methods [4,92] and widely used in genome completeness assessment. These assemblies show high accuracy and completeness and compare favorably with published plant genomes in assembly length and quality (Supplementary Note and Supplementary Tables 6 and 7).

We first used BLAT (v.34x12) [93] to align assembled transcripts (241,439 and 226,814 for AM and SA, respectively) to the scaffold of each species. We find that 99.91% of AM and 99.91% of SA transcripts are covered (Supplementary Table 6). Thus, most of the expressed genes have been sequenced and assembled. We then applied *BUSCO* (v3.1.0) to assess the completeness of the assemblies. Of the 2121 benchmarking universal single-copy orthologs, 2020 (95.2%) were found in AM and 2041 (96.2%) in SA (Supplementary Table 6).

We also aligned 96 AM and 71 SA randomly selected gene sequences from our previous work to our assembled genomes using *BLAT* (v. 34x12). We recovered 96 and 69 loci uniquely in the respective genomes (Supplementary Table 6).

The results indicate high continuity, completeness, and accuracy of the two mangrove assemblies, making them suitable and reliable for various genome-based downstream analyses.

We also compared various assembly statistics to the existing plant genomes. The comparison yielded comparable or better results for our mangrove assemblies. Scaffold N50 (a main indicator of general length of assembly), for example, was up to 5.52 Mb in *S. alba*, higher than N50 values of

many other plant scaffolds and revealing good continuity of the *S. alba* assembly (Supplementary Table 7).

Repeat sequences and gene prediction

The repeat sequences were masked throughout the genome using RepeatMasker [71] and the RepBase library [72]. Working with the repeat-masked genomes, we combined homologous protein alignment, *ab initio* gene prediction, and transcriptome data to generate gene predictions. We used exonerate (version 1.1.1) [73] for homolog-based prediction through alignment of homologous proteins from six published genomes to our repeat-masked genomes. The six published genomes are *Oryza sativa*, *Mimulus guttatus*, *Sesamum indicum*, *Populus trichocarpa,* and *Eucalyptus grandis* (downloaded from Phytozome [http://www.phytozome.net] and Sinbase [http://ocri-genomics.org/Sinbase]). *Augustus* (version 3.2.2) [75] and *GeneMark-ET* (version 4.29) [76] were used for *ab initio* gene prediction.

Predicted genes were then verified using RNA-seq data. Assembled transcripts were mapped to genomes using Tophat (version v2.1.1) [77], and cufflinks (version 2.2.1) [78] was used to identify spliced transcripts. Finally, *EVidenceModeler* (EVM) [79] was used to integrate all gene models predicted from the above approaches into a weighted and non-redundant consensus gene structure set.

As a result, 35,168 AM and 31,886 SA protein-coding genes were predicted. The average gene lengths are 3,566 bp and 2,645 bp, and the average CDS lengths are 1,167 bp and 1,205 bp, respectively (Supplementary Table 9). The scaffold length, mRNA length, CDS length, exon length, exon number per gene, and intron length distributions are shown in Supplementary Fig. 8.

Gene function annotation

Functions of genes were annotated by searching against a series of protein databases, including SwissProt, TrEMBL [80], and the NCBI non-redundant (NR) protein database using *BLAST* (v2.2.6). Protein domains were annotated using InterProScan [94]. Gene ontology (GO) classification was performed by aligning genes to the Pfam database using *HMMER2GO* (https://github.com/sestaton/HMMER2GO). The annotation of Kyoto Encyclopedia of Genes and Genomes (KEGG) metabolic pathways was conducted by aligning to the KEGG database.

As a result, 32,817 AM and 30,360 SA genes were annotated, accounting for 93.31% and 95.21% of the gene sets respectively; 22,316 AM and 20,227 SA genes have KO (KEGG Orthology) information, while GO terms were assigned to 20,627 AM and 18,590 SA loci (Supplementary Fig. 9; Supplementary Table 10).

Transcription factor annotation

The program *iTAK* (v1.2; http://bioinfo.bti.cornell.edu/cgi-bin/itak/index.cgi) was used to identify and classify transcription factors (TFs) in the two mangrove genomes. 2,426 AM and 2,254 SA TFs belonging to 58 transcription families were identified. The most abundant transcription factor family for are MYB in AM and AP2-EREBP in SA (Supplementary Table 11).

Synteny construction

Syntenic blocks within each species were identified using McscanX [85] based on BLASTp self-alignments (e-value threshold of 1×10^{-5}). Each collinear block included at least five pairs of syntenic genes. Syntenic block visualizations produced by *Circos* (v0.65) [86] are shown in Supplementary Fig. 11.

835 AM and 706 SA syntenic blocks were identified. These blocks cover 79.52% of the AM and 90.90% of the SA assembly, and accounted for 47.93% and 49.83% of genes. These syntenic blocks cover high proportions of the genome and provide strong evidence for whole genome duplication (WGD). For example, in kiwifruit and potato genomes, WGD events were identified with only 46% and 25.74% of the genome sequences or genes covered by syntenic blocks [95,96].

Analysis of transcription profiles

To explore gene expression patterns in response to salt stress, we sequenced a series of transcriptomes AM and SA under salt treatments. Seedlings were collected from Hainan Island and cultivated with 1/2 Hoagland's nutrient solution [97] on clean sand for at least seven days. The seedlings were then divided into three groups and incubated under different NaCl concentrations (0 mM, 250 mM and 500 mM NaCl in 1/2 Hoagland's nutrient solution) for seven days. The total RNA of healthy young leaves and roots from each treatment (including two individuals each) were extracted using the Plant RNA Kit (OMEGA) and sequenced with the HiSeq 2000 platform. Two independent replicated biological samples were examined.

The short reads from each sample were aligned to its reference genome using *TopHat* [77] and assembled using *Cufflinks* [78]. Two programs, *Cuffmerge* and *Cuffdiff* from the *Cufflinks* package, were used to compare expression profiles among conditions. Benjamini-Hochberg correction [98] for multiple testing was used (q-value < 0.05) in the analysis. Genes with significantly different expression levels and a fold change greater than two in both treatments were treated as differentially expressed genes (DEGs). DEGs were then mapped to KEGG pathways. Pathways with more than two differentially expressed genes were subjected to an enrichment test. Fisher's exact test with Benjamini–Hochberg correction was used to test for statistical significance.

Between 357 and 2,642 genes were identified as DEGs in roots or leaves of the two species (Supplementary Figs. 22-23), with between 10 and 36 pathways significantly enriched in differential expression. Particularly, we found that pathways related to flavonoid biosynthesis were enriched in leaves of both species when increasing salt concentration from 250 mM to 500 mM (Supplementary Table 28). Flavonoids play important roles in plant stress response. Stresses such as high salinity induce reactive oxygen species (ROS), which can alter normal cellular metabolism through oxidative damage to cellular components. Flavonoids help scavenge ROS [99]. It has been previously reported that flavonoid and total phenolic compound levels increase under salt treatment [100]. Other pathways, such as "Photosynthesis" and "Plant hormone signal transduction" were also enriched in salt-induced or repressed genes.

2. Divergence time and whole-genome duplication (WGD)

Estimation of species divergence time

To explored the origin and divergence time of the three mangrove lineages—*Avicennia*, Rhizophoreae and *Sonneratia*, we reconstructed phylogenetic trees and estimated the species divergence times in each lineage.

The data used in this analysis include five *de novo* genome assemblies (AM, RA, BG, SA and SC), one whole-genome resequencing data set (*A. marina* var. *australasica*), 10 transcriptomes of mangrove or non-mangrove relatives and five published genomes (Supplementary Table 14). The transcriptomes were first assembled using *Trinity* and the coding region sequences (CDS) were obtained by aligning to closely related mangrove reference genomes using *BLASTx* with an e-value threshold of 1×10^{-5}. The CDS of *A. marina* var. *australasica* were obtained by mapping the genome sequences to the *A. marina de novo* assembly. Protein sequences were translated from CDSs using a BioPerl script.

Gene clustering was performed using *OrthoMCL* [83] in each species group (Supplementary Fig. 12), yielding 26,384, 23,356, and 35,206 gene clusters. We picked gene families containing only one member of each species (single-copy ortholog groups) for downstream analyses. The putative orthologs were aligned using a combination of *PAL2NAL* [101] and *MUSCLE* [102]. Short (< 150 bp) or large-K_a (> 0.5 between *A. marina* & *S. indicum*, *R. apiculata* & *P. trichocarpa*, or *S. alba* & *E. grandis*) orthologs were removed. The filtered sets contain 1,421, 1,488, and 617 single-copy orthologous groups.

PhyML [84] was used to reconstruct phylogenetic trees within each group with 1000 bootstrap replicates. All nodes were 100% supported (Supplementary Fig. 16). The program *MCMCTREE* from the *PAML* 4.8 package [33] was used to calculate divergence times with the parameters "seq like (usedata = 1)," "HKY85+gamma (model = 4, alpha = 0.5)" and "independent rates (clock = 2)." *MCMCTREE* from the *PAML* package is one of the most popular approaches that implements a Bayesian phylogenetic framework to estimate divergence time. Bayesian methods are popular because they can readily incorporate complex models of molecular evolution [103]. Many recent studies used *MCMCTREE*, especially for genome papers, such as Yim et al. [104], Frantz et al. [105], and Ma et al. [4]. We set the parameters as suggested in the *PAML* Manual and we ran each analysis twice to make sure the results converged.

We used the following time calibrations in our analyses:

Avicennia group: The root constraint for *Mimulus guttatus* and the common ancestor of other species was set to 70-75 million years (Myr) ago [106] (Supplementary Fig. 13).

Rhizophoreae group: The time constraints are the same as in Xu et al. [1]. The Malpighiales root node, the common ancestor of Rhizophoraceae, Euphorbiaceae (*Ri. cimmunis*), and Salicaceae (*P.*

trichocarpa), was placed at 105-120 Myr before present [107,108] (Supplementary Fig. 14). An additional constraint (the red box 2 in Supplementary Fig. 14c) was between the *Rhizophora* lineage and the common ancestor of *Ka. obovata* and *Ce. tagal*, which was set earlier than 38 Myr ago since the earliest convincing fossils of *Rhizophora* have been dated to the late Eocene (33.9-38 Mya) [109,110].

Sonneratia group: The divergence time of the node between *E. grandis* and the other species was set to 100-110 Myr ago, equal to the divergence time between Myrtaceae and Lythraceae [111,112] (Supplementary Fig. 15). The earliest confirmed fossil of *Lagerstroemia* is a leaf impression dating to the late Paleocene/early Eocene. Therefore, we set another time constraint between *Lagerstroemia speciosa* and *Duabanga grandiflora* as 47.8-59.2 Myr ago [90,113].

As a result, the origin of the *Avicennia* mangroves is placed between 53 and 38 Myr ago (Supplementary Fig. 13). The origin of *Acanthus ilicifolius*, occurred during the last 16.6 Myr and is the only possible example of recent origin for mangroves in our collection. Rhizophoreae has the largest number of genera (*Rhizophora*, *Bruguiera*, *Ceriops*, and *Kandelia*), and its origin can be dated between U3 (at 54.1 Myr ago) and L3 (at 47.8-56 Myr ago) [89] (Supplementary Fig. 14). The last group includes the mangrove genus *Sonneratia* and the freshwater genus *Trapa*. Their most recent common ancestor is marked U4 in Fig. 1, with several mangrove fossils also indicated [90] along the branches. Given that the common ancestor U4 was aquatic and the *Sonneratia*-like fossil appeared early, the origin of *Sonneratia* is likely close to the U4 time at 42.5 Myr ago. L4 is placed at the lower boundary of the dating of the fossils suspected to be the ancestral *Sonneratia* (Supplementary Fig. 15). Divergence times estimated using by varying datasets and methods were consistent with those presented above (Supplementary Tables 15-20, Supplementary Fig. 16).

<u>Comparisons of divergence times estimated using different datasets / methods</u>

To assess the robustness of the results, we calculated divergence times using several datasets and methods. We first compared the divergence time calculated with two sets of data: all three nucleotides of codons, and only the 1st and 2nd positions. The results positions are consistent (Supplementary Tables 15-17).

We then used *r8s* [114] to date the divergence time. Using *r8s*, the phylogeny with branch length and nodes constraints are necessary. Therefore, we built phylogenetic trees with branch lengths using two different nucleotide substitution models: HKY85+G and GTR+I+G. In r8s software, there are three divergence time calculation methods: LF (Langley-Fitch), PL (Penalized likelihood), and NPRS (nonparametric rate smoothing) and three algorithms: TN, Powell, and Quewt for these methods. As per recommendations in the user's manual, we conducted our calculation using the TN algorithm for the LF and PL method, and the Powell algorithm for the NPRS method. The dataset including all orthologs described in the previous section was used in the *r8s* analysis.

The comparisons of the two approaches show that the outputs generally converge well (Supplementary Tables 18-20). The largest discrepancy is the dating of U1 and U2 in Fig. 1, which are 66.5 and 53.1 Myr based on *MCMCTREE* and 47 and 39 Myr from *r8s* (Node 2 and 3 in

Supplementary Fig. 16). One of the possible explanations is that *r8s* only used phylogenetic branch lengths and time constrains of some nodes to estimate time. The node time is influenced mostly by the branch length and might lose important information provided by sequence data (*r8s*, version 1.70 user's manual, page 22). In addition, *MCMCTREE* provided 95% confidence intervals of each node unlike *r8s*. Hence, the results from the *MCMCTREE* analyses are shown in the main text.

<u>Dating whole-genome duplication (WGD) events</u>

In addition to the circular diagrams provided in Supplementary Fig. 17 to illustrate intra-species synteny, we tested other important aspects of WGD. A classical approach of WGD detection is through the distribution of synonymous substitution rates (Ks), an indication of the relative duplication age. This method has been widely used in studies of the same type [115,116]. Based on the genome-wide syntenic blocks identified, we calculated the Ks values for all paralogous gene pairs. A large peak was observed in each mangrove genome, indicating WGD events (Supplementary Figs. 13-15).

To date the mangrove WGDs, we first compared Ks distributions of syntenic gene pairs within mangrove genomes to Ks calculated between orthologous genes from mangrove-relative pairs. The comparison was used to infer the phylogenetic branch where the WGD events occurred. Then, we approximately dated the WGD events on branches using the following steps. First, we calculated the branch length (nucleotide substitution) distribution of syntenic gene pairs in each genome and found the distribution peaks (L_peak). We then calculated branch lengths between the WGD event and the closest node. Therefore, the time t_1 between the WGD and the node is ((L_peak - L_node)/u). L_node is the branch length between the node and the present. u is the average mutation rate on this branch and was estimated from previous analyses of divergence time. We estimated WGD age by adding t_1 and the divergence time of the closest node t_2.

The Ks distribution of *A. marina* syntenic genes and that of other orthologs showed that the WGD event occurred near their divergence node within a narrow time range. The peak of branch length distribution of syntenic genes for *A. marina* and *S. indicum* is 0.29 and 0.25, and the WGD event happened at about 68 and 69 Myr ago, respectively (Supplementary Fig. 13).

The peak of Ks among *R. apiculata* syntenic genes is smaller than that of orthologs between *R. apiculata* and *P. trichocarpa,* but exceeds other Ks pairs, indicating the whole-genome duplication event occurred before the divergence of the common ancestor of *Carallia brachiata* and *Pellacalyx yunnanensis* and other species. The peak of branch length distribution of syntenic *R. apiculata* genes is 0.17, yielding estimated WGD time of about 67 Myr ago (Supplementary Fig. 14).

The Ks peaks for *S. alba* and *S. caseolaris* syntenic genes from the *Sonneratia* group is smaller than that for *S. alba* and *E. grandis* orthologs but larger than other Ks pairs, indicating the whole-genome duplication event occurred before the divergence of the common ancestor of *L. speciosa* and *D. grandiflora* and other species. The peaks of syntenic gene branch length distributions for *S. alba* and *S. caseolaris* are all 0.21. Hence, we calculated the age of WGD event occurred approximately 68 Myr ago (Supplementary Fig. 15).

There are reasons to believe that our methods and results are reliable. First, we estimated the time of a relatively recent WGD in *Sesamum indicum* using the same methods as in our study. As a result, this WGD was dated to about 69 Myr ago, corresponded almost exactly with the previous research, which dated the WGD to around 71 Myr [27]. Moreover, our dating of the WGDs in three lineages placed these events at 67-69 Myr ago, coinciding with the Cretaceous-Tertiary boundary and similar to that observed in many other angiosperms [117].

3. Gene family analyses

Orthologous and paralogous groups were inferred using *OrthoMCL* [83]. In addition to the three mangrove species sequenced in this study (AM, RA, and SA), protein-coding genes from four land species: *O. sativa*, *E. grandis*, *P. trichocarpa* (http://www.phytozome.net), and *S. indicum* (http://ocri-genomics.org/Sinbase) were used for clustering. For genes with alternative splicing, the longest transcripts were selected. The proteins from these seven species were combined to perform an all-vs.-all comparison using *BLASTp* with an e-value cutoff of 1×10^{-10}. The results were fed into a stand-alone *OrthoMCL* program with the default MCL inflation parameter set to 2.0.

35,168 protein-coding *A. marina* genes were classified into 13,850 families, with 544 clusters comprising 1,710 genes specific to that species. 26,640 protein-coding *R. apiculata* genes were classified into 13,151 families, with 469 clusters comprising 1,360 species-specific genes. 31,886 *S. alba* genes were classified into 13,064 families, with 782 clusters comprising 2,045 species-specific genes (Supplementary Table 13 and Supplementary Fig. 12). 6,904 families are shared by all seven species and 9,507 families are shared by AM, RA, and SA.

After gene family clustering, CAFE [87] was used to analyze gene family expansion and contraction in the context of the phylogeny of the seven species. The phylogenetic tree topology and branch lengths were considered to infer the significance of change in gene family size for each branch. The patterns in the three mangrove species were compared to other genomes. In mangroves, 95/192, 23/303 and 29/288 gene families have expanded/contracted while the trend is reversed in the non-mangrove relatives (165/135, 266/40 and 284/36). The differences are highly significant by the G-test and, by a stringent likelihood model implemented in CAFE ($P < 0.05$).

In total, 58 gene families have contracted in all three lineages - AM, RA and SA (Supplementary Table 26). Gene families pertaining to disease resistance represent the most conspicuous contractions (9 out of 58) including the putative receptor serine/threonine kinase genes (PR5K). In addition, 51 disease-related gene families have contracted in at least one of the mangrove genera. They include the TIR-NBS-LRR class and the NB-ARC domain-containing proteins, both of which belong in the largest class of plant disease resistance genes (Supplementary Table 27).

The diversity of pathogens in the intertidal habitats has been reported to be low in some taxa (e.g., fungi) [118] but high in others [119]. The shedding of disease resistance genes could be due to the diminished needs to defend against certain pathogens and/or the increasing demands to cope with other

aspects of a novel physical environment. For pathogen resistance, mangroves appear to rely on secondary compounds [120], among which tannins are particularly common in the three genera [121]. The high content of tannins has earned mangroves the nickname of “red trees”.

4. Amino acid composition analyses

We first compared amino acid (AA) compositions of three mangroves with 54 inland dicotyledon plants (Fig. 3a). We then focused on the comparison of AA usage of AM, RA, and SA with their inland relatives (*S. indicum*, *P. trichocarpa*, and *E. grandis*, respectively). We found that 13 of 20 AAs’ frequencies increased or decreased in the same way in all three mangrove genomes, and 11 of which changed significantly (P value < 0.01, chi-square test). Other mangroves in the three genera show a similar pattern (Supplementary Fig. 18).

Amino acid composition is also influenced by genomic GC content as presented in the main text and tabulated in Supplementary Table 24. To conform that the high GC content of coding regions is indeed correlated with amino acid usage rather than codon usage bias, we removed four-fold degenerate sites and calculated the “adjusted” GC content presented in Supplementary Table 25. The adjusted GC content of mangrove species is still higher than in their inland relatives. We also compared the GC content between mangroves and their inland relatives at intron regions. And we found the intron regions of mangroves didn’t have the same trends of GC content increase (Supplementary Table 24). These results indicate that the increased GC content observed in mangrove species is attributable to skewed amino acid usage rather than codon usage bias.

In the main text, we suggest that physiological conditions in mangrove cytosol likely differ from terrestrial plants at least in some tissues especially in the period after salt concentration changes. Below is additional information from the literature on this issue. While some (but not all) studies have reported that mangroves can keep the cytosol salt concentration at a low level when the concentration reaches an equilibrium, the issue is how fast this equilibrium is approached. It is known that mangroves and other halophytes compartment salt into the vacuole to avoid osmotic imbalance and Na^+ toxicity to the cell. Tonoplast-located ion exchangers transfer Na^+ and Cl^- from cytosol to the vacuole and the salt concentration in this organelle is much higher than that in cytosol.

Previous studies have found that, under salt treatment, the concentration of Na^+ and Cl^- in both the vacuole and the cytosol increases. Li et al. [44] found the Na^+ and Cl^- concentration in the cytoplasm increased after salt treatment in two mangrove species, *K. candel* and *B. gymnorhiza*. Kura-Hotta et al. [43] found an increase of Na^+ and Cl^- in both the cytoplasm and vacuole of *B. gymnorhiza* after 150 mM NaCl treatment. Although the concentration of Na^+ and Cl^- returned to a low level after a rapid increase, this took time. The Na^+ levels need about two weeks to recover, and Cl^- takes even longer. In the natural environment, most salt is stored in the vacuole but the salt concentration in the cytosol of mangroves would still fluctuate much more than in glycophyte. The high concentration of salt in the cytosol is harmful to the cell, especially to protein activities. Hence, amino acid composition may change as a response to this selection pressure.

Supplementary Figures

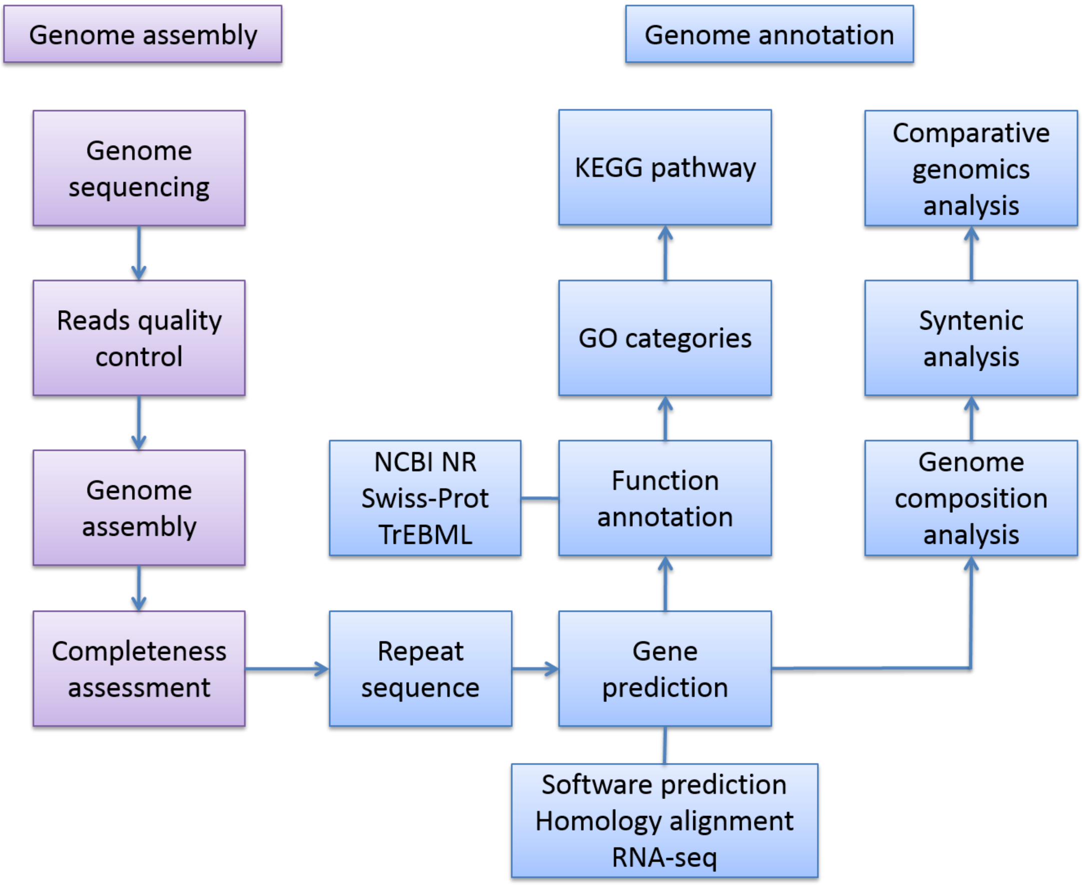

Supplementary Figure 1. Workflow of genome assembly and annotation.

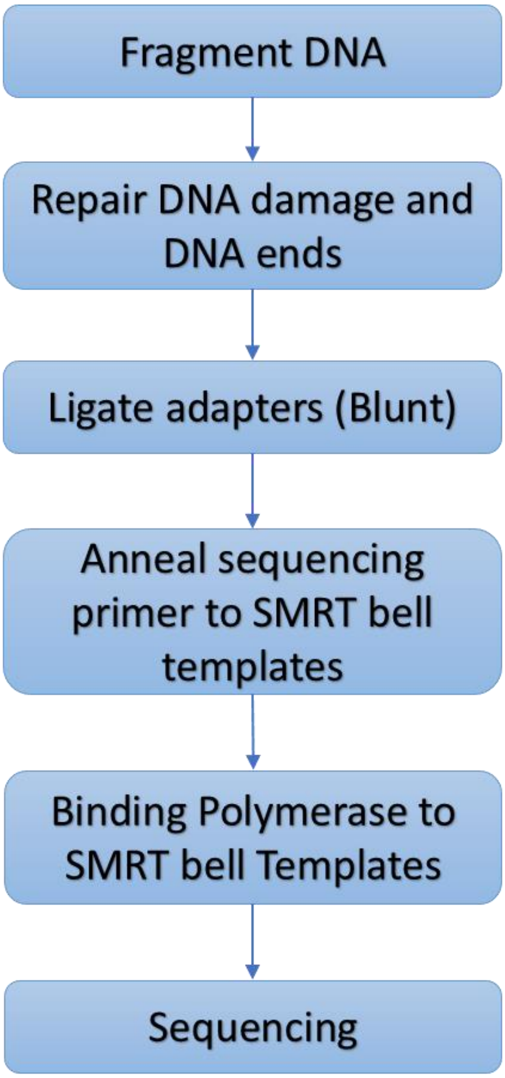

Supplementary Figure 2. Workflow of PacBio SMRT library preparation.

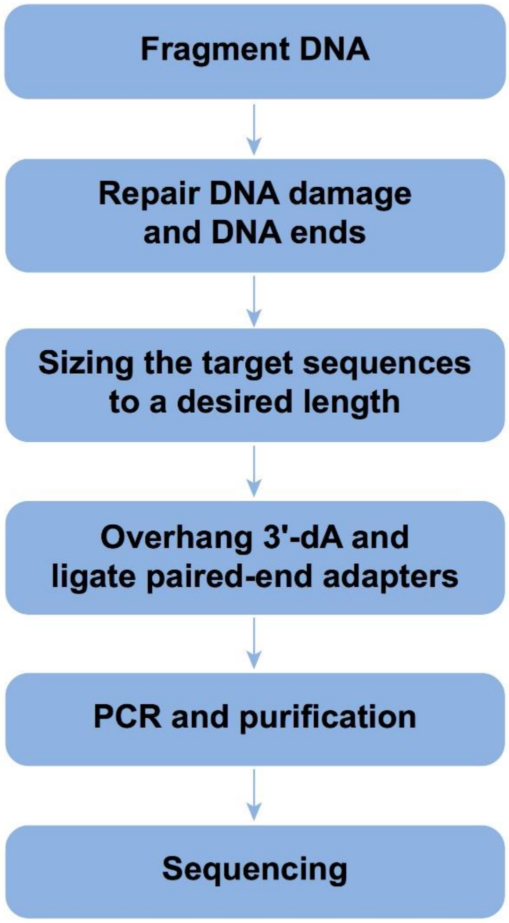

Supplementary Figure 3. Workflow of Illumina short reads library preparation.

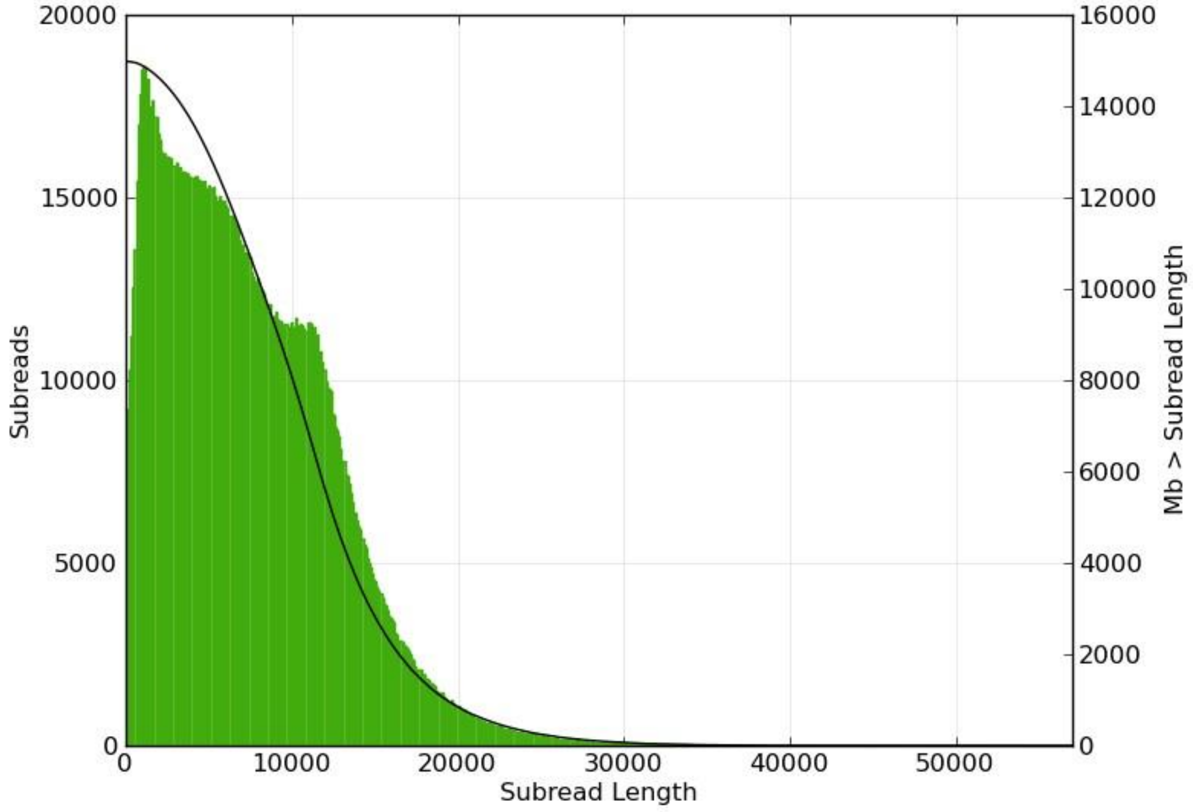

Supplementary Figure 4. Histogram of PacBio SMRT P6-C4 subread lengths from *A. marina*. Subreads are generated when adapters located within a read are removed. The x-axis is subread length. The green histogram shows the number of reads in bins of length intervals, while the black line shows total number of subreads (Mb) with length larger than x bp.

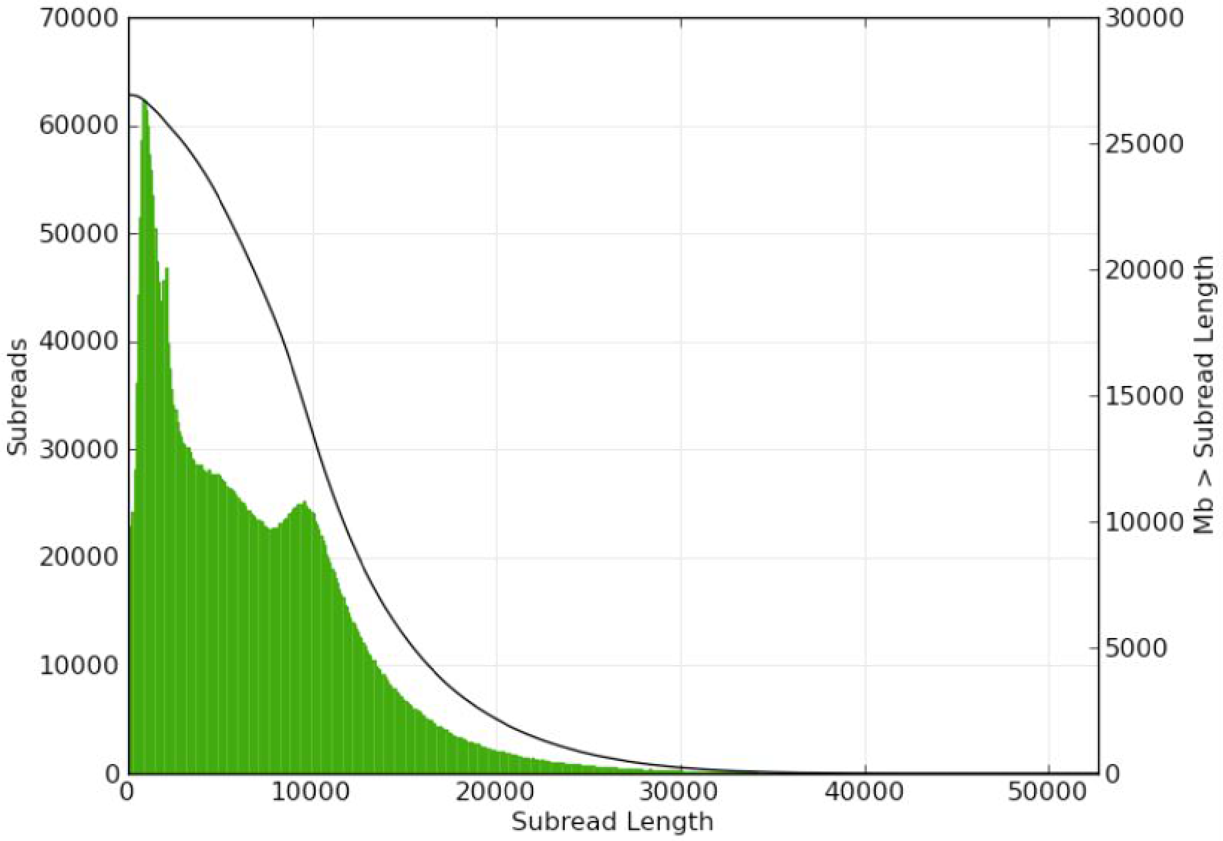

Supplementary Figure 5. Histogram of PacBio SMRT P6-C4 subread lengths from *S. alba*. Subreads are generated when adapters located within a read are removed. The x-axis is subread length. The green histogram shows the number of reads in bins of length intervals, while the black line shows total number of subreads (Mb) with length larger than x bp.

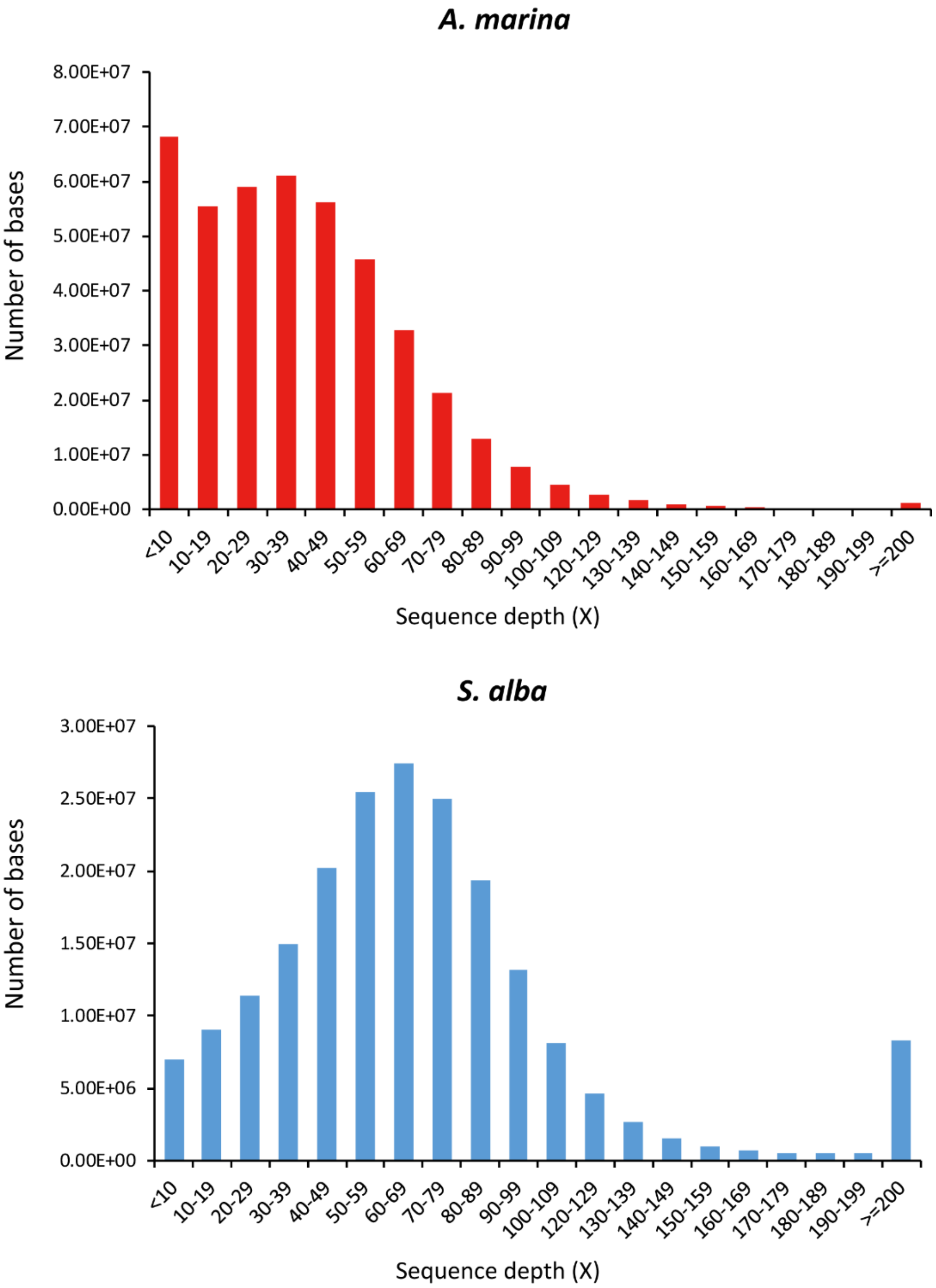

Supplementary Figure 6. Sequencing depth distribution of two mangrove genomes. Reads from small-insert-size libraries were mapped to the genomes using *Bowtie2* [122].

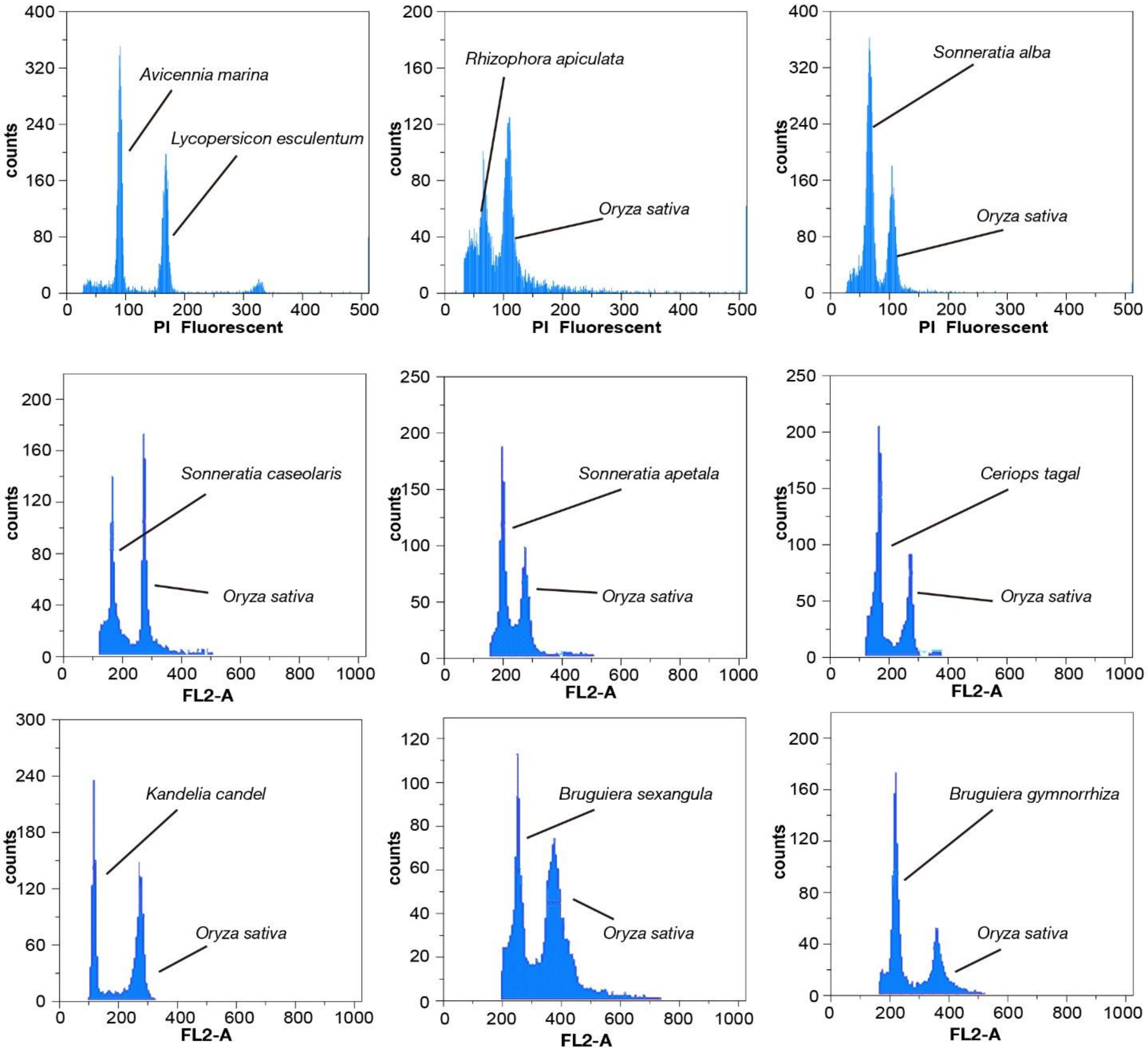

Supplementary Figure 7. Genome size estimation of mangroves species. Genome sizes were estimated by counting the number of particles in suspension using flow cytometry. Either *Oryza sativa* subsp. *japonica* cv. Nipponbare (1*C* = 442 Mb) or *Lycopersicon esculentum* cv. Stupicke polni (1*C*=958Mb) were used as internal standards. The relative positions of the two bars on the x-axis were used to estimate the genome sizes. See Supplementary Table 4.

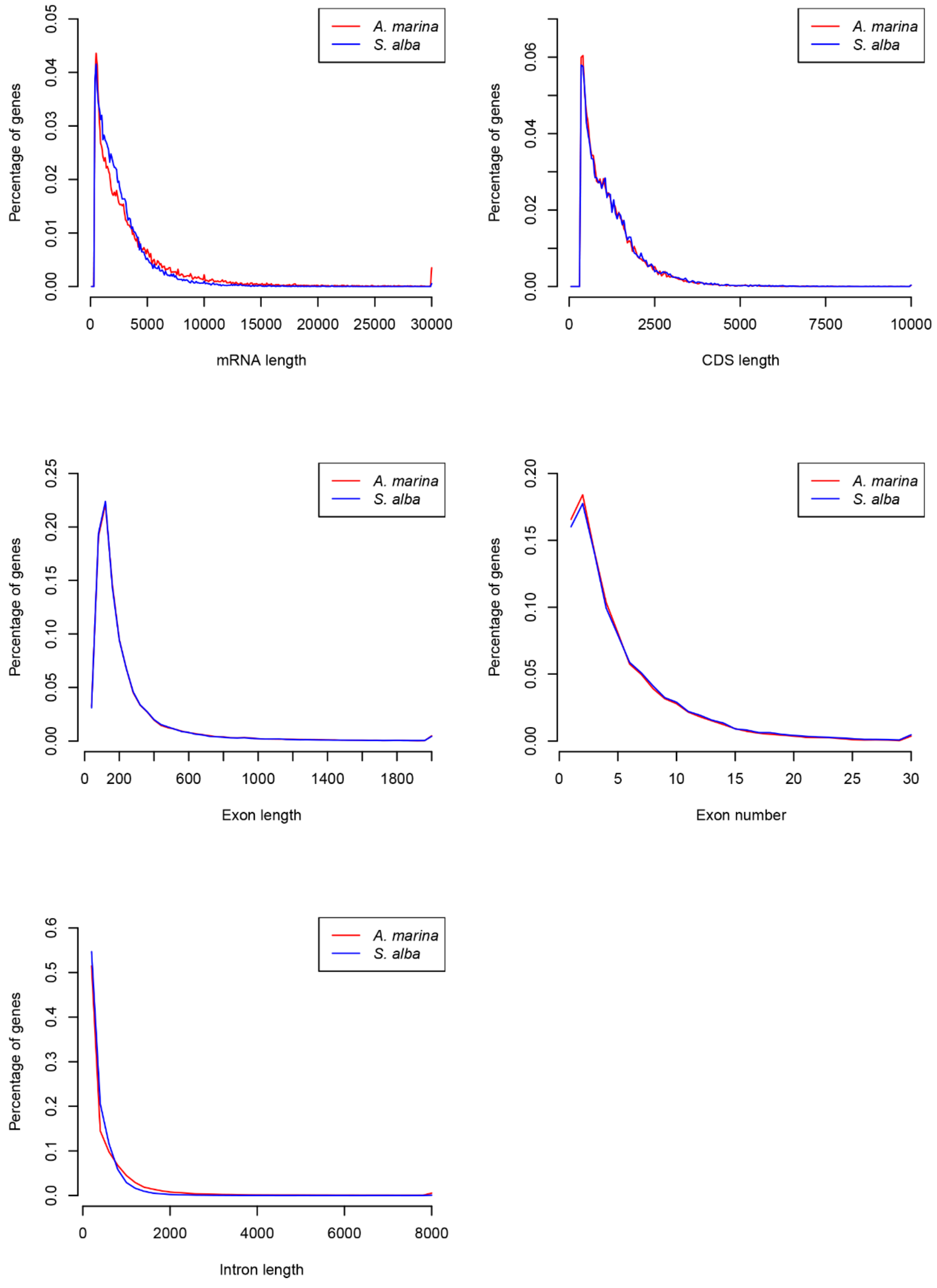

Supplementary Figure 8. Comparison of mRNA length, CDS length, exon length, exon number per gene, and intron length of *A. marina* and *S. alba*.

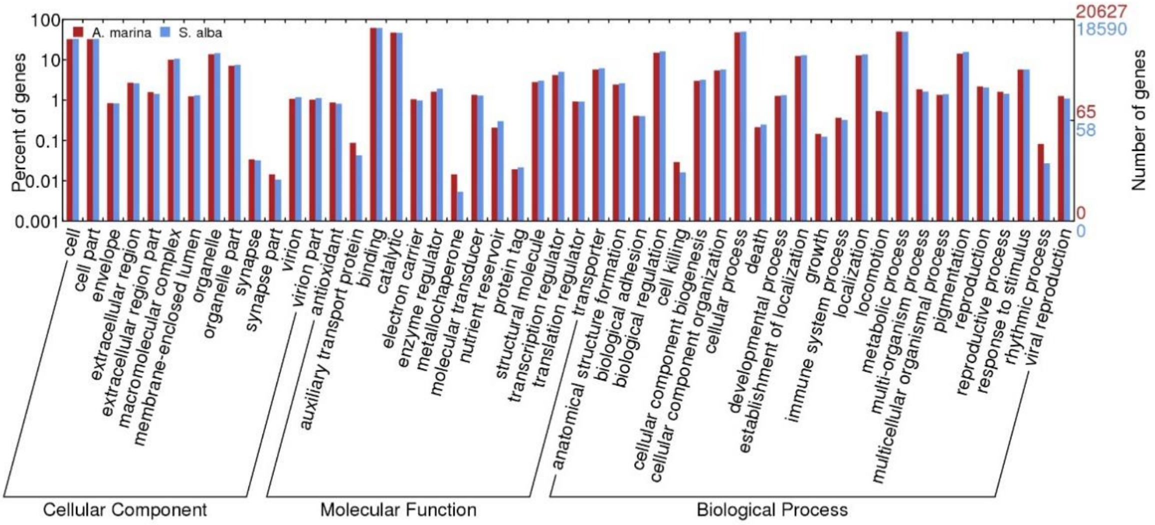

Supplementary Figure 9. GO annotation of AM and SA genes. The number of genes in each category was generated using WEGO (http://wego.genomics.org.cn/cgi-bin/wego/index.pl).

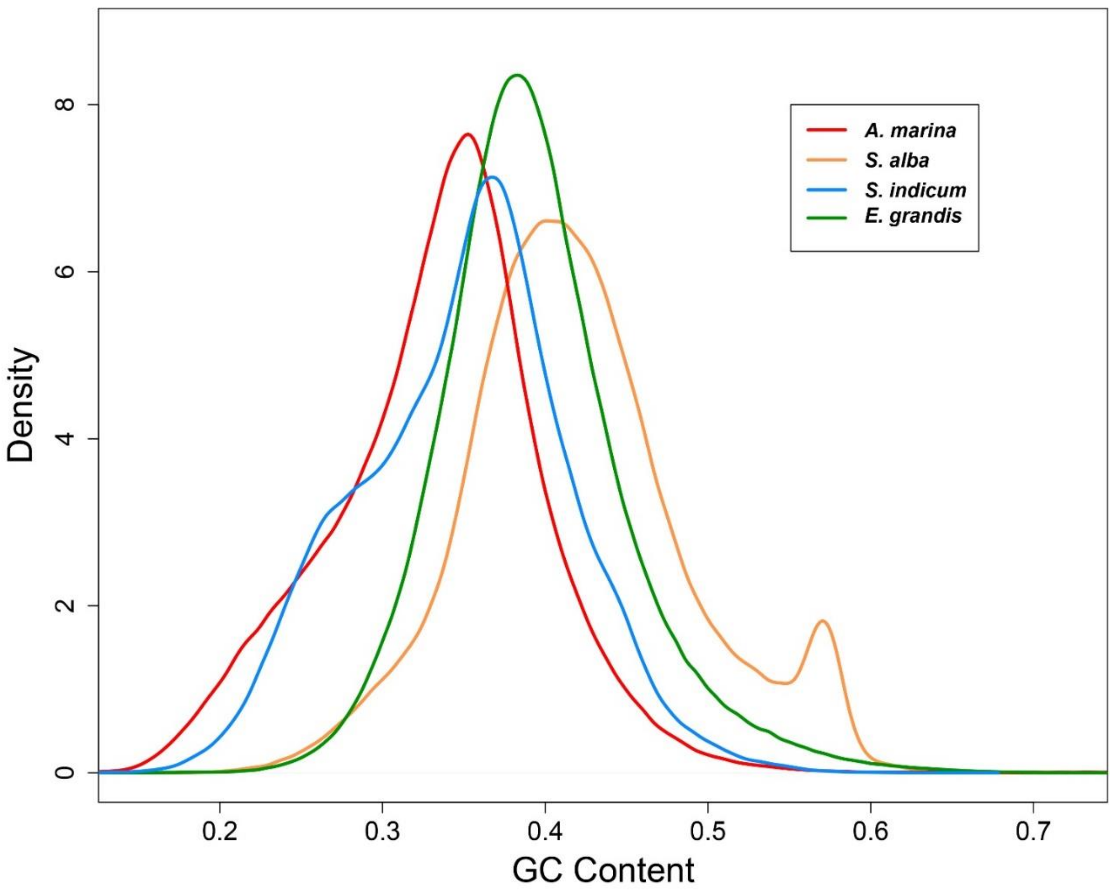

Supplementary Figure 10. GC content distribution in the genomes of AM, SA and their inland relatives. The distribution was estimated using 500 bp non-overlapping sliding windows.

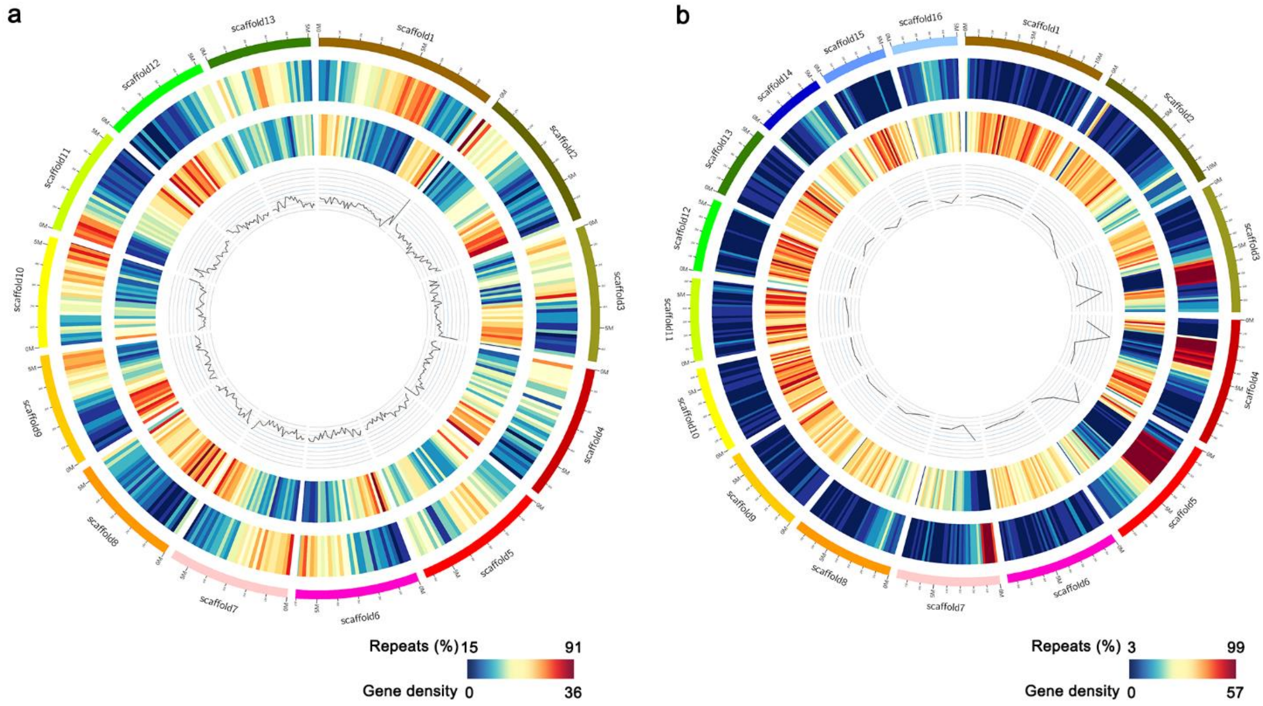

Supplementary Figure 11. AM and SA genomic features. *(a) A. marina*. *(b) S. alba*. The tracks are, from the outer to the inner ring, scaffolds (>5 Mb), percentage of repeats (15-91 % per 200 Kb for AM and 3-99 % per 200 Kb for SA,), gene density (0-36 per 200 Kb for AM and 0-57 per 200 Kb for SA), and GC content (31.05 – 40.11 % per 200 Kb for AM and 36.68 – 54.09 % per 200 Kb for SA).

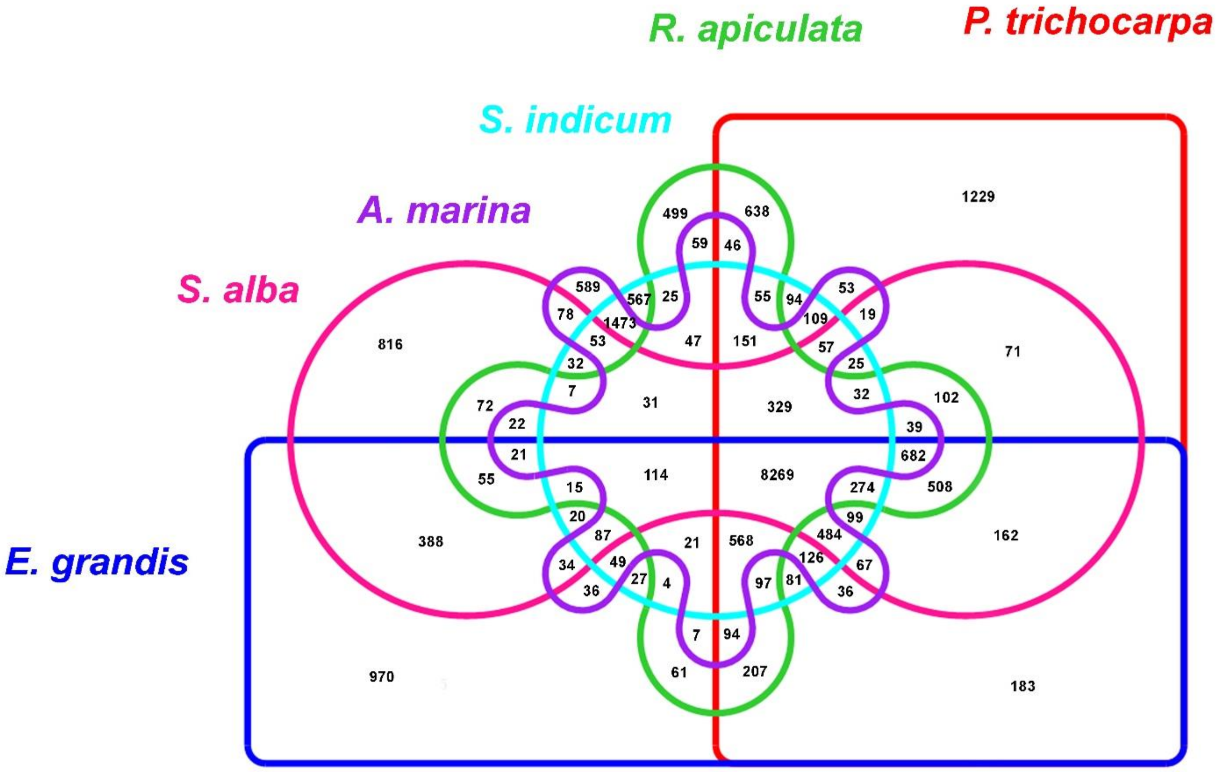

Supplementary Figure 12. Unique and shared gene families from three mangroves (*A. marina*, *R. apiculata*, and *S. alba*) and their inland relatives.

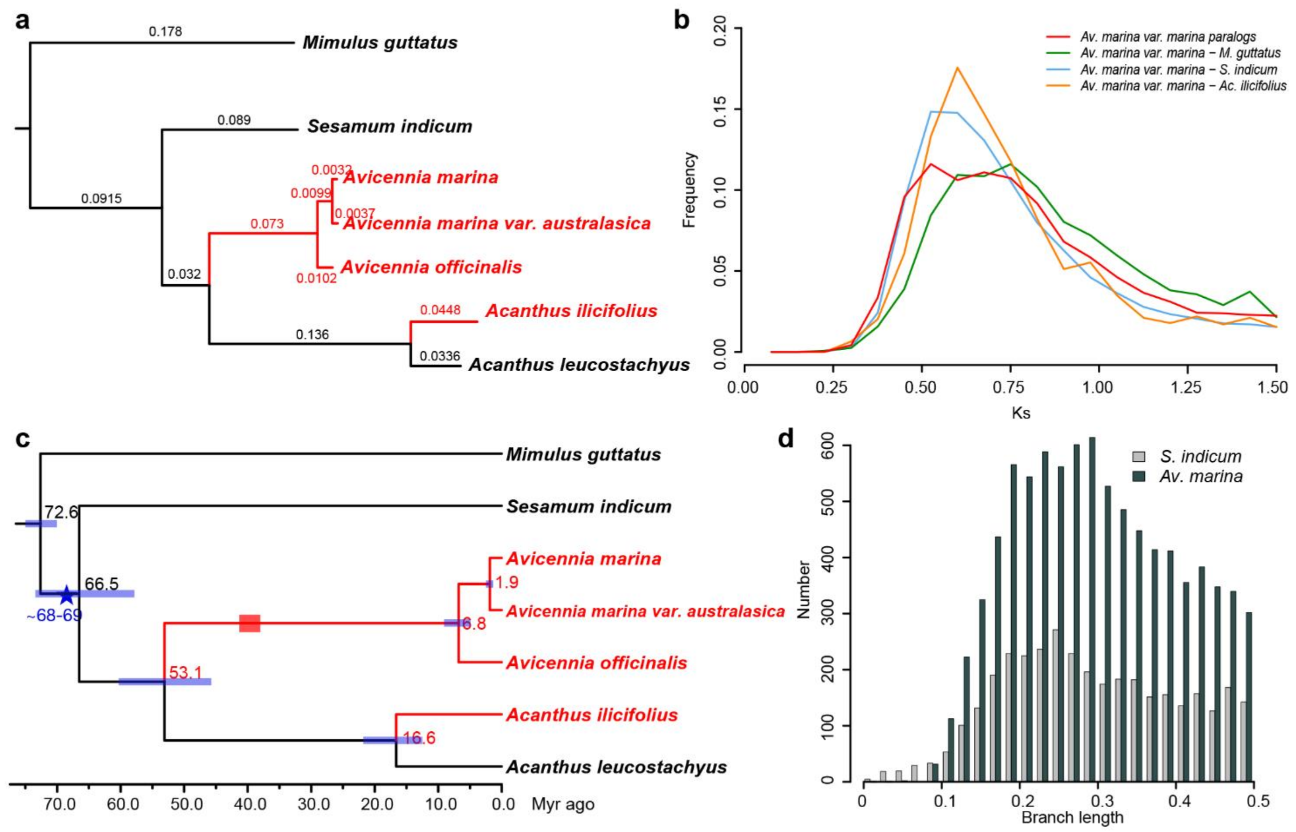

Supplementary Figure 13. Divergence time and whole-genome duplication dating of the *Avicennia* group. *(a)* Phylogenetic tree of the *Avicennia* group: the numbers above the branches represent nucleotide substitution rates from the HKY85+gamma model and 1000 bootstraps. All nodes are 100% supported. Red branches and names indicate mangrove species. *(b)* Ks distributions in the *Avicennia* group: *Av. marina* paralogs, the Ks distribution of syntenic genes from *Av. marina*; *Av. marina–M. guttatus*, the Ks distribution of orthologs between *Av. marina* and *M. guttatus*; *Av. marina–S. indicum*, the Ks distribution of orthologs between *Av. marina* and *S. indicum*; *Av. marina–Ac. ilicifolius*, the Ks distribution of orthologs between *Av. marina* and *Ac. ilicifolius*. *(c) Avicennia* group divergence time. Red branches and names represent mangrove species. The blue bars show 95% confidence intervals. The blue star and the number under it mark the whole-genome duplication event. Red rectangle marks the earliest and most confirmed fossil record from the *Avicennia* lineage, which is from Spain and dated to Middle Bartonia (38-41.3Myr ago) [34]. *(d)* The branch length (nucleotide substitution) distribution of syntenic gene pairs from *Av. marina* and *S. indicum*. The peaks are at 0.29 and 0.25, respectively.

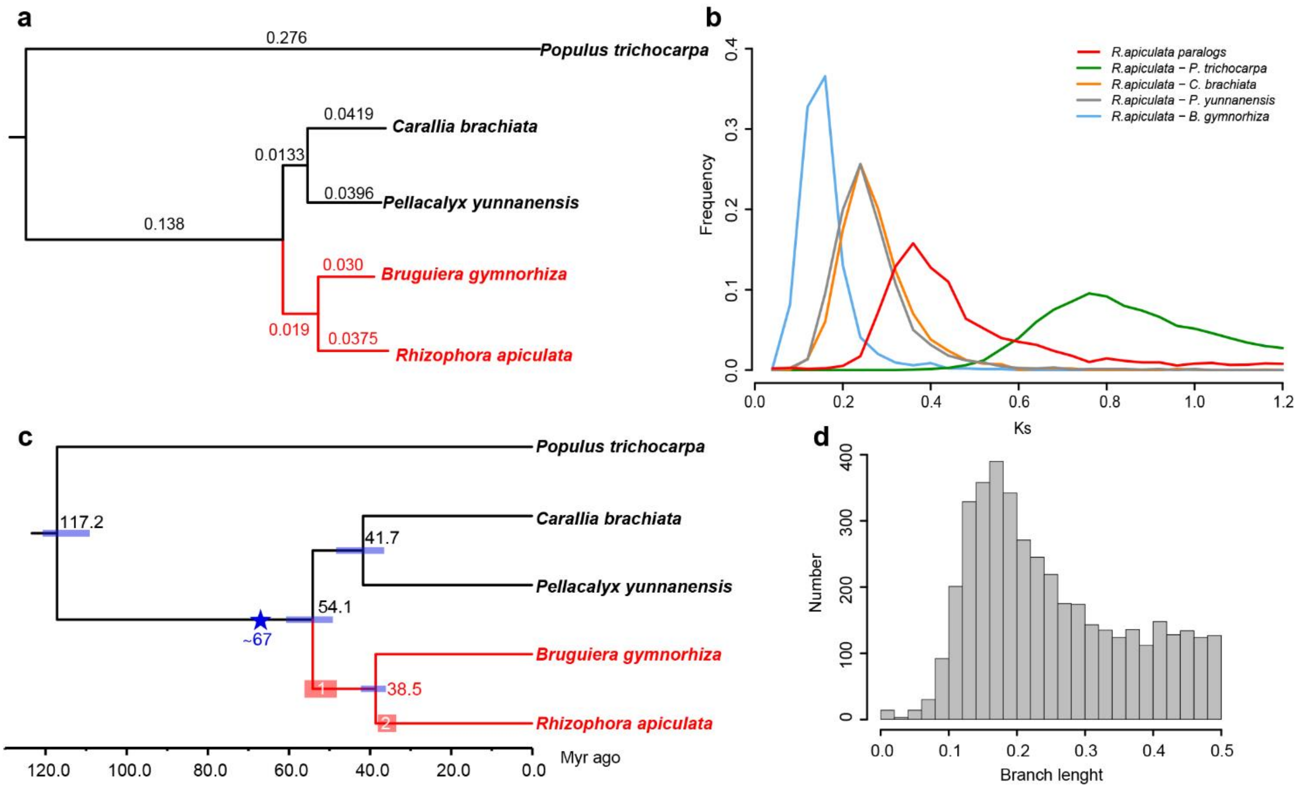

Supplementary Figure 14. Divergence time and whole-genome duplication dating of the Rhizophoreae group. *(a)* Phylogenetic tree of the Rhizophoreae group: the numbers above each branch represent the nucleotide substitution rate from the HKY85+gamma model and 1000 bootstraps. All nodes are 100% supported. Red branches and names indicate mangrove species. *(b)* Ks distribution of the Rhizophoreae group: *R. apiculata* paralogs, the Ks distribution of syntenic *R. apiculata* genes; *R. apiculata–P. trichocarpa*, the Ks distribution of orthologs between *R. apiculata* and *P. trichocarpa*; *R. apiculata–C. brachiata*, the Ks distribution of orthologs between *R. apiculata* and *C. brachiata*; *R. apiculata–P. yunnanensis*, the Ks distribution of orthologs between *R. apiculata* and *P. yunnanensis*; and *R. apiculata–B. gymnorhiza*, the Ks distribution of orthologs between *R. apiculata* and *B. gymnorhiza*. *(c)* The Rhizophoreae group divergence time. The blue bars show 95% confidence intervals. Red branches and names represent mangrove species. The blue star and the number under it mark the whole-genome duplication event. Red rectangles with numbers represent the earliest fossil records of mangrove lineages: 1) Hypocotyls resembling *Bruguiera* are known from the London Clay and are identified as *Palaeobruguiera* in early Eocene (47.8-56 Myr ago) [89]; 2) the oldest records of *Rhizophora* were dated to the upper Eocene (33.9-38 Myr ago) [109]. *(d)* The branch length distribution of syntenic gene pairs for *R. apiculata*. The peak is at 0.17.

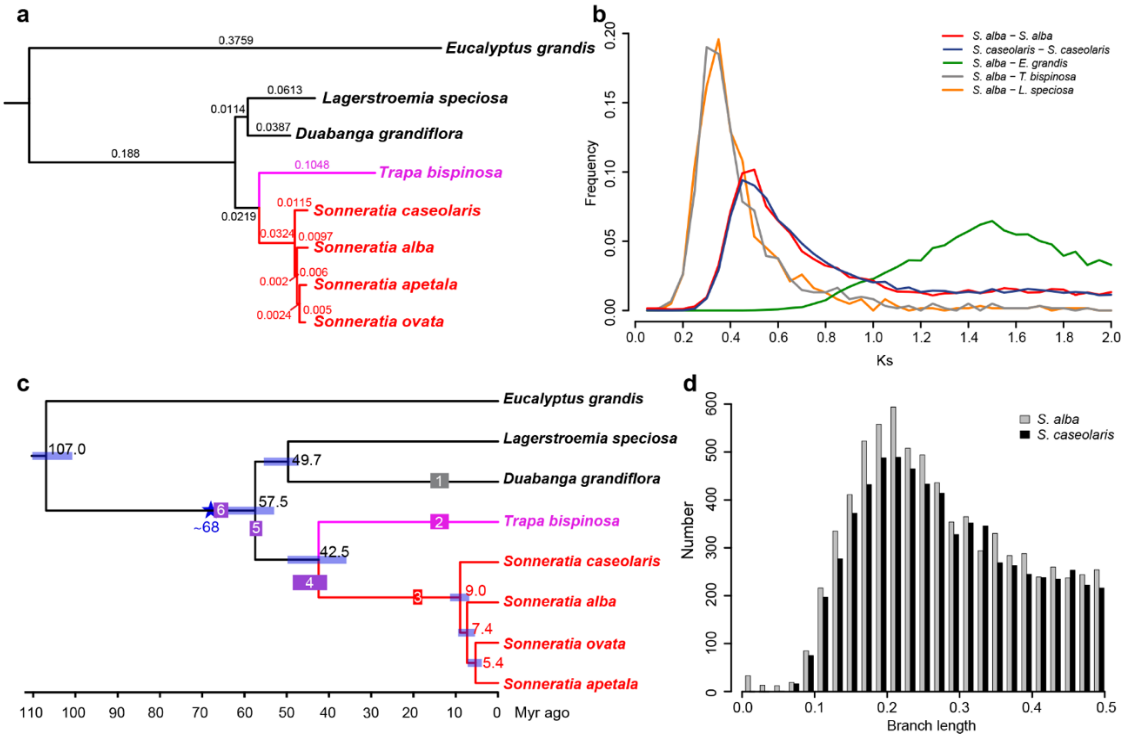

Supplementary Figure 15. Divergence time and whole-genome duplication dating of the *Sonneratia* group. *(a)* Phylogenetic tree of the *Sonneratia* group: the numbers above the branch represent the nucleotide substitution rates from the HKY85+gamma model and 1000 bootstraps. All nodes are 100% supported. Red branches and names indicate mangrove species. Magenta branches and names indicate the freshwater genus *Trapa*. *(b)* Ks distributions of *Sonneratia* group: *S. alba* paralogs, the Ks distribution of syntenic genes from *S. alba*; *S. caseolaris* paralogs, the Ks distribution of syntenic genes from *S. caseolaris*; *S. alba–E. grandis*, the Ks distribution of orthologs between *S. alba* and *E. grandis*; *S. alba–L. speciosa*, the Ks distribution of orthologs between *S. alba* and *L. speciosa*; and *S. alba–T. bispinosa*, the Ks distribution of orthologs between *S. alba* and *T. bispinosa*. *(c)* The *Sonneratia* group divergence time. Red branches and names represent mangrove species. The blue bars show 95% confidence intervals. The blue star and the number under it mark the whole-genome duplication event. Gray, red, magenta and purple rectangles represent fossil records of inland relatives, mangroves, freshwater genus *Trapa*, and uncertain fossils of *Sonneratia*, respectively. The time ranges of fossils are as follows: 1) earliest confirmed fossils of *D. grandiflora* were wood fossils found in India and Myanmar dating to the middle Miocene [90]; 2) unquestioned modern *Trapa* fruit forms also began in the middle Miocene in Europe, Russia, and Japan [90]; 3) earliest pollen directly related to living species was *S. caseolaris* (*F. levipoli*) from the base of the early Miocene in Borneo (ca. 19 Myr ago) [90,123,124], followed by *S. alba* pollen (*F. meridionalis*) from the middle Miocene in Borneo; 4) wood of *Sonneratioxylon* was from the middle Eocene of Libya (40.4-48.6 Myr ago) [90]; 5) *Sonneratia*-like pollen of *Florshuetzia* sp. was from the late Paleocene of France (Thanetian, 55.8-58.7 Myr ago) [90]; 6) wood of *Sonneratioxylon* was from the early Paleocene of India (Danian, 63.8-67.3 Myr ago) [90]. *(d)* The branch length distribution of syntenic gene pairs from *S. alba* and *S. caseolaris*. The peaks are all at 0.21.

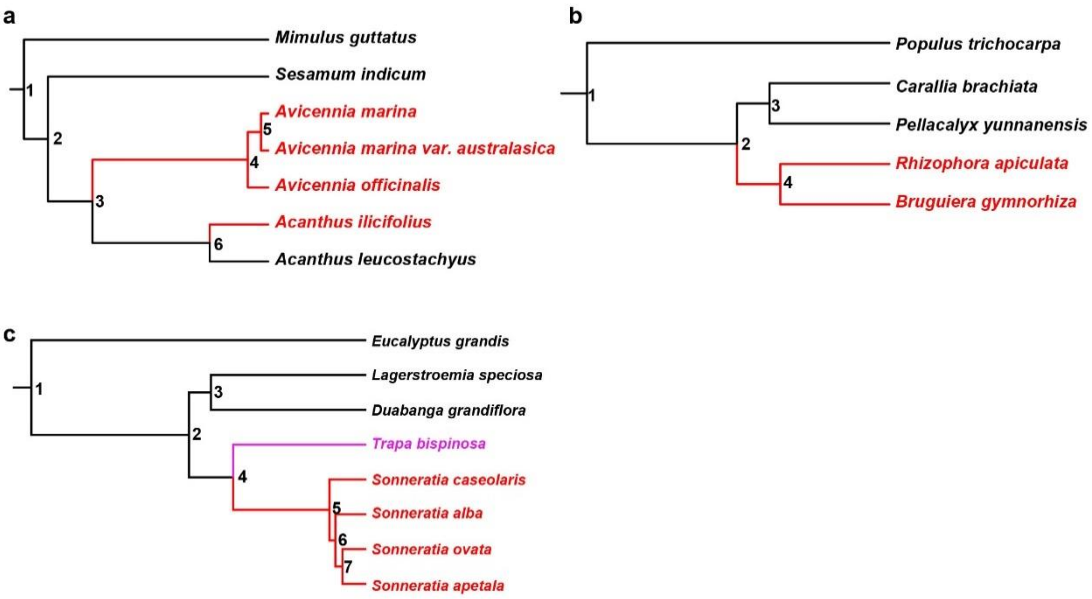

Supplementary Figure 16. The phylogenetic trees of three species groups. *(a) Avicennia* group. *(b)* Rhizophoreae group. *(c) Sonneratia* group. The numbers of each node were used in the Supplementary Tables 15-20.

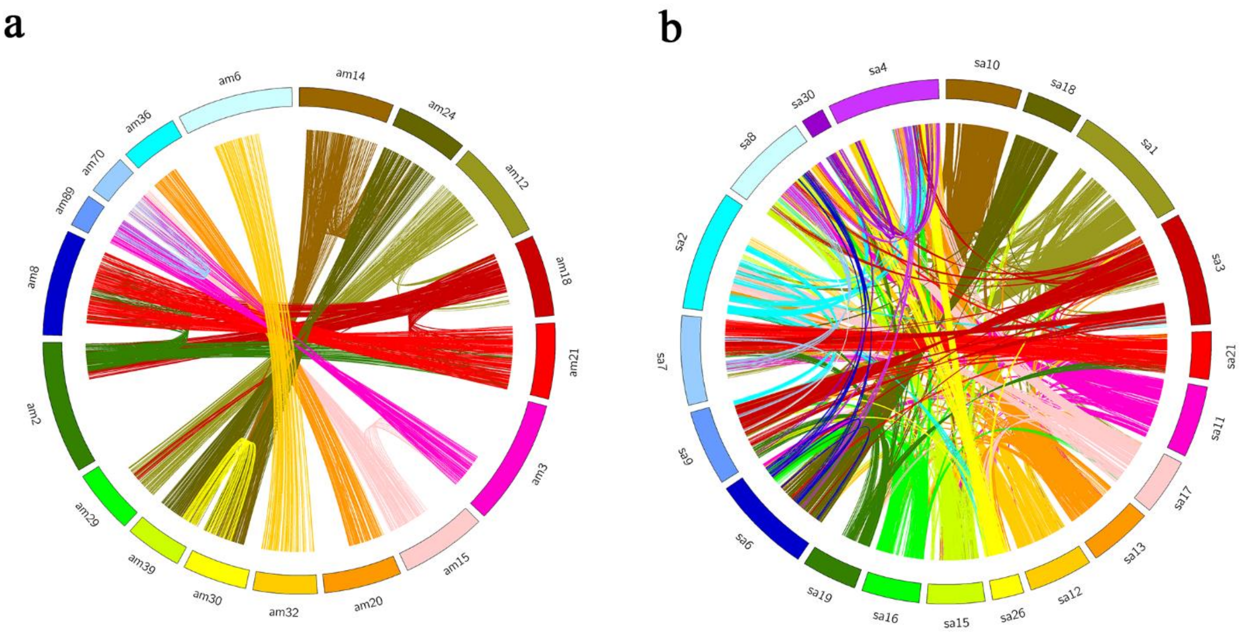

Supplementary Figure 17. Intra-species syntenic blocks in AM and SA. *(a)* Syntenic relationship among the 18 scaffolds containing largest syntenic blocks in *A. marina*. *(b)* Syntenic relationship among the 20 scaffolds containing largest syntenic blocks in *S. alba*. Each line represents a pair of genes in a pair of syntenic blocks.

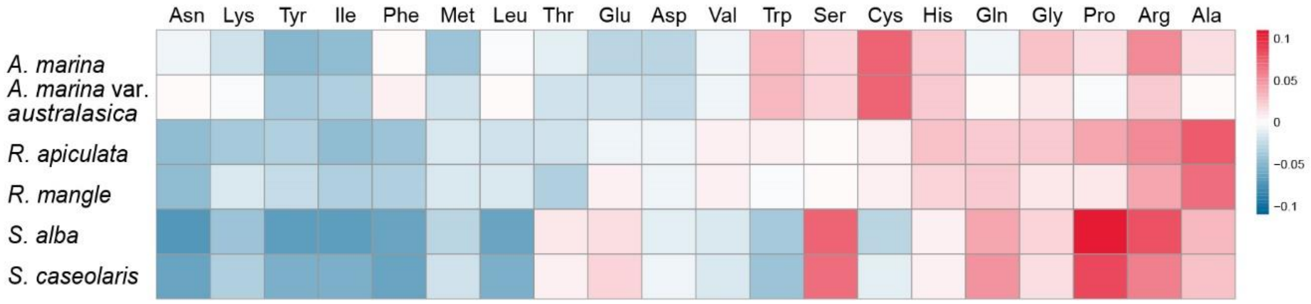

Supplementary Figure 18. **Changes in amino acid frequencies of six mangroves compared to their inland relatives.** Red and blue colors indicate over- and under-representation in the mangrove species.

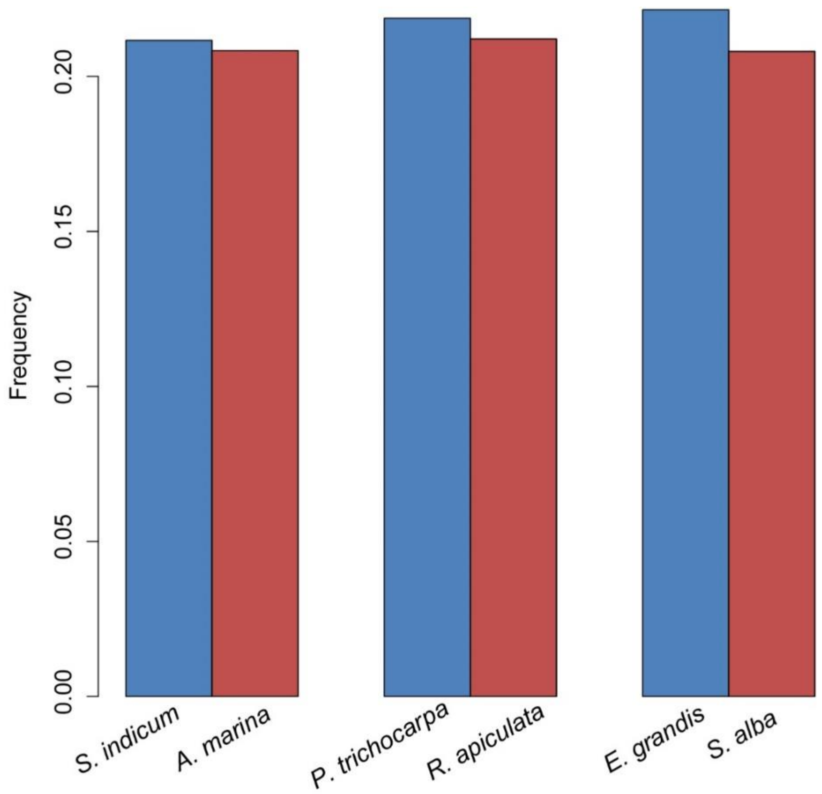

Supplementary Figure 19. Frequency of amino acids with large hydrophobic residues. The frequency of amino acids with large hydrophobic residues (phenylalanine, leucine, isoleucine, and methionine) significantly decreased in *A. marina*, *R. apiculata*, and *S. alba* genomes (P value < 1×10^{-86}, chi-square test).

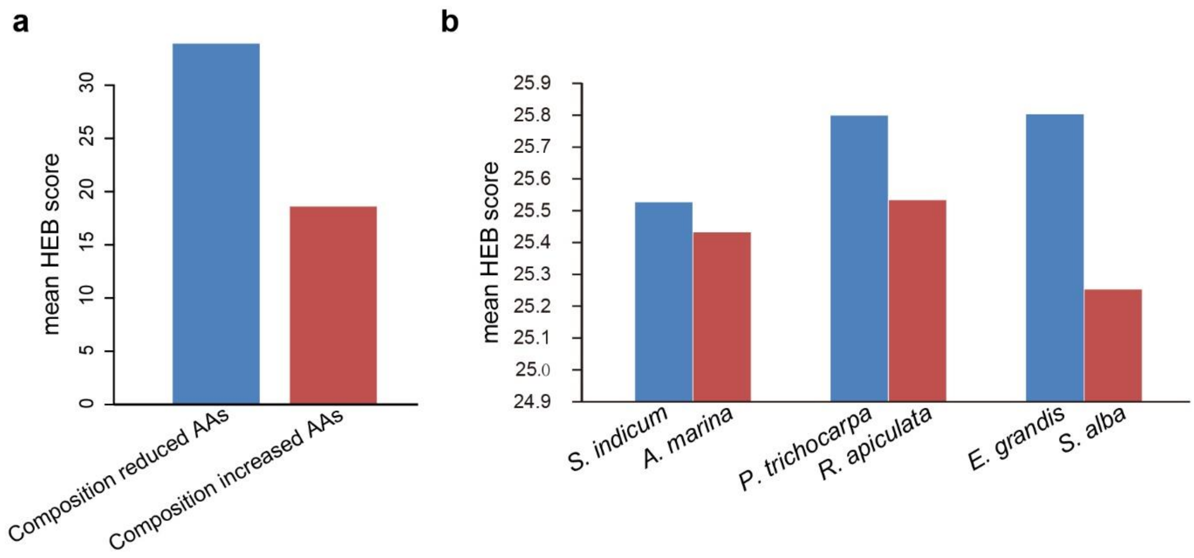

Supplementary Figure 20. Amino acid HEB score. a, Average HEB score of six under-represented AAs and five over-represented AAs. **b**, Average HEB score of three mangroves and their non-mangrove relatives.

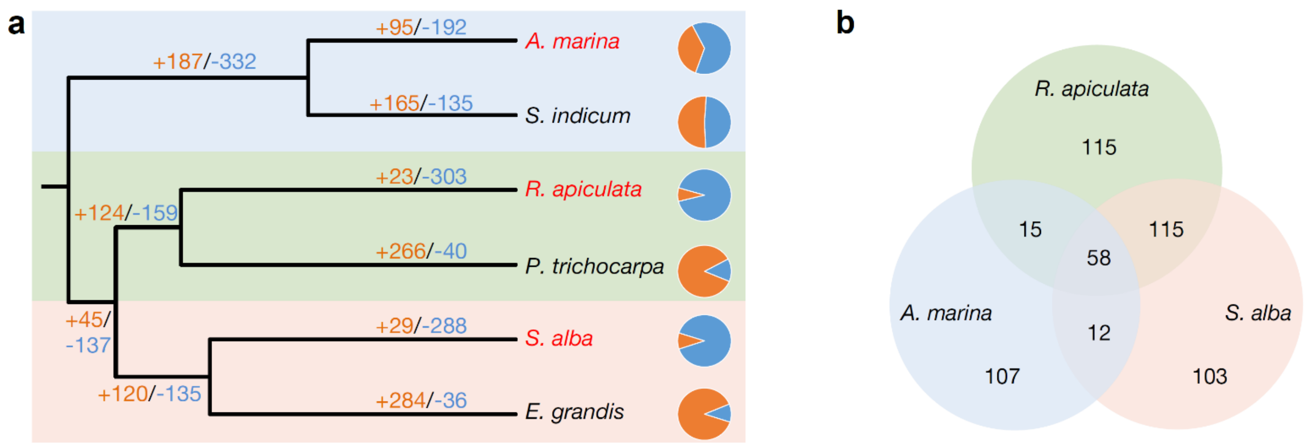

Supplementary Figure 21. Gene family contraction in mangroves. **a**, Gene family expansion and contraction in mangroves (red) and their inland relatives (black). The expanded or contracted gene families was detected by a stochastic birth and death process of software CAFE [87] with p-values < 0.05. The computed numbers for the expanded and contracted gene families are shown above each branch, with rice as the out-group. The split between expansion/contraction along each branch is shown in the pie chart to the right. **b**, Numbers of contracted gene families, shared and unshared, among AM, RA and SA.

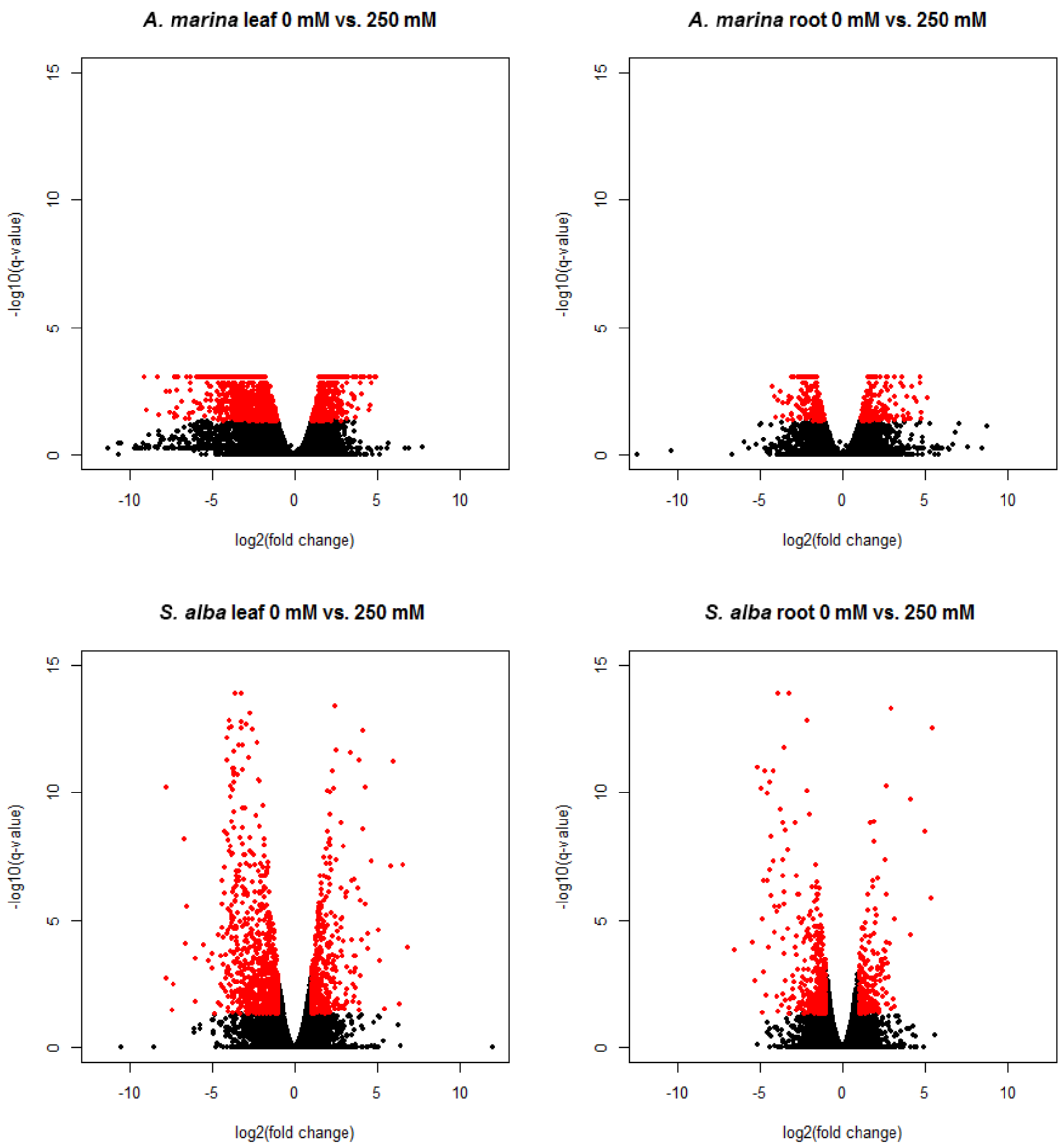

Supplementary Figure 22. Volcano plots of gene expression level changes from 0 to 250 mM NaCl in two mangrove species. Red dots show genes with significant differential expression (q-vaule < 0.05 with fold change greater than 2. Expression pattern in leaves and roots are shown on separate plots.

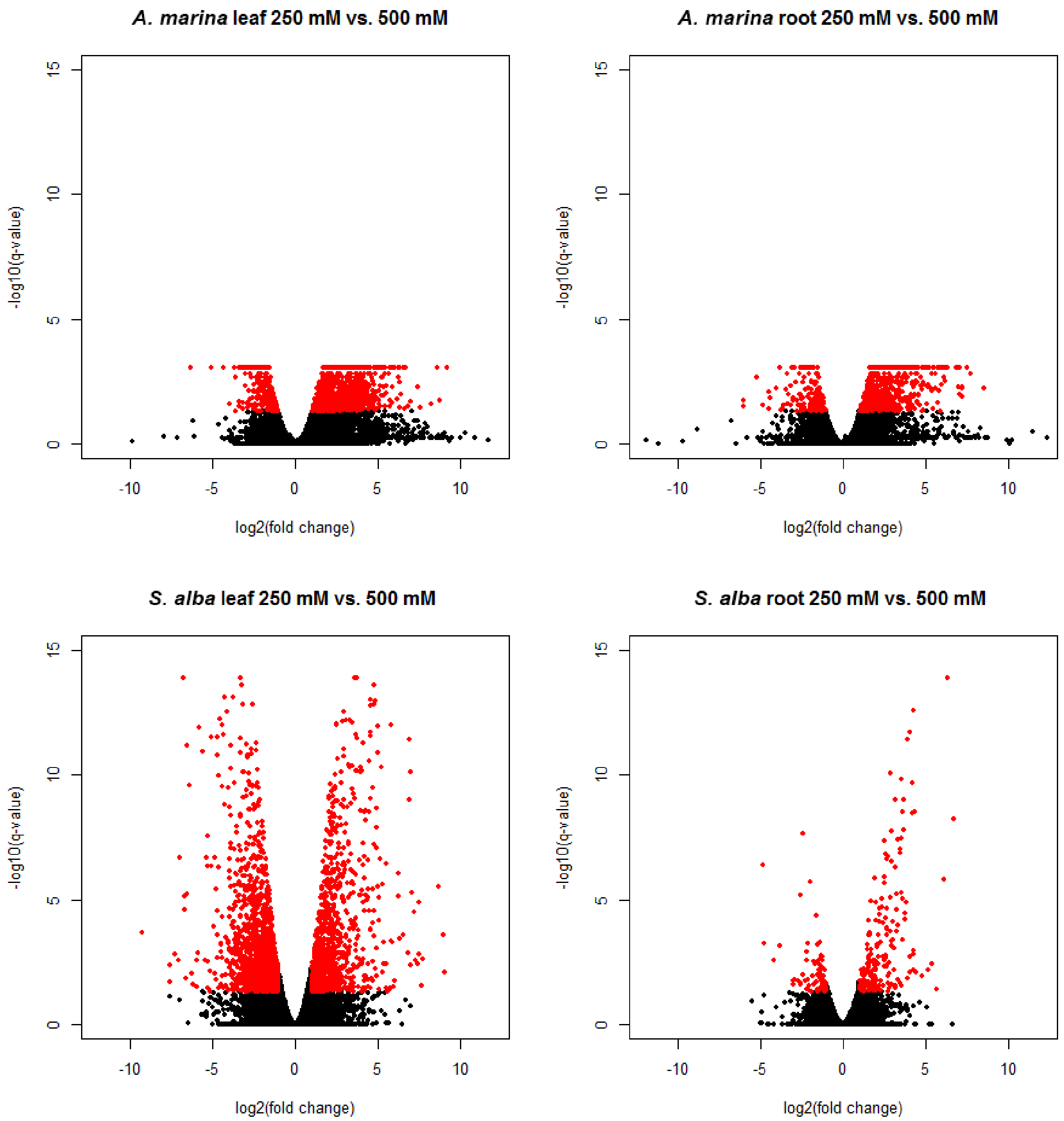

Supplementary Figure 23. Volcano plot of gene expression level changes from 250 to 500 mM NaCl in two mangrove species. Red dots show genes with significant differential expression (q-vaule < 0.05 with fold change greater than 2. Expression pattern in leaves and roots are shown on separate plots.

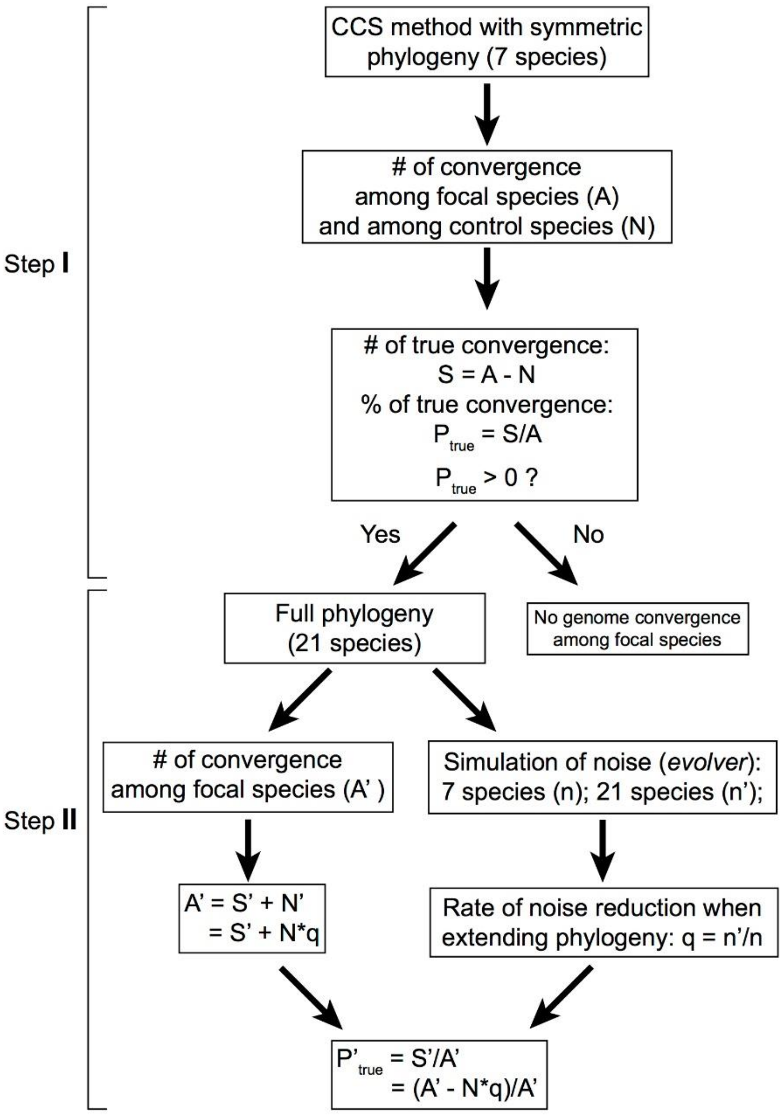

Supplementary Figure 24. Flowchart of CCS+ model in detection of genomic convergence.

Supplementary Tables

Supplementary Table 1. Summary of SMRT sequencing data for *A. marina* and *S. alba*.

		A. marina	***S. alba***
**Before filtering**	Polymerase reads bases	15,710,784,448	28,364,063,716
	Polymerase reads	2,554,964	3,456,716
	Polymerase reads N50	14,184	17,671
**After filtering**	Polymerase reads bases	15,106,982,171	27,208,805,263
	Polymerase reads	1,452,520	2,159,263
	Polymerase reads N50	14,370	17,861

Supplementary Table 2. Summary of DNA libraries and sequencing data for *A. marina*.

Libraries (bp)	Insert size (bp)	Library number	Read length (bp)	Total data (Gb)
200	180-220	1	100	8.6
300	280-320	1	100	7.3
400	380-420	1	100	7.3
600	580-620	1	100	6.4
2,000	500-3,000	2	100	20
5,000	2,000-8,000	2	100	20
10,000	8,000-15,000	2	100	10
Total	-	10	-	79.6

Supplementary Table 3. Summary of DNA libraries and sequencing data for *S. alba*.

Libraries (bp)	Insert size (bp)	Library number	Read length (bp)	Total data (Gb)
200	180-220	1	100	8.3
300	280-320	1	100	7.2
400	380-420	1	100	7.4
600	580-620	1	100	9.9
2,000	500-3,000	3	100	21
5,000	2,000-8,000	3	100	33
10,000	5,000-15,000	2	100	14
Total	-	12	-	100.8

Supplementary Table 4. Genome sizes of mangroves estimated by flow cytometry.

**Order**	**Family**	**Species**	***C*-value (Mb)**
**Lamiales**	Acanthaceae	*Avicennia marina*	508.89
**Malpighiales**	Rhizophoraceae	*Rhizophora apiculata*	273.86
		Bruguiera gymnorhiza	256.89
		Bruguiera sexangula	291.13
		Kandelia candel	186.39
		Ceriops tagal	273.68
**Myrtales**	Lythraceae	*Sonneratia alba*	284.47
		Sonneratia apetala	326.35
		Sonneratia caseolaris	259.43

Supplementary Table 5. Statistics of the final AM and SA genome assemblies.

	Contigs (bp)		Scaffolds (bp)		Chromosome-level scaffolds of *A. marina*
	A. marina	***S. alba***	***A. marina***	***S. alba***	
**Number**	674	343	421	108	32
**N50**	1,542,448	1,893,884	2,339,679	5,523,186	15,140,475
**N90**	415,087	351,148	662,220	1,217,543	11,038,382
**Mean**	679,943	603,806	1,088,625	1,918,222	14,163,629
**Median**	371,606	160,926	592,186	679578.5	14,176,289
**Shortest**	15,080	8,025	16,810	14,540	5,838,278
**Longest**	6,978,494	7,287672,	8,876,880	10,795,034	22,651,104
**Total length**	458,281,843	207,105,377	458,310,972	207,167,948	453,236,130

Supplementary Table 6. Summary of genome completeness assessment.

Species	Transcripts mapping		BUSCO genes mapping		Sanger sequences mapping	
	Number of transcripts	Mapped transcripts	Number of BUSCO genes	Mapped BUSCO genes	Number of genes	Genes with unique mapped position
A. marina	241,439	241,225 (99.91%)	2121	2020 (95.2%)	96	96 (100%)
S. alba	226,814	226,604 (99.91%)	2121	2041 (96.2%)	71	69 (97.18%)

Supplementary Table 7. Assembly statistics from published plant genomes.

Species	Sequencing platform	Scaffold				Assembled genome (Mb)	Percentage of genome size (%)	Reference
		Total number	N50 size (Kb)	N90 size (Kb)	Longest (Kb)			
Avicennia marina	Illumina	421	2,340	662.2	8,877	458.3	90.1	This study
Sonneratia alba	Illumina	108	5,523	1217.5	10,795	207.2	72.8	This study
Amborella trichopoda	454, Illumina & Sanger	5,745	4,900	1,200	16,000	706	81.1	Amborella Genome Project [125]
Banana (*Musa acuminata*)	454, Illumina & Sanger	7,513	1,311	54.3	11,965	472	90.3	D'hont et al. [126]
Carrot (*Daucus carota*)	Illumina & Sanger	4,826	12,700	--	30,200	425.6	89.8	Iorizzo et al. [127]
Cocoa (*Theobroma cacao*)	454, Illumina & Sanger	4,792	474	75.5	3,415	327	76.0	Argout et al. [128]
Cotton (*Gossypium raimondii*)	Illumina	4,715	2,284	644.3	12,800	775	64.5	Wang et al. [115]
Cucumber (*Cucumis sativus*)	Sanger & Illumina	47,837	1,145	2.4	--	244	91.2	Huang et al. [129]
Date palm (*Phoenix dactylifera*)	Illumina	57,277	31	--	--	381	57.9	Al-Dous et al. [130]
Foxtail millet (*Setaria italica*)	Illumina	37,727	1,008	257.8	4,590	423	86.3	Wang et al. [131]
Oropetium thomaeum	SMRT	625	2,400	--	--	244	99	Vanburen et al. [132]
Pineapple (*Ananas comosus*)	454, Illumina & SMRT	3,133	11,759	--	24,881	382	72.6	Ming et al. [133]
Seagrass (*Zostera marina*)	Sanger & Illumina	2,228	124	--	2,655	203.9	--	Olsen et al. [134]
Sesamum indicum	Illumina	16,444	2,100	268.2	--	274	81.3	Wang et al. [27]
Pigeonpea (*Cajanus cajan*)	Sanger & Illumina	137,542	516	25.0	48,970	606	72.7	Varshney et al. [135]
Populus euphratica	Illumina	9,673	482	31.5	8,760	497	83.7	Ma et al. [4]
Strawberry (*Fragaria vesca*)	454, Illumina & solid	3,263	1,361	--	3,924	202	87.4	Shulaev et al. [136]
Thellungiella parvula	454 & Illumina	--	--	--	--	137	85.7	Dassanayake et al. [137]
Thellungiella salsuginea	Illumina	2,682	404	--	--	234	89.9	Wu et al. [138]

Supplementary Table 8. Summary of RNA sequencing and assembly.

Species	Insert size (bp)	Read length (bp)	Total data (Gb)	Number of transcripts
A. marina	300	100	18	241,439
S. alba	300	100	23	226,814

Supplementary Table 9. Properties of predicted protein-coding genes in AM and SA.

**Species**	***A. marina***	***S. alba***
**Gene number**	35,168	31,886
**Average gene length (bp)**	3,566	2,645
**Average CDS length (bp)**	1,167	1,205
**Average exon number**	5.2	5.5
**Average exon length (bp)**	224	221
**Average intron length (bp)**	571	322

Supplementary Table 10. Protein-coding gene annotation statistics.

	A. marina	*S. alba*
**Protein number**	35,168	31,886
**Mean length (AA)**	388	402
**Min length (AA)**	101	102
**Median length (AA)**	312	322
**Max length (AA)**	4,919	5,804
**Total length (AA)**	13,640,992	12,811,577
**Annotated number**	32,817	30,360
**Number with KEGG annotation**	22,316	20,227
**Number with GO annotation**	20,627	18,590

AA, amino acid.

Supplementary Table 11. Transcription factors in AM and SA.

Transcription factor	*A. marina*	*S. alba*
**MYB**	251	176
**C2H2**	181	143
**bHLH**	173	168
**HB**	156	135
**AP2-EREBP**	144	229
**WRKY**	121	109
**bZIP**	112	110
**C3H**	98	84
**NAC**	96	92
**GRAS**	74	75
**LOB**	70	67
**CCAAT**	67	69
**G2-like**	66	74
**MADS**	65	15
**C2C2-Dof**	60	57
**Trihelix**	51	45
**TCP**	48	44
**ABI3VP1**	44	40
**ARF**	42	33
**HSF**	41	32
**C2C2-GATA**	41	43
**OFP**	33	28
**mTERF**	33	31
**FAR1**	27	22
**SBP**	25	29
**Tify**	20	20
**zf-HD**	20	21
**GRF**	18	12
**PLATZ**	18	21

**TUB**	17	16
**RWP-RK**	16	10
**FHA**	16	18
**ARR-B**	15	13
**SRS**	13	11
**GeBP**	13	6
**Alfin-like**	12	10
**C2C2-YABBY**	11	8
**BES1**	11	11
**BSD**	11	13
**E2F-DP**	10	12
**LIM**	10	13
**Sigma70-like**	8	6
**C2C2-CO-like**	8	17
**BBR/BPC**	8	11
**CPP**	7	8
**TAZ**	7	8
**CAMTA**	6	8
**EIL**	6	7
**CSD**	5	6
**LFY**	4	1
**DBP**	3	3
**PBF-2-like**	3	3
**VOZ**	3	3
**NOZZLE**	2	2
**S1Fa-like**	2	1
**SAP**	2	2
**HRT**	1	1
**ULT**	1	2
**Total**	2,426	2,254

Supplementary Table 12. AM and SA repeat element statistics.

Type	*A. marina*		*S. alba*	
	**Copy Number**	**% of genome**	**Copy Number**	**% of genome**
**DNA**	24,341	0.800	14,889	3.016
hAT-Ac	5,046	0.121	3,929	0.649
hAT-Tag1	6,906	0.218	4,937	1.052
MULE-MuDR	3,720	0.070	332	0.089
PIF-Harbinger	899	0.039	899	0.358
CMC-EnSpm	4,507	0.224	724	0.163
others	3,263	0.128	4,068	0.705
**LTR**	53,037	11.013	12,312	13.111
Copia	24,819	4.093	4,885	2.052
Gypsy	25,141	6.537	6,605	10.914
Caulimovirus	1,868	0.312	0	0.000
others	1,209	0.070	822	0.145
**LINE**	1,794	0.167	2,731	0.609
**SINE**	39	0.001	279	0.099
**Others**	248,341	5.271	37,355	4.716
**Total**	327,552	17.251	67,566	21.552

Supplementary Table 13. Summary of gene family clustering.

Species	Total genes	Families	Species-specific families	Genes of species-specific families	Genes per family
A. marina	35,168	13,850	544	1,710	2.54
R. apiculata	26,640	13,151	469	1,360	2.03
S. alba	31,886	13,064	782	2,045	2.44
S. indicum	27,148	13,422	525	3,440	2.02
P. trichocarpa	41,335	14,986	1,170	4,043	2.76
E. grandis	36,374	13,846	916	3,652	2.63
O. sativa	39,046	12,325	2,489	8,312	3.17

Supplementary Table 14. Datasets used in the divergence time estimation and whole-genome duplication dating.

Species	Data type	Number of protein-coding genes	Reference
**Avicennia group**			
Avicennia marina	genome	35,168	This study
Avicennia marina var. australasica	genome	31,801	This study
Mimulus guttatus	genome	26,718	Hellsten et al. [26]
Sesamum indicum	genome	27,148	Wang et al. [27]
Avicennia officinalis	transcriptome	50,654	This study
Acanthus ilicifolius	transcriptome	50,442	Yang et al. [28]
Acanthus leucostachyus	transcriptome	43,337	Yang et al. [28]
**Rhizophoreae group**			
Rhizophora apiculata	genome	26,640	This study
Bruguiera gymnorhiza	genome	24,890	Li *et al.*, unpublished
Populus trichocarpa	genome	41,335	Tuskan et al. [5]
Carallia brachiata	transcriptome	41,653	Guo et al. [30]
Pellacalyx yunnanensis	transcriptome	29,851	Yang et al. [29]
**Sonneratia group**			
Eucalyptus grandis	genome	36,374	Myburg et al. [3]
Sonneratia alba	genome	31,949	This study
Sonneratia caseolaris	genome	28,077	This study
Trapa bispinosa	transcriptome	66,320	Li et al. [31]
Duabanga grandiflora	transcriptome	52,317	Li et al. [31]
Lagerstroemia speciosa	transcriptome	40,705	This study
Sonneratia apetala	transcriptome	48,789	This study
Sonneratia ovata	transcriptome	42,204	This study
**Outgroup**			
Oryza sativa	genome	39,049	Ouyang et al. [91]

Supplementary Table 15. Divergence times and confidence intervals of *Avicennia* group nodes using *MCMCTREE*.

Node	codon123	codon12
**1**	72.6 [70.0,75.0]	72.5 [70.0,75.0]
**2**	66.5 [57.8,73.4]	66.3 [55.4,73.4]
**3**	53.1 [45.7,60.3]	52.9 [42.8,60.4]
**4**	6.7 [4.9,9.1]	7.6 [5.5,10.0]
**5**	1.9 [1.3,2.5]	2.1 [1.5,2.9]
**6**	16.6 [12.5,21.8]	17.4 [12.5,22.4]

Divergence time and 95% confidence interval of each node was shown. Time unit: million years. Nodes were marked in Supplementary Fig. 16.

Codon123, the datasets include all three codon positions. Codon12, the dataset includes the first and second codon positions.

Supplementary Table 16. Divergence time and confidence intervals of Rhizophoreae group nodes using *MCMCTREE*.

Node	codon123	codon12
**1**	117.2 [109.1,120.7]	116.0 [106.5,120.5]
**2**	54.1 [49.1,60.7]	54.5 [48.7,64.5]
**3**	41.7 [36.4,48.3]	42.5 [36.2,51.3]
**4**	38.5 [36.0,42.2]	39.4 [36.4,45.9]

Divergence time and 95% confidence interval of each node was shown. Time unit: million years. Nodes were marked in Supplementary Fig. 16.

Codon123, the datasets include all three codon positions. Codon12, the dataset includes the first and second codon positions.

Supplementary Table 17. Divergence time and confidence intervals of *Sonneratia* group nodes using *MCMCTREE*.

Node	codon123	codon12
**1**	107.0 [100.8,110.3]	106.4 [100.4,110.2]
**2**	57.5 [53.1,64.5]	57.8 [52.7,66.2]
**3**	49.7 [47.2,55.0]	50.3 [47.4,57.1]
**4**	42.5 [36.4,49.4]	43.5 [36.2,51.7]
**5**	8.9 [7.0,11.1]	9.8 [7.3,12.8]
**6**	7.2 [5.6,9.2]	8.1 [5.9,10.7]
**7**	5.3 [3.8,7.1]	6.7 [4.1,8.3]

Divergence time and 95% confidence interval of each node was shown. Time unit: million years. Nodes were marked in Supplementary Fig. 16.

Codon123, the datasets include all three codon positions. Codon12, the datasets include the first and second codon positions.

Supplementary Table 18. Divergence time of *Avicennia* group nodes using *r8s*.

Node	Results of MCMCTREE	HKY85+G in r8s			GTR+I+G in r8s		
		PL	LF	NPRS	PL	LF	NPRS
**1**	72.6 [70.0,75.0]	75.00	75.00	75.00	75.00	75.00	75.00
**2**	66.5 [57.8,73.4]	46.93	46.99	45.43	47.06	47.09	45.48
**3**	53.1 [45.7,60.3]	39.10	39.17	36.38	39.32	39.36	36.53
**4**	6.7 [4.9,9.1]	5.16	5.17	5.66	5.23	5.23	5.71
**5**	1.9 [1.3,2.5]	1.34	1.35	1.53	1.36	1.36	1.55
**6**	16.6 [12.5,21.8]	10.86	10.90	8.46	10.99	11.02	8.56

Nodes were marked in Supplementary Fig. 16. Time unit: million years.

PL, LF and NPRS, methods used in *r8s*.

Supplementary Table 19. Divergence time of Rhizophoreae group nodes using *r8s*.

Node	Results of MCMCTREE	HKY85+G in r8s			GTR+I+G in r8s		
		PL	LF	NPRS	PL	LF	NPRS
**1**	117.2 [109.1,120.7]	120.00	120.00	120.00	120.00	120.00	120.00
**2**	54.1 [49.1,60.7]	45.55	44.47	51.23	45.57	45.50	51.27
**3**	41.7 [36.4,48.3]	32.56	32.40	41.48	32.57	32.44	41.53
**4**	38.5 [36.0,42.2]	38.00	38.00	38.00	38.00	38.00	38.00

Nodes were marked in Supplementary Fig. 16. Time unit: million years.

PL, LF and NPRS, methods used in *r8s*.

Supplementary Table 20. Divergence time of *Sonneratia* group nodes using *r8s*.

Node	Results of MCMCTREE	HKY85+G in r8s			GTR+I+G in r8s		
		PL	LF	NPRS	PL	LF	NPRS
**1**	107.0 [100.8,110.3]	110.00	110.00	110.00	110.00	110.00	110.00
**2**	57.5 [53.1,64.5]	50.87	50.69	53.64	50.86	50.71	53.65
**3**	49.7 [47.2,55.0]	47.80	47.80	47.80	47.80	47.80	47.80
**4**	42.5 [36.4,49.4]	37.37	36.69	43.90	37.32	36.74	43.89
**5**	8.9 [7.0,11.1]	6.09	5.78	13.93	6.06	5.80	13.87
**6**	7.2 [5.6,9.2]	4.60	4.35	11.60	4.58	4.37	11.55
**7**	5.3 [3.8,7.1]	2.99	2.82	8.31	2.97	2.83	8.27

Nodes were marked in Supplementary Fig. 16. Time unit: million years.

PL, LF and NPRS, methods used in *r8s*.

Supplementary Table 21. Functional categorization of 73 candidate mangrove-convergent genes.

Functional categories	Orthologous genes of *S. alba*
response to stress	SA_06167 SA_11584 SA_03006 SA_16354 SA_00230 SA_03953 SA_12276 SA_02407 SA_05795 SA_20423 SA_11519 SA_05832 SA_19915 SA_28155 SA_14269 SA_19139 SA_08456 SA_02934 SA_06975 SA_01211 SA_21991
metabolic processes	SA_21057 SA_16089 SA_01596 SA_17374 SA_26511 SA_11137 SA_28822 SA_05385 SA_01658 SA_07883 SA_12268 SA_04100 SA_02226 SA_11060 SA_07439 SA_14528 SA_14765
developmental processes	SA_16716 SA_20131 SA_11233 SA_03524 SA_00738 SA_23432 SA_12019 SA_18589 SA_15247 SA_16352 SA_12025 SA_23335 SA_13105 SA_09087
other cellular processes	SA_03969 SA_16740 SA_28015 SA_18583 SA_25861
other metabolic processes	SA_07696
unknown biological processes	SA_27615 SA_05450 SA_24734 SA_24178 SA_06762 SA_14702 SA_06321 SA_18018 SA_13508 SA_28187 SA_01668 SA_05676 SA_22190 SA_23928 SA_08436

Supplementary Table 22. KEGG pathway annotation of 73 candidate mangrove-convergent genes.

PathwayID	No. of mangrove convergence genes	Orthologous genes of *S. alba*	Description
PATH:ko04120	4	SA_16354 SA_20423 SA_05832 SA_08436	Ubiquitin mediated proteolysis
PATH:ko04626	4	SA_09087 SA_23432 SA_28155 SA_28187	Plant-pathogen interaction
PATH:ko00680	4	SA_14269 SA_18589 SA_01596 SA_05450	Methane metabolism
PATH:ko03040	3	SA_14702 SA_01658 SA_28822	Spliceosome
PATH:ko04110	3	SA_15247 SA_08436 SA_08456	Cell cycle
PATH:ko00900	3	SA_19915 SA_03524 SA_05385	Terpenoid backbone biosynthesis
PATH:ko00230	2	SA_00230 SA_19139	Purine metabolism
PATH:ko03420	2	SA_19139 SA_20423	Nucleotide excision repair
PATH:ko03013	2	SA_12025 SA_16089	RNA transport
PATH:ko04144	2	SA_16740 SA_18583	Endocytosis
PATH:ko03010	2	SA_16352 SA_22190	Ribosome
PATH:ko00940	2	SA_14269 SA_06321	Phenylpropanoid biosynthesis
PATH:ko00363	2	SA_26511 SA_02934	Bisphenol degradation
PATH:ko00360	2	SA_14269 SA_06321	Phenylalanine metabolism
PATH:ko00040	1	SA_13508	Pentose and glucuronate interconversions
PATH:ko00520	1	SA_13105	Amino sugar and nucleotide sugar metabolism
PATH:ko00625	1	SA_02934	Chloroalkane and chloroalkene degradation
PATH:ko04620	1	SA_18018	Toll-like receptor signaling pathway
PATH:ko00710	1	SA_00230	Carbon fixation in photosynthetic organisms
PATH:ko00460	1	SA_01596	Cyanoamino acid metabolism
PATH:ko03030	1	SA_19139	DNA replication
PATH:ko00020	1	SA_12276	Citrate cycle (TCA cycle)

PATH:ko03410	1	SA_19139	Base excision repair
PATH:ko00240	1	SA_19139	Pyrimidine metabolism
PATH:ko03018	1	SA_02226	RNA degradation
PATH:ko00960	1	SA_07696	Tropane, piperidine and pyridine alkaloid biosynthesis
PATH:ko04066	1	SA_03969	HIF-1 signaling pathway
PATH:ko00260	1	SA_01596	Glycine, serine and threonine metabolism
PATH:ko00010	1	SA_00230	Glycolysis / Gluconeogenesis
PATH:ko00903	1	SA_26511	Limonene and pinene degradation
PATH:ko00052	1	SA_05676	Galactose metabolism
PATH:ko00600	1	SA_05676	Sphingolipid metabolism
PATH:ko03015	1	SA_11519	mRNA surveillance pathway
PATH:ko00280	1	SA_27615	Valine, leucine and isoleucine degradation
PATH:ko00190	1	SA_12276	Oxidative phosphorylation
PATH:ko04670	1	SA_03969	Leukocyte transendothelial migration
PATH:ko00630	1	SA_01596	Glyoxylate and dicarboxylate metabolism
PATH:ko00905	1	SA_03006	Brassinosteroid biosynthesis
PATH:ko04115	1	SA_08456	p53 signaling pathway
PATH:ko04141	1	SA_12025	Protein processing in endoplasmic reticulum
PATH:ko00650	1	SA_02934	Butanoate metabolism
PATH:ko00982	1	SA_18589	Drug metabolism - cytochrome P450
PATH:ko04310	1	SA_11137	Wnt signaling pathway
PATH:ko04064	1	SA_18018	NF-kappa B signaling pathway
PATH:ko04145	1	SA_03969	Phagosome
PATH:ko04964	1	SA_07439	Proximal tubule bicarbonate reclamation
PATH:ko00100	1	SA_02407	Steroid biosynthesis
PATH:ko00790	1	SA_04100	Folate biosynthesis
PATH:ko04130	1	SA_20131	SNARE interactions in vesicular transport
PATH:ko04722	1	SA_18018	Neurotrophin signaling pathway
PATH:ko04976	1	SA_05385	Bile secretion
PATH:ko03022	1	SA_23928	Basal transcription factors
PATH:ko00053	1	SA_13105	Ascorbate and aldarate metabolism
PATH:ko00511	1	SA_05676	Other glycan degradation

PATH:ko04721	1	SA_20131	Synaptic vesicle cycle
PATH:ko00624	1	SA_26511	Polycyclic aromatic hydrocarbon degradation
PATH:ko00051	1	SA_02934	Fructose and mannose metabolism
PATH:ko00590	1	SA_12268	Arachidonic acid metabolism
PATH:ko00945	1	SA_26511	Stilbenoid, diarylheptanoid and gingerol biosynthesis
PATH:ko04622	1	SA_05450	RIG-I-like receptor signaling pathway
PATH:ko00591	1	SA_02934	Linoleic acid metabolism
PATH:ko00130	1	SA_06321	Ubiquinone and other terpenoid-quinone biosynthesis
PATH:ko00627	1	SA_26511	Aminobenzoate degradation
PATH:ko00561	1	SA_05450	Glycerolipid metabolism
PATH:ko00620	1	SA_00230	Pyruvate metabolism
PATH:ko00402	1	SA_03953	Benzoxazinoid biosynthesis
PATH:ko04210	1	SA_18018	Apoptosis
PATH:ko00670	1	SA_01596	One carbon pool by folate

Supplementary Table 23. Four of the 73 candidate mangrove-convergent genes that were assigned to the "ubiquitin mediated proteolysis" pathway.

Orthologous genes of *S. alba*	KEGG Orthology term	Gene Name	Description	# of mangrove convergent sites
SA_20423	K10610	DDB1	DNA damage-binding protein 1	2
SA_05832	K10260	FBXW7	F-box and WD-40 domain protein 7	3
SA_08436	K03354	APC7	anaphase-promoting complex subunit 7	2
SA_16354	K10260	FBXW7	F-box and WD-40 domain protein 7	2

Supplementary Table 24. Comparison of GC content of introns and coding regions between mangrove species and their inland relatives.

Mangrove species	GC content (%)	Related non-mangrove	GC content (%)	GC content increase in mangroves (%)
**Intron**				
A. marina	33.14	*S. indicum*	33.90	-0.76
R. apiculata	34.62	*P. trichocarpa*	33.44	1.18
S. alba	37.39	*E. grandis*	37.35	0.04
**Coding region**				
A. marina	46.85	*S. indicum*	45.94	0.91
R. apiculata	45.43	*P. trichocarpa*	43.47	1.96
S. alba	50.13	*E. grandis*	48.59	1.54

Supplementary Table 25. Adjusted CDS GC content in mangroves and related non-mangroves.

Mangrove species	Adjusted GC content of CDS (%)	Related non-mangrove	Adjusted GC content of CDS (%)	GC content increase in mangroves (%)
A. marina	47.67	*S. indicum*	47.02	0.65
R. apiculata	46.80	*P. trichocarpa*	45.27	1.53
S. alba	50.36	*E. grandis*	48.45	1.91

Supplementary Table 26. The 58 gene families contracted in the *A. marina, R. apiculata* and *S. alba*.

Gene family ID	Number of genes in each species							Annotation Description
	Osa	Sin	Ama	Ptr	Rap	Egr	Sal	
GF_2	75	12	6	31	3	199	2	Leucine-rich repeat protein kinase family protein
GF_3	52	61	30	68	31	67	13	Subtilase family protein
GF_5	19	26	25	53	14	76	33	laccase 14
GF_8	40	14	10	46	10	82	20	Wall-associated kinase family protein
GF_14	8	28	23	64	1	31	8	FAD-binding Berberine family protein
GF_19	41	4	5	24	7	50	14	receptor lectin kinase
GF_21	17	3	4	31	1	84	0	PR5-like receptor kinase
GF_25	16	11	2	39	1	50	11	UDP-glucosyl transferase 85A2
GF_27	16	2	0	29	0	60	15	RmlC-like cupins superfamily protein
GF_30	18	16	10	34	17	20	2	beta glucosidase 17
GF_35	48	13	7	22	3	17	2	cytochrome P450, family 71, subfamily B, polypeptide 34
GF_54	6	16	12	20	6	28	1	cytochrome P450, family 81, subfamily D, polypeptide 8
GF_68	1	23	6	31	2	16	3	ATP binding;nucleic acid binding;helicases
GF_69	9	9	7	21	9	19	8	acyl activating enzyme 1
GF_79	1	14	8	5	1	38	10	terpene synthase 21
GF_97	0	8	6	19	1	31	4	cytochrome P450, family 82, subfamily C, polypeptide 4
GF_107	0	11	8	22	0	21	3	UDP-Glycosyltransferase superfamily protein
GF_129	18	14	3	11	3	7	3	O-methyltransferase family protein
GF_136	5	13	4	7	3	21	5	cytochrome P450, family 76, subfamily C, polypeptide 4
GF_146	0	8	4	9	4	30	1	Cytochrome P450 superfamily protein
GF_150	3	5	2	14	2	29	1	Protein kinase superfamily protein

GF_154	6	6	5	16	3	13	6	Leucine-rich repeat protein kinase family protein
GF_182	21	3	1	8	0	16	2	SAUR-like auxin-responsive protein family
GF_199	1	3	2	18	1	20	4	Zinc-binding dehydrogenase family protein
GF_208	1	6	3	19	5	9	4	HXXXD-type acyl-transferase family protein
GF_217	4	10	1	4	4	19	4	HSP20-like chaperones superfamily protein
GF_235	10	3	1	2	1	24	3	P-loop containing nucleoside triphosphate hydrolases superfamily protein
GF_283	3	7	5	6	1	17	1	NAD(P)-linked oxidoreductase superfamily protein
GF_332	1	5	3	16	1	7	4	terpene synthase-like sequence-1,8-cineole
GF_342	2	4	3	17	4	3	3	O-Glycosyl hydrolases family 17 protein
GF_351	3	6	3	11	3	8	1	MATE efflux family protein
GF_360	1	3	2	15	3	11	0	Pectin lyase-like superfamily protein
GF_363	0	7	2	13	0	11	2	Cytochrome P450 superfamily protein
GF_377	7	6	4	1	1	14	1	cytochrome P450, family 71, subfamily A, polypeptide 26
GF_384	1	8	5	7	2	10	1	Pectin lyase-like superfamily protein
GF_394	4	6	2	11	1	7	3	senescence-associated gene 12
GF_416	3	2	1	5	3	16	3	indole-3-acetate beta-D-glucosyltransferase
GF_529	6	5	3	11	1	2	1	Major facilitator superfamily protein
GF_566	0	10	6	2	1	8	1	cellulose synthase like G2
GF_666	2	2	1	4	1	14	2	cytochrome BC1 synthesis
GF_764	2	12	1	6	2	1	0	SAUR-like auxin-responsive protein family
GF_780	0	11	4	8	0	0	0	cytochrome P450, family 83, subfamily B, polypeptide 1
GF_817	0	5	3	5	0	10	0	myb domain protein 113
GF_821	0	2	0	7	0	12	2	Arabidopsis protein of unknown function (DUF241)
GF_826	0	1	0	8	0	14	0	TRAF-like family protein
GF_891	0	1	0	4	2	14	1	general control non-repressible 4
GF_893	0	2	1	5	1	11	2	Arabidopsis protein of unknown function

								(DUF241)
GF_894	7	6	1	8	0	0	0	Peroxidase superfamily protein
GF_961	2	3	2	8	0	5	1	germin-like protein subfamily 2 member 2 precursor
GF_964	3	2	1	1	0	13	1	homolog of Medicago truncatula MTN3
GF_1056	2	2	1	1	0	12	2	S-adenosyl-L-methionine-dependent methyltransferases superfamily protein
GF_1147	1	1	0	2	0	13	2	NmrA-like negative transcriptional regulator family protein
GF_1162	2	2	1	5	0	8	1	homolog of carrot EP3-3 chitinase
GF_1291	0	2	1	8	1	5	1	D-mannose binding lectin protein with Apple-like carbohydrate-binding domain
GF_1439	0	4	1	5	0	7	0	2-oxoglutarate (2OG) and Fe(II)-dependent oxygenase superfamily protein
GF_2028	0	1	0	6	0	6	1	cytochrome P450, family 712, subfamily A, polypeptide 1
GF_2475	1	8	0	3	0	1	0	SGNH hydrolase-type esterase superfamily protein
GF_2806	0	2	0	4	0	6	0	NAD(P)-binding Rossmann-fold superfamily protein

Sal, *S. alba*; Egr, *E. grandis*; Rap, *R. apiculata*; Ptr, *P. trichocarpa*; Ama, *A. marina*; Sin, *S. indicum*; Osa, *O. sativa*.

* Gene families pertaining to disease resistance are contracted in *A. marina*, *R. apiculata* and *S. alba*.

Supplementary Table 27. Contracted gene families that related to disease resistance in at least one mangrove genome.

Gene family ID	Annotation description
GF_4	disease resistance protein (TIR-NBS-LRR class), putative
GF_36	NB-ARC domain-containing disease resistance protein
GF_38	NB-ARC domain-containing disease resistance protein
GF_53	NB-ARC domain-containing disease resistance protein
GF_94	Disease resistance protein (TIR-NBS-LRR class) family
GF_105	NB-ARC domain-containing disease resistance protein
GF_109	disease resistance family protein / LRR family protein
GF_124	Disease resistance protein (TIR-NBS-LRR class) family
GF_143	NB-ARC domain-containing disease resistance protein
GF_168	NB-ARC domain-containing disease resistance protein
GF_169	NB-ARC domain-containing disease resistance protein
GF_202	disease resistance protein (TIR-NBS-LRR class), putative
GF_209	NB-ARC domain-containing disease resistance protein
GF_226	Disease resistance protein (CC-NBS-LRR class) family
GF_234	disease resistance protein (TIR-NBS-LRR class), putative
GF_276	NB-ARC domain-containing disease resistance protein
GF_298	NB-ARC domain-containing disease resistance protein
GF_322	NB-ARC domain-containing disease resistance protein
GF_335	Disease resistance protein (TIR-NBS-LRR class) family
GF_348	NB-ARC domain-containing disease resistance protein
GF_349	disease resistance protein (TIR-NBS-LRR class)
GF_362	disease resistance family protein / LRR family protein
GF_419	disease resistance protein (TIR-NBS-LRR class), putative
GF_438	LRR and NB-ARC domains-containing disease resistance protein
GF_442	NB-ARC domain-containing disease resistance protein
GF_464	NB-ARC domain-containing disease resistance protein
GF_530	NB-ARC domain-containing disease resistance protein
GF_572	NB-ARC domain-containing disease resistance protein

GF_658	Disease resistance protein (CC-NBS-LRR class) family
GF_698	disease resistance family protein / LRR family protein
GF_959	Disease resistance protein (TIR-NBS-LRR class) family
GF_1063	NB-ARC domain-containing disease resistance protein
GF_1410	Disease resistance protein (TIR-NBS-LRR class) family
GF_1586	disease resistance protein (TIR-NBS-LRR class)
GF_1603	Disease resistance protein (TIR-NBS-LRR class) family
GF_1610	NB-ARC domain-containing disease resistance protein
GF_1850	NB-ARC domain-containing disease resistance protein
GF_2034	disease resistance protein (TIR-NBS-LRR class)
GF_2036	Disease resistance protein (TIR-NBS-LRR class) family
GF_2371	disease resistance protein (TIR-NBS-LRR class)
GF_2396	Disease resistance protein (TIR-NBS-LRR class) family
GF_2801	Disease resistance protein (CC-NBS-LRR class) family
GF_2809	LRR and NB-ARC domains-containing disease resistance protein
GF_2826	Disease resistance protein (TIR-NBS class)
GF_3478	Disease resistance protein (TIR-NBS-LRR class) family
GF_3503	Disease resistance protein (TIR-NBS-LRR class) family
GF_3523	NB-ARC domain-containing disease resistance protein
GF_4723	NB-ARC domain-containing disease resistance protein
GF_5634	NB-ARC domain-containing disease resistance protein
GF_5791	NB-ARC domain-containing disease resistance protein
GF_7088	Disease resistance protein (TIR-NBS-LRR class) family

Supplementary Table 28. Pathways significantly enriched in genes differentially expressed under salt treatment (DEGs).

Pathway	Total No. of genes in the pathway	No. of DEGs in the pathway	p-value	q-value	Annotation
***A. marina* leaves, from 0 mM to 250 mM**					
PATH:ko04626	766	80	7.11E-13	1.22E-10	Plant-pathogen interaction
PATH:ko00941	126	19	2.47E-06	2.12E-04	Flavonoid biosynthesis
PATH:ko04722	990	75	3.19E-06	1.83E-04	Neurotrophin signaling pathway
PATH:ko00950	138	19	9.64E-06	4.14E-04	Isoquinoline alkaloid biosynthesis
PATH:ko00902	52	11	1.25E-05	4.29E-04	Monoterpenoid biosynthesis
PATH:ko04620	887	66	2.15E-05	6.16E-04	Toll-like receptor signaling pathway
PATH:ko04064	864	64	3.26E-05	8.01E-04	NF-kappa B signaling pathway
PATH:ko04740	69	12	4.09E-05	8.80E-04	Olfactory transduction
PATH:ko04971	50	10	5.15E-05	9.85E-04	Gastric acid secretion
PATH:ko00511	198	22	6.11E-05	1.05E-03	Other glycan degradation
PATH:ko00943	43	9	8.38E-05	1.31E-03	Isoflavonoid biosynthesis
PATH:ko00592	87	13	1.02E-04	1.46E-03	alpha-Linolenic acid metabolism
PATH:ko04970	73	11	3.17E-04	4.20E-03	Salivary secretion
PATH:ko04744	42	8	4.11E-04	5.04E-03	Phototransduction
PATH:ko00945	154	17	4.33E-04	4.96E-03	Stilbenoid, diarylheptanoid and gingerol biosynthesis
PATH:ko04713	77	11	5.08E-04	5.46E-03	Circadian entrainment
PATH:ko04261	138	15	1.08E-03	1.09E-02	Adrenergic signaling in cardiomyocytes
PATH:ko00940	326	27	1.23E-03	1.17E-02	Phenylpropanoid biosynthesis
PATH:ko04075	735	50	1.49E-03	1.35E-02	Plant hormone signal transduction
PATH:ko04750	52	8	1.78E-03	1.53E-02	Inflammatory mediator regulation of TRP channels
PATH:ko04745	53	8	2.02E-03	1.65E-02	Phototransduction - fly
PATH:ko04925	53	8	2.02E-03	1.58E-02	Aldosterone synthesis and secretion
PATH:ko00909	42	7	2.12E-03	1.59E-02	Sesquiterpenoid and triterpenoid biosynthesis
PATH:ko04270	94	11	2.68E-03	1.92E-02	Vascular smooth muscle contraction
PATH:ko04916	85	10	3.95E-03	2.72E-02	Melanogenesis
PATH:ko04020	100	11	4.34E-03	2.87E-02	Calcium signaling pathway
PATH:ko04924	74	9	4.90E-03	3.12E-02	Renin secretion

PATH:ko04022	146	14	4.93E-03	3.03E-02	cGMP - PKG signaling pathway
PATH:ko04728	134	13	5.99E-03	3.55E-02	Dopaminergic synapse
PATH:ko04720	108	11	7.72E-03	4.42E-02	Long-term potentiation
PATH:ko04915	125	12	8.77E-03	4.87E-02	Estrogen signaling pathway
PATH:ko00906	96	10	9.28E-03	4.99E-02	Carotenoid biosynthesis
***A. marina* leaves, from 250 mM to 500 mM**					
PATH:ko00902	52	11	1.87E-06	2.66E-04	Monoterpenoid biosynthesis
PATH:ko04626	766	53	4.74E-06	3.36E-04	Plant-pathogen interaction
PATH:ko04110	222	22	1.81E-05	8.55E-04	Cell cycle
PATH:ko04914	107	13	1.20E-04	4.28E-03	Progesterone-mediated oocyte maturation
PATH:ko00950	138	15	1.34E-04	3.82E-03	Isoquinoline alkaloid biosynthesis
PATH:ko00940	326	26	1.37E-04	3.25E-03	Phenylpropanoid biosynthesis
PATH:ko04111	206	18	4.80E-04	9.73E-03	Cell cycle - yeast
PATH:ko03030	125	12	1.82E-03	3.22E-02	DNA replication
PATH:ko00941	126	12	1.94E-03	3.07E-02	Flavonoid biosynthesis
PATH:ko04075	735	42	2.22E-03	3.15E-02	Plant hormone signal transduction
PATH:ko00350	118	11	3.48E-03	4.49E-02	Tyrosine metabolism
PATH:ko00982	70	8	3.51E-03	4.15E-02	Drug metabolism - cytochrome P450
PATH:ko00909	42	6	3.64E-03	3.97E-02	Sesquiterpenoid and triterpenoid biosynthesis
PATH:ko00945	154	13	3.74E-03	3.79E-02	Stilbenoid, diarylheptanoid and gingerol biosynthesis
PATH:ko00943	43	6	4.10E-03	3.88E-02	Isoflavonoid biosynthesis
***A. marina* roots, from 0 mM to 250 mM**					
PATH:ko00196	18	5	6.34E-07	4.06E-05	Photosynthesis - antenna proteins
PATH:ko00940	326	13	2.03E-05	6.49E-04	Phenylpropanoid biosynthesis
PATH:ko00910	58	5	2.47E-04	5.27E-03	Nitrogen metabolism
PATH:ko03020	72	5	6.75E-04	1.08E-02	RNA polymerase
PATH:ko00941	126	6	1.46E-03	1.86E-02	Flavonoid biosynthesis
PATH:ko04724	88	5	1.67E-03	1.78E-02	Glutamatergic synapse
PATH:ko00230	350	10	2.33E-03	2.13E-02	Purine metabolism
PATH:ko00908	59	4	2.57E-03	2.06E-02	Zeatin biosynthesis
PATH:ko00830	69	4	4.53E-03	3.22E-02	Retinol metabolism
PATH:ko00604	78	4	6.98E-03	4.47E-02	Glycosphingolipid biosynthesis - ganglio series
***A. marina* roots, from 250 mM to 500 mM**					
PATH:ko00950	138	19	3.65E-10	5.04E-08	Isoquinoline alkaloid biosynthesis

PATH:ko00909	42	9	3.40E-07	2.35E-05	Sesquiterpenoid and triterpenoid biosynthesis
PATH:ko00941	126	14	1.10E-06	5.06E-05	Flavonoid biosynthesis
PATH:ko00500	604	33	3.83E-06	1.32E-04	Starch and sucrose metabolism
PATH:ko00592	87	11	4.38E-06	1.21E-04	alpha-Linolenic acid metabolism
PATH:ko00130	98	11	1.40E-05	3.22E-04	Ubiquinone and other terpenoid-quinone biosynthesis
PATH:ko00940	326	21	2.06E-05	4.07E-04	Phenylpropanoid biosynthesis
PATH:ko00196	18	5	3.93E-05	6.79E-04	Photosynthesis - antenna proteins
PATH:ko00943	43	7	4.70E-05	7.20E-04	Isoflavonoid biosynthesis
PATH:ko00945	154	13	5.20E-05	7.18E-04	Stilbenoid, diarylheptanoid and gingerol biosynthesis
PATH:ko00900	117	11	7.35E-05	9.22E-04	Terpenoid backbone biosynthesis
PATH:ko00040	262	17	1.13E-04	1.30E-03	Pentose and glucuronate interconversions
PATH:ko00624	84	9	1.21E-04	1.28E-03	Polycyclic aromatic hydrocarbon degradation
PATH:ko00902	52	7	1.64E-04	1.61E-03	Monoterpenoid biosynthesis
PATH:ko00363	75	8	2.95E-04	2.71E-03	Bisphenol degradation
PATH:ko04722	990	40	3.50E-04	3.02E-03	Neurotrophin signaling pathway
PATH:ko00980	77	8	3.53E-04	2.87E-03	Metabolism of xenobiotics by cytochrome P450
PATH:ko00965	18	4	6.19E-04	4.74E-03	Betalain biosynthesis
PATH:ko04620	887	36	6.26E-04	4.55E-03	Toll-like receptor signaling pathway
PATH:ko04064	864	34	1.46E-03	1.01E-02	NF-kappa B signaling pathway
PATH:ko02010	167	11	1.55E-03	1.02E-02	ABC transporters
PATH:ko00903	97	8	1.64E-03	1.03E-02	Limonene and pinene degradation
PATH:ko00901	40	5	2.00E-03	1.20E-02	Indole alkaloid biosynthesis
PATH:ko00270	179	11	2.67E-03	1.54E-02	Cysteine and methionine metabolism
PATH:ko04730	64	6	3.25E-03	1.79E-02	Long-term depression
PATH:ko00627	114	8	4.47E-03	2.37E-02	Aminobenzoate degradation
PATH:ko00982	70	6	5.07E-03	2.59E-02	Drug metabolism - cytochrome P450
PATH:ko00906	96	7	6.21E-03	3.06E-02	Carotenoid biosynthesis
PATH:ko00942	33	4	6.33E-03	3.01E-02	Anthocyanin biosynthesis
PATH:ko04713	77	6	8.03E-03	3.69E-02	Circadian entrainment
PATH:ko04626	766	28	9.61E-03	4.28E-02	Plant-pathogen interaction
PATH:ko00904	60	5	1.15E-02	4.96E-02	Diterpenoid biosynthesis

***S. alba* leaves, from 0 mM to 250 mM**

PATH:ko04111	177	23	5.13E-07	7.07E-05	Cell cycle - yeast
PATH:ko04110	202	24	1.51E-06	1.04E-04	Cell cycle
PATH:ko03030	91	14	1.22E-05	5.62E-04	DNA replication
PATH:ko00941	98	14	2.89E-05	9.98E-04	Flavonoid biosynthesis
PATH:ko04113	172	19	5.00E-05	1.38E-03	Meiosis - yeast
PATH:ko04914	87	11	5.96E-04	1.37E-02	Progesterone-mediated oocyte maturation
PATH:ko00940	288	23	1.09E-03	2.16E-02	Phenylpropanoid biosynthesis
PATH:ko04742	14	4	1.73E-03	2.98E-02	Taste transduction
PATH:ko00500	571	37	2.19E-03	3.36E-02	Starch and sucrose metabolism
PATH:ko00052	234	19	2.36E-03	3.25E-02	Galactose metabolism

***S. alba* leaves, from 250 mM to 500 mM**

PATH:ko00500	571	82	1.22E-08	2.55E-06	Starch and sucrose metabolism
PATH:ko00940	288	49	6.33E-08	6.62E-06	Phenylpropanoid biosynthesis
PATH:ko00052	234	41	3.27E-07	2.28E-05	Galactose metabolism
PATH:ko04722	824	99	2.58E-06	1.35E-04	Neurotrophin signaling pathway
PATH:ko04626	551	71	6.34E-06	2.65E-04	Plant-pathogen interaction
PATH:ko04075	684	83	1.12E-05	3.91E-04	Plant hormone signal transduction
PATH:ko00511	191	32	1.58E-05	4.72E-04	Other glycan degradation
PATH:ko00941	98	20	3.53E-05	9.22E-04	Flavonoid biosynthesis
PATH:ko00520	317	44	6.22E-05	1.44E-03	Amino sugar and nucleotide sugar metabolism
PATH:ko00100	60	14	1.14E-04	2.39E-03	Steroid biosynthesis
PATH:ko04620	731	83	1.17E-04	2.22E-03	Toll-like receptor signaling pathway
PATH:ko00073	70	15	1.84E-04	3.20E-03	Cutin, suberine and wax biosynthesis
PATH:ko04064	704	79	2.43E-04	3.91E-03	NF-kappa B signaling pathway
PATH:ko00010	302	40	3.51E-04	5.24E-03	Glycolysis / Gluconeogenesis
PATH:ko00053	97	17	8.62E-04	1.20E-02	Ascorbate and aldarate metabolism
PATH:ko00626	17	6	1.08E-03	1.41E-02	Naphthalene degradation
PATH:ko00592	88	15	2.25E-03	2.77E-02	alpha-Linolenic acid metabolism
PATH:ko00030	181	25	2.34E-03	2.72E-02	Pentose phosphate pathway
PATH:ko00943	40	9	2.45E-03	2.69E-02	Isoflavonoid biosynthesis
PATH:ko04915	134	20	2.47E-03	2.59E-02	Estrogen signaling pathway
PATH:ko04971	48	10	2.64E-03	2.63E-02	Gastric acid secretion
PATH:ko04540	126	19	2.78E-03	2.64E-02	Gap junction
PATH:ko00982	57	11	3.12E-03	2.83E-02	Drug metabolism - cytochrome P450
PATH:ko00900	100	16	3.18E-03	2.77E-02	Terpenoid backbone biosynthesis
PATH:ko00460	128	19	3.33E-03	2.78E-02	Cyanoamino acid metabolism

PATH:ko04015	103	16	4.29E-03	3.45E-02	Rap1 signaling pathway
PATH:ko04740	52	10	4.85E-03	3.76E-02	Olfactory transduction
PATH:ko04970	69	12	5.04E-03	3.76E-02	Salivary secretion
PATH:ko00980	61	11	5.36E-03	3.86E-02	Metabolism of xenobiotics by cytochrome P450
PATH:ko01220	30	7	5.89E-03	4.10E-02	Degradation of aromatic compounds
PATH:ko04024	136	19	6.50E-03	4.38E-02	cAMP signaling pathway
PATH:ko04530	99	15	7.04E-03	4.60E-02	Tight junction
PATH:ko00621	6	3	7.15E-03	4.53E-02	Dioxin degradation
PATH:ko00040	249	30	7.24E-03	4.45E-02	Pentose and glucuronate interconversions
PATH:ko04612	128	18	7.41E-03	4.43E-02	Antigen processing and presentation
PATH:ko04270	91	14	7.91E-03	4.59E-02	Vascular smooth muscle contraction
***S. alba* roots, from 0 mM to 250 mM**					
PATH:ko00195	75	25	8.69E-24	8.17E-22	Photosynthesis
PATH:ko00196	24	11	5.04E-13	2.37E-11	Photosynthesis - antenna proteins
PATH:ko00860	102	12	1.30E-06	4.08E-05	Porphyrin and chlorophyll metabolism
PATH:ko00940	288	20	2.65E-06	6.23E-05	Phenylpropanoid biosynthesis
PATH:ko00052	234	14	3.84E-04	7.22E-03	Galactose metabolism
PATH:ko00040	249	13	2.10E-03	3.29E-02	Pentose and glucuronate interconversions
PATH:ko00250	87	7	2.13E-03	2.86E-02	Alanine, aspartate and glutamate metabolism
PATH:ko00902	31	4	3.59E-03	4.22E-02	Monoterpenoid biosynthesis
PATH:ko04610	17	3	4.75E-03	4.96E-02	Complement and coagulation cascades
PATH:ko00460	128	8	5.09E-03	4.78E-02	Cyanoamino acid metabolism
***S. alba* roots, from 250 mM to 500 mM**					
PATH:ko04514	15	5	1.62E-07	8.26E-06	Cell adhesion molecules (CAMs)
PATH:ko04610	17	5	3.29E-07	8.38E-06	Complement and coagulation cascades
PATH:ko04390	105	8	5.25E-06	8.93E-05	Hippo signaling pathway
PATH:ko04670	45	5	5.28E-05	6.73E-04	Leukocyte transendothelial migration
PATH:ko00910	53	5	1.17E-04	1.19E-03	Nitrogen metabolism
PATH:ko00052	234	9	2.99E-04	2.54E-03	Galactose metabolism
PATH:ko04650	75	5	5.98E-04	4.36E-03	Natural killer cell mediated cytotoxicity
PATH:ko00250	87	5	1.17E-03	7.48E-03	Alanine, aspartate and glutamate metabolism

PATH:ko00941	98	5	1.99E-03	1.13E-02	Flavonoid biosynthesis
PATH:ko04015	103	5	2.47E-03	1.26E-02	Rap1 signaling pathway
PATH:ko00520	317	9	2.51E-03	1.17E-02	Amino sugar and nucleotide sugar metabolism
PATH:ko00960	78	4	5.51E-03	2.34E-02	Tropane, piperidine and pyridine alkaloid biosynthesis
PATH:ko04810	180	6	6.06E-03	2.38E-02	Regulation of actin cytoskeleton
PATH:ko04145	251	7	8.10E-03	2.95E-02	Phagosome
PATH:ko00950	100	4	1.30E-02	4.42E-02	Isoquinoline alkaloid biosynthesis
PATH:ko00830	55	3	1.35E-02	4.32E-02	Retinol metabolism
PATH:ko00945	105	4	1.53E-02	4.59E-02	Stilbenoid, diarylheptanoid and gingerol biosynthesis
PATH:ko00500	571	11	1.54E-02	4.37E-02	Starch and sucrose metabolism
PATH:ko00350	111	4	1.84E-02	4.93E-02	Tyrosine metabolism
PATH:ko00220	62	3	1.86E-02	4.75E-02	Arginine biosynthesis

Supplementary References

92. Cho YS, Hu L, Hou H *et al.* The tiger genome and comparative analysis with lion and snow leopard genomes. *Nat Commun* 2013;**4**:2433.

93. Kent WJ. BLAT - The BLAST-like alignment tool. *Genome Res* 2002;**12**:656–64.

94. Jones P, Binns D, Chang HY *et al.* InterProScan 5: Genome-scale protein function classification. *Bioinformatics* 2014;**30**:1236–40.

95. Huang S, Ding J, Deng D *et al.* Draft genome of the kiwifruit Actinidia chinensis. *Nat Commun* 2013;**4**:2640.

96. Xu X, Pan S, Cheng S *et al.* Genome sequence and analysis of the tuber crop potato. *Nature* 2011;**475**:189–95.

97. Hoagland DR, Arnon DI. The water-culture method for growing plants without soil. *Circular. California Agricultural Experiment Station, 347(2nd Edit)*. 1950.

98. Benjamini Y, Hochberg Y. Controlling the False Discovery Rate: A Practical and Powerful Approach to Multiple Testing. *J R Stat Soc Ser B* 1995;**57**:289–300.

99. Posmyk MM, Kontek R, Janas KM. Antioxidant enzymes activity and phenolic compounds content in red cabbage seedlings exposed to copper stress. *Ecotoxicol Environ Saf* 2009;**72**:596–602.

100. Waśkiewicz A, Muzolf-Panek M, Goliński P. Phenolic Content Changes in Plants Under Salt Stress. In: Ahmad P, Azooz MM, Prasad MN V (eds.). *Ecophysiology and Responses of Plants under Salt Stress*. New York, NY: Springer New York, 2013, 283–314.

101. Suyama M, Torrents D, Bork P. PAL2NAL: robust conversion of protein sequence alignments into the corresponding codon alignments. *Nucleic Acids Res* 2006;**34**:W609–12.

102. Edgar RC. MUSCLE: multiple sequence alignment with high accuracy and high throughput. *Nucleic Acids Res* 2004;**32**:1792–7.

103. Ho SYW. The changing face of the molecular evolutionary clock. *Trends Ecol Evol* 2014;**29**:496–503.

104. Yim H-SS, Cho YS, Guang X *et al.* Minke whale genome and aquatic adaptation in cetaceans. *Nat Genet* 2014;**46**:88–92.

105. Frantz LAF, Schraiber JG, Madsen O *et al.* Genome sequencing reveals fine scale diversification and reticulation history during speciation in Sus. *Genome Biol* 2013;**14**:R107.

106. Bremer K, Friis EM, Bremer B. Molecular phylogenetic dating of asterid flowering plants shows early cretaceous diversification. *Syst Biol* 2004;**53**:496–505.

107. Davis CC, Webb CO, Wurdack KJ *et al.* Explosive radiation of Malpighiales supports a mid-

Cretaceous origin of modern tropical rain forests. *Am Nat* 2005;**165**:E36–65.

108. Xi Z, Ruhfel BR, Schaefer H *et al.* Phylogenomics and a posteriori data partitioning resolve the Cretaceous angiosperm radiation Malpighiales. *Proc Natl Acad Sci* 2012;**109**:17519–24.

109. Muller J. Fossil pollen records of extant angiosperms. *Bot Rev* 1981;**47**:1–142.

110. Graham A. Paleobotanical Evidence and Molecular Data in Reconstructing the Historical Phytogeography of Rhizophoraceae. *Ann Missouri Bot Gard* 2006;**93**:325–34.

111. Sytsma KJ, Litt A, Zjhra ML *et al.* Clades, Clocks, and Continents: Historical and Biogeographical Analysis of Myrtaceae, Vochysiaceae, and Relatives in the Southern Hemisphere. *Int J Plant Sci* 2004;**165**:S85–105.

112. Wikström N, Savolainen V, Chase MW. Evolution of the angiosperms: Calibrating the family tree. *Proc R Soc B Biol Sci* 2001;**268**:2211–20.

113. Lakhanpal RN, Guleria JS. Leaf impressions from the Eocene of Kachchh, western India. *Palaeobotanist* 1981;**28**–**29**:353–73.

114. Sanderson MJ. r8s: inferring absolute rates of molecular evolution and divergence times in the absence of a molecular clock. *Bioinformatics* 2003;**19**:301–2.

115. Wang K, Wang Z, Li F *et al.* The draft genome of a diploid cotton Gossypium raimondii. *Nat Genet* 2012;**44**:1098–103.

116. Al-Mssallem IS, Hu S, Zhang X *et al.* Genome sequence of the date palm Phoenix dactylifera L. *Nat Commun* 2013;**4**:2274.

117. Vanneste K, Baele G, Maere S *et al.* Analysis of 41 plant genomes supports a wave of successful genome duplications in association with the Cretaceous − Paleogene boundary Analysis of 41 plant genomes supports a wave of successful genome duplications in association with the Cretaceous – Pale. *Genome Res* 2014;**32**:1334–47.

118. Richards TA, Jones MDM, Leonard G *et al.* Marine Fungi: Their Ecology and Molecular Diversity. *Ann Rev Mar Sci* 2012;**4**:495–522.

119. Wang Y, Sheng HF, He Y *et al.* Comparison of the levels of bacterial diversity in freshwater, intertidal wetland, and marine sediments by using millions of illumina tags. *Appl Environ Microbiol* 2012;**78**:8264–71.

120. Bandaranayake WM. Bioactivities, bioactive compounds and chemical constituents of mangrove plants. *Wetl Ecol Manag* 2002;**10**:421–52.

121. Bandaranayake WM. Survey of mangrove plants from Northern Australia for phytochemical constituents and UV absorbing compounds. *Curr Top Phytochem* 1995;**14**:69–78.

122. Langmead B, Salzberg SL. Fast gapped-read alignment with Bowtie 2. *Nat Methods* 2012;**9**:357–359.

123. Morley RJ. Origin and Evolution of Tropical Rain Forests. 2000;**15**:378.

124. Mullert JAN. Significance of Fossil Pollen for Angiosperm History. *Ann Missouri Bot Gard* 1984;**71**:419–43.

125. Amborella Genome Project. The Amborella Genome and the Evolution of Flowering Plants. *Science* 2013;**342**:1241089.

126. D'hont A, Denoeud F, Aury JM *et al.* The banana (Musa acuminata) genome and the evolution of monocotyledonous plants. *Nature* 2012;**488**:213–7.

127. Iorizzo M, Ellison S, Senalik D *et al.* A high-quality carrot genome assembly provides new insights into carotenoid accumulation and asterid genome evolution. *Nat Genet* 2016;**48**:657–66.

128. Argout X, Salse J, Aury J-M *et al.* The genome of Theobroma cacao. *Nat Genet* 2011;**43**:101–8.

129. Huang S, Li R, Zhang Z *et al.* The genome of the cucumber, Cucumis sativus L. *Nat Genet* 2009;**41**:1275–81.

130. Al-Dous EK, George B, Al-Mahmoud ME *et al.* De novo genome sequencing and comparative genomics of date palm (Phoenix dactylifera). *Nat Biotechnol* 2011;**29**:521–7.

131. Wang J, Zhang G, Liu X *et al.* Genome sequence of foxtail millet (Setaria italica) provides insights into grass evolution and biofuel potential. *Nat Biotechnol* 2012;**30**:549–54.

132. Vanburen R, Bryant D, Edger PP *et al.* Single-molecule sequencing of the desiccation-tolerant grass Oropetium thomaeum. *Nature* 2015;**527**:508–11.

133. Ming R, VanBuren R, Wai CM *et al.* The pineapple genome and the evolution of CAM photosynthesis. *Nat Genet* 2015;**47**:1435–42.

134. Olsen JL, Rouzé P, Verhelst B *et al.* The genome of the seagrass Zostera marina reveals angiosperm adaptation to the sea. *Nature* 2016;**530**:331–5.

135. Varshney RK, Chen W, Li Y *et al.* Draft genome sequence of pigeonpea (Cajanus cajan), an orphan legume crop of resource-poor farmers. *Nat Biotechnol* 2012;**30**:83–9.

136. Shulaev V, Sargent DJ, Crowhurst RN *et al.* The genome of woodland strawberry (Fragaria vesca). *Nat Genet* 2011;**43**:109–16.

137. Dassanayake M, Oh D-H, Haas JS *et al.* The genome of the extremophile crucifer Thellungiella parvula. *Nat Genet* 2011;**43**:913–8.

138. Wu HJ, Zhang Z, Wang JY *et al.* Insights into salt tolerance from the genome of Thellungiella salsuginea. *Proc Natl Acad Sci* 2012;**109**:12219–24.
